# Synthesis of Alkyl/Aryloxymethyl Derivatives of 1,2,4-Triazole-3-Carboxamides and Their Biological Activities

**DOI:** 10.3390/molecules29204808

**Published:** 2024-10-11

**Authors:** Ekaterina A. Mikhina, Daria V. Stepanycheva, Varvara P. Maksimova, Olga N. Sineva, Natalia N. Markelova, Lyubov E. Grebenkina, Ekaterina A. Lesovaya, Marianna G. Yakubovskaya, Andrey V. Matveev, Ekaterina M. Zhidkova

**Affiliations:** 1 Lomonosov Institute of Fine Chemical Technologies, MIREA-Russian Technological University, 86 Vernadsky Prospekt, Moscow 119571, Russia; mik.hi@mail.ru (E.A.M.); legrebenkina@mail.ru (L.E.G.); 4motya@gmail.com (A.V.M.); 2 Department of Chemical Carcinogenesis, N.N. Blokhin Russian Cancer Research Center, Ministry of Health of Russia, 24 Kashirskoe Shosse, Moscow 115478, Russia; darya.stepanycheva@yandex.ru (D.V.S.); lavarvar@gmail.com (V.P.M.); lesovenok@yandex.ru (E.A.L.); mgyakubovskaya@mail.ru (M.G.Y.); 3 Gause Institute of New Antibiotics, 11 Bolshaya Pirogovskaya St., Moscow 119021, Russia; olga.sineva81@yandex.ru (O.N.S.); nathanmrk82@gmail.com (N.N.M.); 4 Faculty of Oncology, I.P. Pavlov Ryazan State Medical University, Ministry of Health of Russia, 9 Vysokovol’tnaya St., Ryazan 390026, Russia; 5 Laboratory of Single Cell Biology, Friendship University of Russia, 6 Miklukho-Maklaya St., Moscow 117198, Russia

**Keywords:** 1,2,4-triazole-3-carboxamides, ribavirin, acute lymphoblastic leukemia, chronic myeloid leukemia, cancer treatment, antimicrobial effect

## Abstract

Ribavirin and its analogues exhibit an in vitro antiproliferative effect in cancer cells. In this work, we studied the biological activities of a number of alkyl/aryloxymethyl derivatives of ribavirin’s aglycon—1,2,4-triazole-3-carboxamide. Alkyl/arylxymethyl derivatives of 1,2,4-triazole-3-carboxamide with substitutions at the fifth or first position of the triazole ring, were synthesized and their antiproliferative and antimicrobial effects were assessed. For both series, the presence of an antiproliferative effect was investigated, and 1-alkyl/aryloxymethyl derivatives were shown an antimicrobial potential against a Gram-positive bacteria *Micrococcus luteus* and Gram-negative bacterium *Pseudomonas aeruginosa*. The obtained results showed that the n-decyloxymethyl derivatives induced leukemia cell death at low micromolar concentrations. We confirmed that n-decyloxymethyl derivatives of ribavirin inhibited the cell cycle progression and induced an accumulation of leukemia cells in the subG1-phase. The molecular docking results suggest that alkyl/aryloxymethyl derivatives may act by inhibiting translation initiation, due to interference with eIF4E assembly. The outcome results revealed that active derivatives (1- or 5-n-decyloxymethyl-1,2,4-triazole-3-carboxamides) can be considered as a lead compound for anticancer treatments.

## 1. Introduction

Synthetic analogues of natural nucleosides have a wide spectrum of applications as antiviral, anticancer and antibacterial compounds. An example is ribavirin (1-(β-D-ribofuranosyl)-1,2,4-triazole-3-carboxamide, **1a**), which is not only used as an antiviral drug but also shows significant potential in cancer therapy, including blood cancer [1,2], as well as a moderate antimicrobial effect [3]. Thus, the multivalency of ribavirin applications makes it an interesting parent structure for new drug candidate design.

However, numerous studies showed that ribavirin has teratogenic and genotoxic effects, which significantly limits its therapeutic application [4]. This feature is common to most of the other nucleoside analogues, probably due to their involvement in the basic metabolic pathways. It is therefore of interest to search for alternatives of compound **1a** that have meaningful structural differences from nucleosides. Some ribavirin analogues with different fragments at the 1- and/or 5-positions of triazole ring show specific activity in the cells of several cancer types. For example, several triazoles with the biphenyl group in 1-position are cytotoxic in breast cancer cells in micromolar concentrations [5]. Several hybrid molecules containing secoestroides and triazole fragments demonstrate cytotoxicity in cervical cancer and breast cancer cell lines [6,7,8]. However, despite significant progress in the bioorganic chemistry of nucleoside analogues, identification of the most promising ways of their modification to maintain high efficacy and simultaneously reduce the significant side effects is highly in demand. Therefore, the traditional approach in the synthesis of new agents, followed by the identification of their biological effects, remains relevant to the solution of this problem.

Recently, we showed antiproliferative effects of the ribavirin aglycon in acute lymphoblastic leukemia and chronic myeloid leukemia cell lines [9]. We obtained ribavirin analogues through a replacement of a ribavirin ribose fragment with tetrahydropyran and tetrahydrofuran groups in 5- and 1-positions of the triazole ring. We showed the accumulation of cancer cells treated with 1,2,4-triazol-3-carboxamides in the G1 phase of the cell cycle and the induction of caspase-3 cleavage, resulting in apoptosis in leukemia cells [9]. Therefore, we assume that the ribavirin analogues with non-sugar fragments in 1- or 5-position may act like nucleoside analogues.

The present study was undertaken to prepare a series of alkyl/aryloxymethyl derivatives of 1,2,4-triazole-3-carboxamide as well as an evaluation of its anticancer actions in leukemia cell lines and antimicrobial activities in Gram-positive and Gram-negative bacteria.

## 2. Results and Discussions

Biologically active nucleoside analogues were obtained by replacing the carbohydrate fragment with its acyclic analogue, mostly with the exclusion of some hydroxyl groups. In the case of ribavirin analogues obtained using this approach, there should be several distinguished derivatives with an acyclic carbohydrate fragment and substitution at the position 1—1,2,4-triazole-3-carboxamides **1b**–**f**. Another approach to the nucleoside analogue modification was the replacement of the N-glycoside bond with a C-glycoside for easier exclusion of the biolabile bond from the structure of the analogue without a change in the main pharmacophore fragments. The ribavirin C-nucleoside analogue **2a** was obtained by this method applied to modification **1a** [10,11]. The authors noted the importance of the hydroxyethoxymethyl fragment presence in the structure of the molecule for an acyclic analogue to retain antiviral activity. Later, the other 1-hydroxyethoxymethyl derivatives of 5-substituted 1,2,4-triazole-3-carboxamides were synthesized, some of which showed antiviral activity against hepatitis C virus and an anticancer effect on cell models [9,12,13,14]. Thus, the reduction of the carbohydrate to a hydroxyethoxymethyl moiety does not prevent the molecular recognition of ribavirin analogues by a significant number of enzymes.

**Figure 1:**
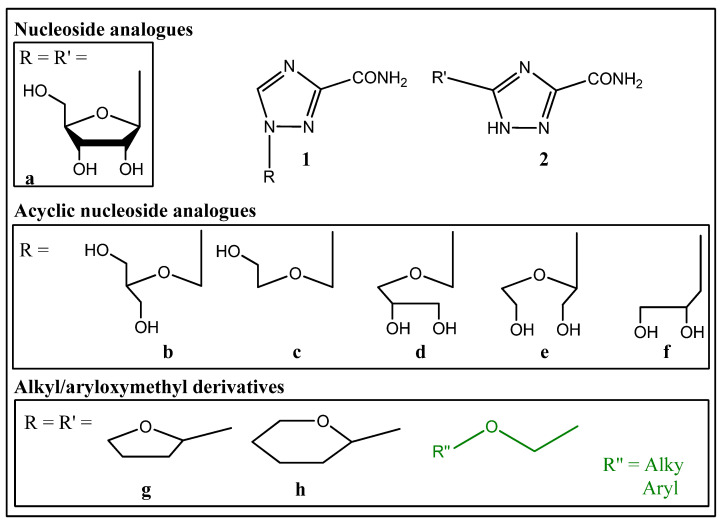
Ribavirin **1a** and its analogues.

In our previous work on modification of the carbohydrate moiety, we showed that the derivatives of 1,2,4-triazole-3-carboxamide **1g**, **h** and **2g**, **h** substituted at position 5 as well as at position 1 of the triazole ring with 2-tetrahydrofuranyl or 2-tetrahydropyranyl groups which can be considered as analogs of the carbohydrate backbone, devoid of hydroxyl groups inhibit the proliferation of chronic myeloid (K562) and acute lymphoblastic (CCRF-SB) leukemia cells [9] (Figure 1). However, the detailed mechanism of the biological activity of compounds **1g**, **h** and **2g**, **h** remains unclear. Therefore, we assumed that the ribavirin analogues with a non-sugar fragment in the 1- or 5-position may, in some aspects, act like a nucleoside analogue, in particular revealing anticancer and antimicrobial effects.

### 2.1. Synthesis

Two series of compounds were synthesized: 5-alkyl/aryloxymethyl-1,2,4-triazole-3-carboxamides **6a**–**e**, **g**, **h**, **k** and 1-alkyl/aryloxymethyl-1,2,4-triazole-3-carboxamides 1c and **11a**–**k**. The methods of their synthesis differed for each series: the introduction of a 5-alkyl/aryloxymethyl fragment was carried out by the cyclization of a triazole fragment, while the introduction of 1-alkyl/aryloxymethyl fragment was carried out by the alkylation of methyl 1,2,4-triazole-3-carboxylate.

**Figure 2:**
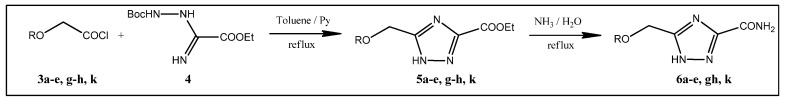
5-alkyl/aryloxymethyl-1,2,4-triazole-3-carboxamide preparation.

5-alkyl/aryloxymethyl analogues of ribavirin **6a**–**e**, **g**, **h**, **k** were synthesized by the previously described method [15], consisting in the ammonolysis of ethyl esters of 5-alkyl/aryloxymethyl-1,2,4-triazole-3-carboxylic acids **5a**–**e**, **g**, **h**, **k** obtained by the treatment of β-N-t-butyloxycarbonyloxalamidrazone 11 with alkyl/aryloxyacetyl chlorides, followed by a one-pot cyclization of intermediates (Figure 2). Target compounds **6** yields out of **4** are presented in Table 1.

In the case of an alkyl/aryloxymethyl substituent introduction into the 1,2,4-triazole ring, which is a necessary step in compound **11** synthesis, an alkylation can occur at any nitrogen of the triazole ring, leading to a formation of three regioisomers [16,17]. According to sources in the literature, the method of introducing an alkyl/aryloxymethyl substituent via triazole carboxylic acid ester N-silyl derivatives by alkyl/aryloxymethylacetates is considered as the most regioselective, e.g., the only product of such alkylation for methyl 1,2,4,-triazole-3-carboxylate is methyl 1-alkoxymethyl 1,2,4-triazole-3-carboxylate [17].

**Figure 3:**
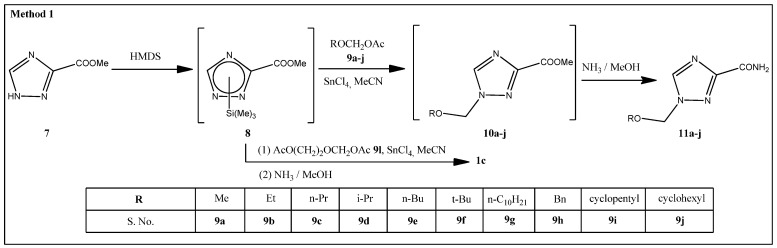
Introduction of a 1-alkoxymethyl moiety.

In our study, methyl 1-alkyloxymethyl-1,2,4-triazole-3-carboxylates **10a**–**j** were prepared in two steps: first, we obtained the silyl derivatives of methyl 1,2,4-triazole-3-carboxylate **7** by its treatment with 1,1,1,1,3,3,3,3-hexamethyldisilazane (HMDS) followed by an addition of alkyloxymethylacetates **9a**–**j** in the presence of Lewis acid tin tetrachloride (Figure 3). Compounds **9a**–**j** were synthesized from dialkoxymethanes **14a**–**j** [18], which in turn, were obtained by a known method [19]. In the case of 1-([2-hydroxyethoxy]methyl)-1,2,4-triazole-3-carboxamide **1c**, compound **8** was treated with [2-(acetoxy)ethoxy]methyl acetate **9l** [20] obtained from 1,3-dioxalane, the acetate protecting group of the ethyloxymethyl moiety that was removed by ammonolysis. Methyl 1-methoxymethyl-1,2,4-triazole-3-carboxylate **10a** was isolated by column chromatography resulting in a 38.5% yield. The esters **10b**–**j** were used at the next stage without further purification. The amides **11a**–**j** were obtained by ammonolysis of the esters **10a**–**j** (Figure 3) and were purified through recrystallization from an ethanol–ethyl acetate mixture in yields ranging from 34 to 91% (Table 2 shows the yield of compounds **11** out of **7** and the yield of **1c** out of **7**).

**Figure 4:**
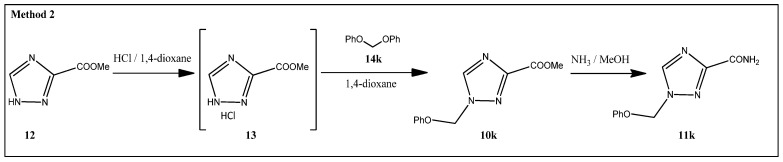
Introduction of a 1-phenoxymethyl moiety.

1-(Phenoxymethyl)-1,2,4-triazole-3-carboxamide **11k** was prepared using diphenoxymethane **14k** that was synthesized according to a procedure described in the literature [21]. Methyl 1,2,4-triazole-3-carboxylate hydrochloride **13** was treated with **14k** to give the ester 1-(phenoxymethyl)-1,2,4-triazole-3-carboxylate **10k** followed by its ammonolysis (Figure 4). Amide **11k** was purified in a similar way to the previous amides **16a**–**j** manner, resulting in a 52% yield.

**Figure 5:**
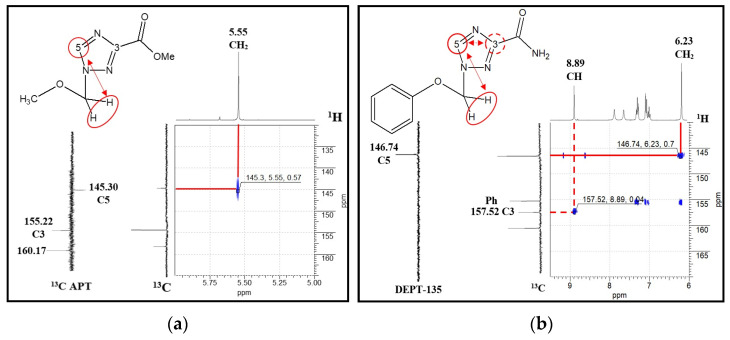
APT, DEPT-135 and ^1^H-^13^C HMBC NMR spectra fragments: (**a**) methyl 1-(methoxymethyl)-1,2,4-triazole-3-carboxylate and (**b**) 1-(phenoxymethyl)-1,2,4-triazole-3-carboxamide.

The structures of the obtained compounds were established using a set of physicochemical methods: ^1^H and ^13^C NMR, HRMS. A combination of APT and ^1^H-^13^C HMBC NMR experiments was used to establish the position of the alkyl/aryloxymethyl substituent in the case of compound **10a**. We identified structures **11b**–**j** and **1c** as the position 1 isomers based on the similarity of their NMR characteristics to those of **11a** (**11a** was obtained by ammonolysis of **10a**). In the case of **11k**, the position of the phenoxymethyl radical was established by DEPT-135 and ^1^H-^13^C HMBC NMR (Figure 5).

### 2.2. In Vitro Studies

#### 2.2.1. Anti-Cancer Activity In Vitro

The in vitro cytotoxic activities of the synthesized compounds were evaluated on CCRF-SB and K562 cells using an MTT assay. Compounds **11g** and **6g** showed the highest cytotoxic activity in the leukemia cell lines after a 24 h exposition, as shown in Table 3. The CC_50_ values for **11g** were calculated as 13.6 ± 0.3 µM in the K562 cells and 112 ± 19 µM in the CCFR-SB cells, respectively. CC_50_ for 6g were 391 ± 15 µM in the K562 cell line.

The CC_50_ values for **11g** were about 20-fold lower than ribavirin and about 4-fold lower than Cyt in the K562 cells. Most notably, one of the derivatives **6g** showed activity against acute lymphoblastic leukemia (CCRF-SB), an aggressive form of pediatric leukemia.

For other compounds, CC_50_ values were not determined. However, a number of compounds showed a dose–response cytostatic effect on leukemia cells at 72 h of exposure, assuming that newly synthesized 1,2,4-triazole-3-carboxamide derivatives may possess an antiproliferative effect associated with low toxicity. The MTT assay after 72 h revealed the cytotoxic effects of **11e**, **6g**, and **6k** in the acute lymphoblastic leukemia cell line and cytotoxic effects of **11i**, **11h**, **11f**, **6e** and **6k** in chronic myeloid leukemia cells. Consequently, compounds **11e**, **11g**, **11i**, **11h**, **11f**, **6e**, **6g** and **6k** were selected to study its antiproliferative activity.

**Figure 6:**
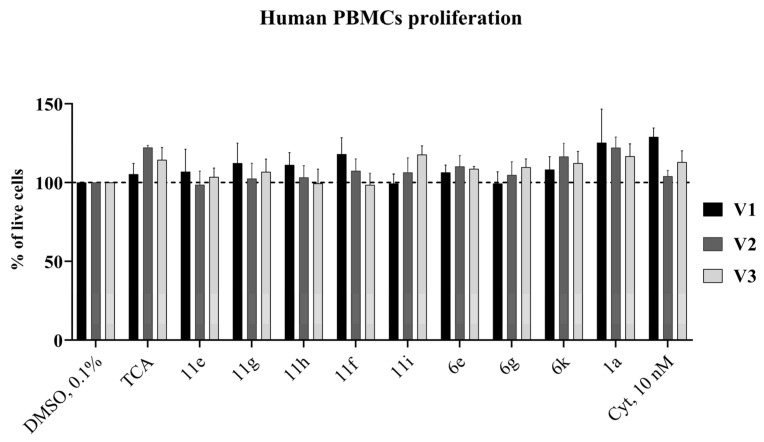
Effect of compounds on human PBMC proliferation. Cells were treated with the DMSO, Cyt, **1a** or its derivatives for 72 h and then were counted using trypan blue exclusion test. V—volunteer. All data are expressed as percent of DMSO treated control. Significant differences were analyzed by the one-way ANOVA test.

To evaluate the effect of compounds on non-transformed cells, human peripheral blood mononuclear cells (PBMC) were isolated from the whole blood of three healthy volunteers. Then, PBMCs were incubated for 72 h with 1,2,4-triazole-3-carboxamide derivatives in the highest concentrations (500 µM). The normal cells were less sensitive to compounds than cancer cells (Figure 6), highlighting the selectivity of action of novel compounds on malignant cells.

**Figure 7:**
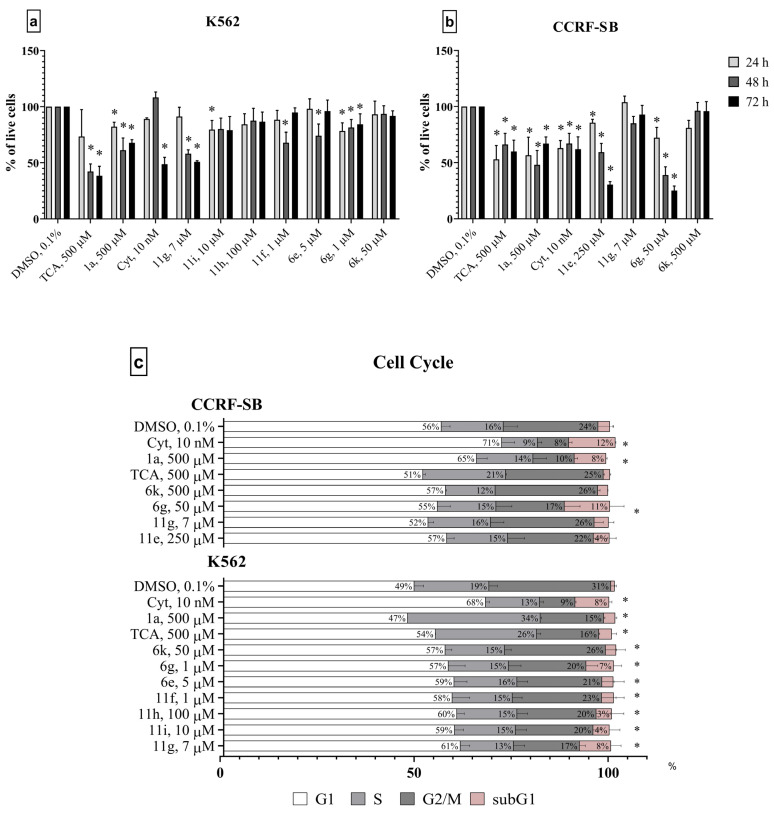
(**a**,**b**) Antiproliferative effects of compounds in CCRF-SB and K562 cancer cells. The cells were cultured with the solvent (DMSO), cytarabine (Cyt), or ribavirin (**1a**), or its derivatives. Cells were stained with trypan blue and counted after 24, 48 and 72 h of the treatment. (**c**) Effect of selected compounds on cell cycle progression in K562 and CCRF-CEM cells after 72 h of incubation with the DMSO, Cyt, **1a** or its derivatives. Cells were fixed with ethanol and then stained with propidium iodide and analyzed by flow cytometry. All data are expressed as percent of DMSO treated control. Significant differences were analyzed by the one-way ANOVA test. *—significant differences from the control (*p* < 0.05).

We conducted an in vitro trypan blue exclusion assay to test the cytostatic (antiproliferative) activity of newly synthesized 1,2,4-triazole-3-carboxamide derivatives. The cells were incubated with the active compounds for 24–72 h at concentrations equal to the calculated CC_20_ value. Compound **11g** significantly reduced cell proliferation in chronic myeloid leukemia cells and caused minimal cell death in the acute lymphoblastic leukemia cell line. Compounds **6g** and **11e** showed dose-depending antiproliferative action in the CCRF-SB cell line (Figure 7a,b).

To investigate the mechanism underlying the cell growth inhibition induced by 1,2,4-triazole-3-carboxamide derivatives, the cell cycle profile was analyzed by flow cytometry with PI staining. The K562 and CCRF-SB cells were exposed to compounds for 72 h. In Figure 7c, it is demonstrated that the compound **6g** caused an increase in the cell population in the G0 phase, indicating cell death in CCRF-SB culture. The population of the G2/M and S phases of CCRF-SB cells reduced after the treatment with 7 µm **6g** compared with control. At the same time, all compounds increased the accumulation of cells in the G1 phase and caused a decrease in the percentage of cells in the G2/M phase in the K562 cell line. The treatment of K562 cells with 7 µM **11g** significantly reduced the fraction of cells in the S and G2/M phases and increased the proportion of cells in the G1 phase. Furthermore, **6g** and **11g** significantly increased the accumulation of cells in the subG1 phase corresponding to apoptotic cells by 7 and 8 times, respectively, in K562 cells. The ability of compounds **6g** and **11g** to induce cell death is concordant with the cytotoxicity determined by the MTT assay.

#### 2.2.2. Antimicrobial Effect Studies

The multivalency of the biological effects of ribavirin—the parent structure of the 1- or 5-alkyl/aryloxymethyl-1,2,4-triazole-3-carboxamides—prompted us to study their antimicrobial properties. The antimicrobial potential of compounds **6a**–**e**, **g**, **h**, **k** and **11a**–**k** was investigated in comparison with that of reference molecules **1a** and **1c** against the following series of microorganisms: Micrococcus luteus ATCC 9341, Staphylococcus aureus INA 00985, Pseudomonas aeruginosa ATCC 27853 and Candida albicans ATCC 14053 on an agarose nutrient medium at concentrations of 25 mM (Table 4).

Compounds **6a**–**e**, **g**, **h**, **k** have not showed any suppression of microorganism growth. Compounds **11i**, **j** and **1c** showed bacteriostatic activity against the Gram-positive organism *M. luteus* but not against *S. aureus*, in contrast to ribavirin **1a**, which showed no antimicrobial activity against such organisms. In the case of the Gram-negative microorganism *P. aeruginosa*, moderate activity compared to that of **1a** was observed for compound **11c**. In relation to *C. albicans*, the studied compounds **1c**, **11** showed no activity, and ribavirin **1a** showed the highest activity.

### 2.3. Molecular Docking

Due to the revealed effects of the synthesized compounds **6a**–**e**, **g**, **h**, **k** and **11a**–**k**, **1c** towards acute lymphoblastic leukemia and chronic myeloid leukemia cell lines, we became interested in trying to assume the mechanism underlying the action. As known, ribavirin undergoes phosphorylation in cells to form ribavirin 5-monophosphate (RMP) [22]. A number of cellular targets were shown for RMP, including inosine-5′-phosphate dehydrogenase (IMPDH) and the eukaryotic translation initiation factor 4E (elF4F) [23,24,25]. Inhibition of IMPDH occurs due to an insertion into the inosine monophosphate binding site, and elF4E is presumably, according to various sources, either due to insertion into the 5′-cap mRNA binding site or by interfering with the assembly of protein subunits of the factor [26,27,28,29,30]. Oxymethyl derivatives of TCA **6a**–**e**, **g**, **h**, **k** and **11a**–**k** do not have a hydroxyl substituent and therefore cannot be phosphorylated. Therefore, the main opportunity for them to participate in these biochemical pathways is to block the interaction of the eIF4E and eIF4G subunits of factor 4E, by binding to at least one of them [31]. An example of such an effect of low-molecular-weight compounds is an inhibitor of this interaction, 4EGI-1 [32]. 4EGI-1 disrupts the eIF4E/eIF4G association in vitro and in vivo and reduces the viability of a wide range of cancer cells, such as breast cancer and multiple myeloma [33]. 4EGI-1 inhibits tumour growth in in vivo models of acute myeloid leukemia and chronic lymphocytic leukemia. [34,35,36,37]. Therefore, we used a region of the protein surface characteristic of 4EGI-1 binding as a target for modelling.

The structure of the elF4E protein (PDB: 4TPW) and the structures of low-molecular-weight ligands **1g**, **h** and **2g**, **h**, **6**, **11** optimized with an OPLS3e force field were used for molecular docking, which was performed in Schrodinger Maestro.

**Figure 8:**
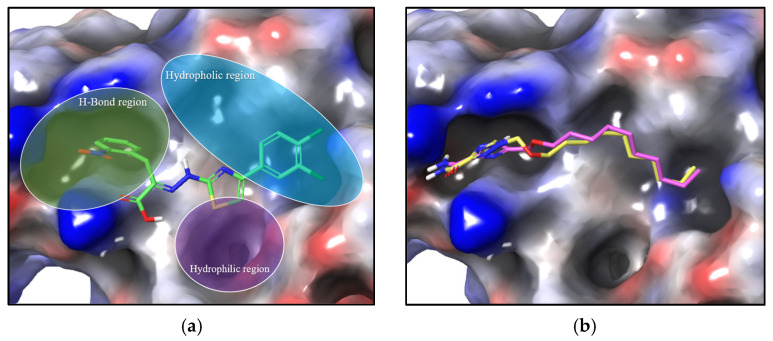
Binding of test compounds to protein elF4E: (**a**) known inhibitor 4EGI-1—green; (**b**) **6g**—pink, **11g**—yellow.

According to the simulation results, several newly synthesized compounds **6** and **11** as well as ribavirin **1a**, its N-alkyl/aryloxymethyl analogues **1g**, **1h** and its C-alkyl/aryloxymethyl analogues **2g**, **2h** demonstrate a preferential localization in the binding site of the known inhibitor 4EGI-1. DockingScore was not representative, as the best values of −5.5 were shown by **1a**, while the known inhibitor 4EGI-1 gave −3.5, which was shown earlier by other authors [32]. The binding site responsible for the eIF4E/eIF4G association (hereinafter referred to as the site) contingently can be divided into three regions: hydrophilic, hydrophobic and H-bond regions. Binding of the known inhibitor 4EGI-1 occurs in the first and third regions of the site (Figure 8a). The lipophilic region is formed by the following amino acid residues: Leu75, Hie78, Ile79, Ser82, Ser83 and Tyr91, H-bond region includes Ala58, Asn59, Lys54, Arg61, Ile63, Lys49.

The most active compound **11g** and well-known inhibitor 4EGI-1 binds to the same eIF4E site according to our simulation results. Moreover, the binding of **11g** occurs mainly with the same regions as for 4EGI-1. In this case, an increase in the proportion of lipophilic interactions is observed due to the binding to Ile63 and Leu45. In addition, in the case of **11g**, a π-interaction of the triazole ring with Arg61 is additionally observed (the thiazole fragment of 4EGI-1 also has a π-interaction, but with Phe47). Compound **6g**, which showed moderate activity, exhibited a binding profile similar to **11g** (Figure 8b) (see Supplementary Materials), but with a reduced proportion of lipophilic interactions.

Thus, as a result of the modelling, it was possible to trace the correlation between the pattern of lipophilicity together with the location of alkoxymethyl analogues of ribavirin on the surface of eIF4E and their in vitro toxicity to cancer cells. This pattern suggests that a possible mechanism of action of the synthesized compounds may be associated with the inhibition of RNA translation due to disruptions in the assembly of the elF4F complex.

## 3. Materials and Methods

### 3.1. Synthetic Section

#### 3.1.1. 5-(n-Propoxymethyl)-1,2,4-triazole-3-carboxamide **6c**

A total of 1.88 g (11.02 mmol) of n-propyloxyacetyl chloride was added dropwise to a suspension of 1.19 g (5.15 mmol) of β-N-(t-butyloxycarbonyl)ethyloxalamidrazone **4** in anhydrous pyridine while cooling the reaction mixture to 0 °C. The reaction mass was brought to boiling point and stirred for 20 h. After the reaction was completed (control was carried out using TLC), the solvent was removed on a vacuum rotary evaporator. The residue was dissolved in a 1 M aqueous HCl solution and extracted 3 times with ethyl acetate in equal portions. The organic phases were combined and dried using Na_2_SO_4_, and the solvent was removed on a vacuum rotary evaporator. The crude product **5c** was isolated by flash chromatography on silica gel using chloroform/methanol system (with a methanol gradient from 0 to 7%) as an eluent. The crude product **5c** was dissolved in 2 mL of a 10 M ammonia methanol solution and heated to boiling under reflux for 12 h, and the solvent was removed using a vacuum rotary evaporator. Residue was suspended in anhydrous acetone, filtered and dried in a desiccator under reduced pressure above NaOH for 12 h to yield 0.23 g (24%) **6c** as white crystals.

R_f_ = 0.61 (1% CH_3_OH in CHCl_3_), mp 110–111 °C. ^1^H NMR spectrum (DMSO-d_6_) δ: 0.83 (t, 3H, *J* = 7.09, CH_3_CH_2_CH_2_); 1.50 (se, 2H, *J* = 7.09, CH_3_CH_2_CH_2_); 3.40 (t, 2H, *J* = 6.09, CH_3_CH_2_CH_2_); 4.51 (s, 2H, OCH_2_); 7.70 and 8.01 (2s, 2H, NH_2_). ^13^C NMR spectrum (DMSO-d_6_) δ: 10.54; 22.38; 63.66; 72.13; 153.22; 156.84; 159.39. HRMS: for C_7_H_12_N_4_O_2_ *m*/*z* [M + H]^+^ calculated: 185.0960; found: 185.0981; and LC 5-(n-propoxymethyl)-1,2,4-triazole-3-carboxamide content: spectrophotometric detection of 235 nm no less than 98%.

#### 3.1.2. Methyl 1-(Methoxymethyl)-1,2,4-triazole-3-carboxylate (**10a**)

A total of 0.5 g (3.9 mmol) of methyl 1,2,4-triazole-3-carboxylate was suspended in 4 mL (19 mmol) of HMDS and stirred under reflux for 1 h. After cooling, the excess of HMDS was removed using a rotary evaporator. Concentrations of 5 mL of anhydrous acetonitrile, 1.70 mL (18 mmol) of **9a**, 0.45 mL (3.9 mmol) of SnCl_4_ were added to the residue, and the reaction was stirred under reflux until the starting ester was no longer detectable by TLC. The reaction mass was poured into 10 mL of saturated sodium bicarbonate solution and the precipitates formed were filtered off. The filtrate was extracted with chloroform (4 × 10 mL), and the combined chloroform extracts were washed with water (10 mL) and dried over CaCl_2_. The volatile components were evaporated. A total of 0.25 g (38.5%) of the product **10a** was isolated by column chromatography on silica gel, eluent: toluene–acetone, modified with 1% triethylamine (acetone gradient from 5 to 7%), as transparent oil.

R_f_ = 0.26 (30% acetone in toluene). ^1^H NMR spectrum (CDCl_3_) δ: 3.40 (s, 3H, OCH_3_); 4.99 (s, 3H, COOCH_3_); 5.53 (s, 2H, OCH_2_); 8.34 (s, 1H, CH). ^13^C NMR spectrum (CDCl_3_) δ: 52.86; 57.66; 80.39; 145.89; 154.89; 159.93. For C_6_H_9_N_3_O_3_ *m*/*z* [M + H]^+^ calculated: 172.1; found: 172.0.

#### 3.1.3. 1-(Methoxymethyl)-1,2,4-triazole-3-carboxamide (**11a**)

A total concentration of 0.2 g (1.2 mmol) of methyl 1-(methoxymethyl)-1,2,4-triazole-3-carboxylate was dissolved in 1.5 mL of a 10 M ammonia solution in methanol and stirred at room temperature to conversion of the starting material (control by TLC). Volatile components were removed on a rotary evaporator, 0.15 g (87%) of the product **11a** was isolated by recrystallization from a solvent mixture: ethanol–ethyl acetate as white crystals.

R_f_ = 0.53 (1% CH_3_OH in CHCl_3_), mp 146–147 °C. ^1^H NMR spectrum (DMSO-d_6_) δ: 3.29 (s, 3H, OCH_3_); 5.51 (s, 2H, OCH_2_); 7.51 and 7.67 (2s, 2H, NH_2_); 8.80 (s, 1H, CH). ^13^C NMR spectrum (DMSO-d6) δ: 56.58; 79.16; 146.21; 157.48; 160.41. HRMS: for C_5_H_8_N_4_O_2_ *m*/*z* [M + H]^+^ calculated: 157.0726; found: 157.0733; LC 1-(methoxymethyl)-1,2,4-triazole-3-carboxamide content: spectrophotometric detection of 235 nm no less than 98%.

#### 3.1.4. General Procedure for the Preparation of 1-Substituted of 1,2,4-Triazole-3-carboxamides **11b**–**j**, **1c**

Methyl 1,2,4-triazole-3-carboxylate was suspended in 5 eq. HMDS and stirred under reflux for 1 h in an anhydrous atmosphere. After cooling, the excess of HMDS was removed using a rotary evaporator. Anhydrous acetonitrile, 5 eq. **9a**, 1 eq. SnCl_4_ were added to the residue, and the reaction was stirred under reflux until the starting ester was no longer detectable by TLC. The reaction mass was poured into saturated sodium bicarbonate solution, and the precipitates formed were filtered off. The filtrate was extracted with chloroform, the combined chloroform extracts were washed with water (10 mL) and dried over CaCl_2_. The volatile components were evaporated. The product was isolated by column chromatography on silica gel, eluent: toluene–acetone, modified with 1% triethylamine (acetone gradient from 5 to 7%).

1-(Ethoxymethyl)-1,2,4-triazole-3-carboxamide (**11b**).

From 0.5 g (2.5 mmol) of methyl 1,2,4-triazole-3-carboxylate, 0.38 mg (78%) of product **11b** was obtained as white crystals.

R_f_ = 0.52 (1% CH_3_OH in CHCl_3_), mp 127 °C. ^1^H NMR spectrum (DMSO-d_6_) δ: 1.08 (t, *J* = 7.03, 2H, CH_3_CH_2_); 3.29 (s, 3H, OCH_3_); 3.54 (q, *J* = 7.03, 2H, CH_3_CH_2_); 5.55 (s, 2H, OCH_2_); 7.61 and 7.75 (2s, 2H, NH_2_); 8.79 (s, 1H, CH). ^13^C NMR spectrum (DMSO-d_6_) δ: 14.57; 64.50; 77.64; 146.01; 157.37; 160.37. HRMS: for C_6_H_10_N_4_O_2_ *m*/*z* [M + H]^+^ calculated: 171.0882; found: 171.0893; LC 1-(ethoxymethyl)-1,2,4-triazole-3-carboxamide content: spectrophotometric detection of 235 nm no less than 98%.

1-(n-Propyloxymethyl)-1,2,4-triazole-3-carboxamide (**11c**).

From 1 g (7.8 mmol) of methyl 1,2,4-triazole-3-carboxylate, 0.36 mg (78%) of product **11c** was obtained as white crystals.

R_f_ = 0.69 (1% CH_3_OH in CHCl_3_), mp 125–126 °C. ^1^H NMR (DMSO-d_6_) δ: 0.80 (t, 3H, *J* = 7.41, OCH_2_CH_2_CH_3_); 1.41-1.53 (m, 2H, OCH_2_CH_2_CH_3_); 3.44 (t, 2H, *J* = 6.60, OCH_2_CH_2_CH_3_); 5.55 (s, 2H, OCH_2_); 7.57 and 7.79 (2s, 2H, NH_2_); 8.79 (s, 1H, CH). ^13^C NMR (DMSO-d_6_) δ: 10.19; 22.00; 70.61; 77.88; 145.99; 157.35; 160.35. HRMS: for C_5_H_8_N_4_O_2_ *m*/*z* [M + H]^+^ calculated: 185.1038; found: 185.1048. LC 1-(n-propyloxymethyl)-1,2,4-triazole-3-carboxamide content: spectrophotometric detection of 235 nm no less than 97%.

1-(Isopropyloxymethyl)-1,2,4-triazole-3-carboxamide (**11d**).

From 1 g (7.8 mmol) of methyl 1,2,4-triazole-3-carboxylate, 0.42 mg (91%) of product **11d** was obtained as white crystals.

R_f_ = 0.65 (1% CH_3_OH in CHCl_3_), mp 145 °C. ^1^H NMR (DMSO-d_6_) δ: 1.06 (d, 3H, *J* = 6.12, OCHCH_3_); 3.77–3.81 (m, 1H, *J* = 6.11, OCH); 5.56 (s, 2H, OCH_2_); 7.57 and 7.79 (2s, 2H, NH_2_); 8.79 (s, 1H, CH). ^13^C NMR (DMSO-d_6_) δ: 21.94; 70.36; 75.75; 145.98; 157.33; 160.47. HRMS: for C_5_H_8_N_4_O_2_ *m*/*z* [M + H]^+^ calculated: 185.1039; found: 185.1058. LC 1-(isopropyloxymethyl)-1,2,4-triazole-3-carboxamide content: spectrophotometric detection of 235 nm no less than 98%.

1-(n-Butyloxymethyl)-1,2,4-triazole-3-carboxamide (**11e**).

From 1 g (7.8 mmol) of methyl 1,2,4-triazole-3-carboxylate, 0.53 mg (81%) of product **11e** was obtained as white crystals.

R_f_ = 0.5 (1% CH_3_OH in CHCl_3_), mp 123–126 °C. ^1^H NMR spectrum (300 MHz, DMSO-d_6_) δ: 0.80 (t, *J* = 7.41, 2H, CH_3_CH_2_); 1.47 (q, *J* = 6.85, 2H, CH_3_CH_2_); 3.44 (t, *J* = 6.60, 2H, CH_2_CH_2_); 5.55 (s, 2H, OCH_2_); 7.58 and 7.79 (2s, 2H, NH_2_); 8.79 (s, 1H, CH). ^13^C NMR spectrum (75 MHz, DMSO-d_6_) δ: 10.57; 22.00; 70.61; 77.88; 145.99; 157.35; 160.35. HRMS: for C_8_H_14_N_4_O_2_ *m*/*z* [M + H]^+^ calculated: 199.1195; found: 199.1205. LC 1-(n-butyloxymethyl)-1,2,4-triazole-3-carboxamide content: spectrophotometric detection of 235 nm no less than 97%.

1-(tert-Butoxymethyl)-1,2,4-triazole-3-carboxamide (**11f**).

From 1 g (7.8 mmol) of methyl 1,2,4-triazole-3-carboxylate, 0.39 mg (49%) of product **11f** was obtained as white crystals.

R_f_ = 0.65 (1% CH_3_OH in CHCl_3_), mp 194–195 °C. ^1^H NMR (DMSO-d_6_) δ: 1.18 (s, 9H, O(CH_3_)_3_); 5.57 (s, 2H, OCH_2_); 7.55 and 7.75 (2s, 2H, NH_2_); 8.76 (s, 1H, CH). ^13^C NMR (DMSO-d_6_) δ: 27.28; 72.94; 73.51; 132.74; 157.63; 159.84. HRMS: for C_8_H_14_N_4_O_2_ *m*/*z* [M + H]^+^ calculated: 199.1195; found: 199.1208. LC 1-(tert-butoxymethyl)-1,2,4-triazole-3-carboxamide content: spectrophotometric detection of 235 nm no less than 96%.

1-(n-Decyloxymethyl)-1,2,4-triazole-3-carboxamide (**11g**).

From 1 g (7.8 mmol) of methyl 1,2,4-triazole-3-carboxylate, 0.36 mg (78%) of product **11g** was obtained as white crystals.

R_f_ = 0.60 (1% CH_3_OH in CHCl_3_), mp 122–124 °C. ^1^H NMR (DMSO-d_6_) δ: 0.84 (t, 3H, *J* = 6.83, O(CH_2_)_9_CH_3_; 1.20 (s, 14H, OCH_2_CH_2_(CH_2_)_7_CH_3_); 1.42–1.46 (m, 2H, OCH_2_CH_2_(CH_2_)_7_CH_3_); 0.37 (t, 2H, *J* = 6.50, OCH_2_CH_2_(CH_2_)_7_CH_3_); 7.57 and 7.77 (2s, 2H, NH_2_); 8.78 (s, 1H, CH). ^13^C NMR (DMSO-d_6_) δ: 22.00; 28.59; 28.84; 68.97; 145.98; 160.34. HRMS: for C_14_H_26_N_4_O_2_ *m*/*z* [M + H]^+^ calculated: 283.2134; found: 283.2150. LC 1-n-decyloxymethyl-1,2,4-triazole-3-carboxylic acid amide content: spectrophotometric detection 235 nm no less than 96%.

1-(Benzyloxymethyl)-1,2,4-triazole-3-carboxamide (**11h**).

From 1 g (7.8 mmol) of methyl 1,2,4-triazole-3-carboxylate, 0.63 mg (89%) of product **11h** was obtained as white crystals.

R_f_ = 0.65 (1% CH_3_OH in CHCl_3_), mp 168–169 °C. ^1^H NMR (DMSO-d_6_) δ: 4.60 (s, 2H, CH_2_C_6_H_5_); 5.67 (s, 2H, OCH_2_); 7.26–7.37 (m, 2H, C_6_H_5_); 7.60 and 7.82 (2s, 2H, NH_2_); 8.83 (s, 1H, CH). ^13^C NMR (DMSO-d_6_) δ: 70.69; 77.47; 127.65; 128.28; 136.87; 146.19; 157.46; 160.38. HRMS: for C_11_H_12_N_4_O_2_ *m*/*z* [M + H]^+^ calculated: 233.1039; found: 233.1089. LC 1-(benzyloxymethyl)-1,2,4-triazole-3-carboxamide content: spectrophotometric detection of 235 nm no less than 97%.

1-(Cyclopentyloxymethyl)-1,2,4-triazole-3-carboxamide (**11i**).

From 1 g (7.8 mmol) of methyl 1,2,4-triazole-3-carboxylate, 0.31 mg (53%) of product **11i** was obtained as white crystals.

R_f_ = 0.66 (1% CH_3_OH in CHCl_3_), mp 153–154 °C. ^1^H NMR (DMSO-d_6_) δ: 1.46–1.66 (m, 8H, OC_5_H_9_); 4.08 (s, 1H, OCH); 5.54 (s, 2H, OCH_2_); 7.57 and 7.79 (2s, 2H, NH_2_); 8.79 (s, 1H, CH). ^13^C NMR (DMSO-d_6_) δ: 22.90; 31.78; 76.42; 79.97; 145.99; 157.31; 160.40. HRMS: for C_9_H_10_N_4_O_2_ *m*/*z* [M + H]^+^ calculated: 211.1195; found: 211.1208. LC 1-(cyclopentyloxymethyl)-1,2,4-triazole-3-carboxamide content: spectrophotometric detection 235 nm no less than 98%.

1-(Cyclohexyloxymethyl)-1,2,4-triazole-3-carboxamide (**11j**).

From 1 g (7.8 mmol) of methyl 1,2,4-triazole-3-carboxylate, 0.58 mg (82%) of product **11j** was obtained as white crystals.

R_f_ = 0.46 (1% CH_3_OH in CHCl_3_), mp 155–156 °C. ^1^H NMR (DMSO-d_6_) δ: 1.70–1.17 (m, 10H, C_5_H_10_); 3.49–3.51 (m, 1H, OCH); 5.59 (s, 2H, OCH_2_); 7.62 and 7.85 (2s, 2H, NH_2_); 8.81 (s, 1H, CH). ^13^C NMR (DMSO-d_6_) δ: 23.26; 25.06; 31.61; 75.64; 145.97; 157.33; 160.48. HRMS: for C_10_H_16_N_4_O_2_ *m*/*z* [M + H]^+^ calculated: 225.1352; found: 225.1380. LC 1-(cyclohexyloxymethyl)-1,2,4-triazole-3-carboxamide content: spectrophotometric detection of 235 nm no less than 96%.

1-([2-Hydroxyethoxy]methyl)-1,2,4-triazole-3-carboxamide (**1c**).

From 0.2 g (0.99 mmol) of methyl 1,2,4-triazole-3-carboxylate, 0.58 mg (83%) of product **1c** was obtained as white crystals.

R_f_ = 0.35 (5% CH_3_OH in CHCl_3_), mp 154–156 °C. ^1^H NMR spectrum (DMSO-d_6_) δ: 3.44–3.55 (m, 4H, -OCH_2_CH_2_O-); 5.59 (s, 2H, OCH_2_); 7.57 and 7.79 (2s, 2H, NH_2_); 8.79 (s. 1H, CH). ^13^C NMR spectrum (DMSO-d_6_) δ: 59.79; 70.98; 78.09; 146.01; 158.36; 160.39. HRMS: for C_6_H_10_N_4_O_3_ *m*/*z* [M + H]^+^ calculated: 187.0831; found: 187.0838; LC 1-([2-hydroxyethoxy]methyl))-1,2,4-triazole-3-carboxamide content: spectrophotometric detection of 235 nm no less than 97%.

#### 3.1.5. Methyl 1-(Phenoxymethyl)-1,2,4-triazole-3-carboxylate (**10k**)

A total of 2 g (16 mmol) methyl 1,2,4-triazole-3-carboxylate was suspended in 5 mL of a 3.4 M hydrogen chloride solution in 1,4-dioxane and stirred under reflux for 1 h. The excess of 1,4-dioxane was removed using a rotary evaporator. A 3.2 mL (16 mmol) of diphenoxymethane **14k** and 5 mL of 1,4-dioxane were added to the residue, and the reaction was stirred under reflux until the starting ester was no longer detectable by TLC. The volatile components were evaporated. A total of 1.2 g (32%) of the product **10k** was isolated by column chromatography on silica gel, eluent: toluene–acetone, modified with 1% triethylamine (acetone gradient from 5 to 7%), as transparent oil.

R_f_ = 0.62 (30% acetone in toluene). ^1^H NMR spectrum (CDCl_3_) δ: 3.99 (s, 3H, COOCH_3_); 6.46 (s, 2H, OCH_2_); 6.85–7.34 (m, 5H, Ph); 8.07 (s, 1H, CH). ^13^C NMR spectrum (75 MHz, CDCl_3_) δ: 53.33; 81.77; 117.63; 122.93; 130.34; 148.57; 156.56; 157.06; 160.41. For C_6_H_9_N_3_O_3_ *m*/*z* [M + H]^+^ calculated: 234.2; found: 234.1.

#### 3.1.6. 1-(Phenoxymethyl)-1,2,4-triazole-3-carboxamide (**11k**)

This compound was prepared like **11a** from 0.74 g (3.18 mmol) of methyl 1-(phenoxymethyl)-1,2,4-triazole-3-carboxylate **10k** in 2 mL of methanolic ammonia. The yield was 0.31 g (52%) as white crystals.

R_f_ = 0.75 (1% CH_3_OH in CHCl_3_), mp 188–192 °C. ^1^H NMR spectrum (300 MHz, DMSO-d_6_) δ: 6.23 (s, 2H, OCH_2_); 7.03–7.35 (m, 5H, Ph); 7.67 and 7.90 (2s, 2H, NH_2_); 8.79 (s, 1H, CH). ^13^C NMR spectrum (75 MHz, DMSO-d_6_) δ: 75.02; 116.03; 122.63; 129.82; 146.75; 155.74; 160.26. HRMS: for C_10_H_10_N_4_O_2_ *m*/*z* [M + H]^+^ calculated: 219.0882; found: 219.0896; LC 1-(Phenoxymethyl)-1,2,4-triazole-3-carboxamide content: spectrophotometric detection of 235 nm was no less than 95%.

### 3.2. Antiproliferative Assays

#### 3.2.1. Cell Cultures

Acute lymphoblastic leukemia (CCRF-SB) and chronic myeloid leukemia K562 cell lines were obtained from the Bioresource collection of cell lines of N.N. Blokhin National Medical Research Center of Oncology. Cells were cultured in RPMI-1640 media (“Paneco”, Moscow, Russia) supplemented with a 10% fetal bovine serum (“Biowest”, Nuaillé, France), 2 mM L-glutamine, 5 ME/mL penicillin and 5 µg/mL streptomycin (“Paneco”, Russia) at 37 °C and 5% CO_2_.

#### 3.2.2. MTT Assay

Cells were seeded in 96-well plates (15,000 cells/well) and treated with various concentrations (5 nM–1 mM) of ribavirin or its derivatives or a 0.1% solvent DMSO for 24 h. Cell viability was determined using the MTT assay. Cells were incubated at 37 °C for 3 h with a solution of 3-(4,5-dimethylthiazol-2-yl)-2,5-diphenyltetrazolium bromide (“Paneco”, Russia) in PBS, final concentration 0.25 mg/mL in well. The supernatant was discarded, and the formazan was dissolved in 100 μL of DMSO. The absorbance values were measured at 570 nm on a Microplate Photometer Multiskan FC (“Thermo Fisher Scientific”, Waltham, MA, USA). The percentage of viable cells was calculated as a percentage of solvent-treated control. Each concentration was tested in three technical and three biological replicates.

#### 3.2.3. Cell Proliferation

Cells were seeded in 24-well plates (30,000 cells/well), treated with ribavirin or its derivatives or a 0.1% solvent (DMSO) and incubated for 24 h 48 or 72 h. Cytarabine (Cyt, “SelleckChem”, Houston, TX, USA) was used as a positive control (at 10 nM). Then, cells were stained with 0.4% trypan blue in a PBS (pH 7.4) solution (1:1 *v*/*v*) and immediately counted using a TC20 automatic cell counter (“Bio-Rad”, Hercules, CA, USA). Each point was tested in two technical and three biological replicates.

#### 3.2.4. Cell Cycle

Cells were cultured in 24-well plates (30,000 cells/well) and treated with a 0.1% DMSO (solvent control), 10 nM Cyt (positive control), ribavirin or its derivatives for 72 h. Then, cells were fixed in 70% ethanol for 2 h at 4 °C. Cells were then washed twice with cold PBS, pH 7.4, then stained with a 500 µL cold propidium iodide (PI) solution (50 µg/mL PI, 1% Triton X-100 and 100 µg/mL RNase A in PBS). The cell cycle distribution of cells in samples were analyzed using a FACSCalibur Flow Cytometer (“BD Biosciences”, San Jose, CA, USA). Each point was tested in two technical and three biological replicates.

#### 3.2.5. Human Peripheral Blood Mononuclear Cell (PBMC) Isolation and Culture

Peripheral blood samples were collected from 3 healthy volunteers (21–28-years-old, non-smoking). Monocytes were isolated by centrifugation with Ficoll-Isopaque (“Paneco”, Russia) and then cultured in the RPMI-1640 media (“Paneco”, Russia) supplemented with 20% FBS (“Biowest”, France), 2 mM L-glutamine, 0.5 ME/mL penicillin and 0.5 µg/mL streptomycin (“Paneco”, Russia), 10 mg/L phytohaemagglutinin (“Paneco”, Russia). Cells were incubated with a 0.1% DMSO (solvent control), 10 nM Cyt or 500 µM of **1a** or its derivatives for 72 h at 37 °C and 5% CO_2_. Each point was tested in three technical replicates.

### 3.3. Antimicrobial Assays

Antimicrobial activity was determined with the standard method of agar wells measuring the diameter of the inhibition zones. The following microorganisms from the collection of cultures of the Gause Institute of New Antibiotics were used as test cultures: *Staphylococcus aureus* INA 00985, *Micrococcus luteus* ATCC 9341, *Pseudomonas aeruginosa* ATCC 27853, *Candida albicans* ATCC 14053. The cultures were grown at 35 °C on the following media: Mueller–Hinton agar (*Staphylococcus aureus* INA 00985, *Micrococcus luteus* ATCC 9341, *Pseudomonas aeruginosa* ATCC 27853) and Sabouraud agar (*Candida albicans* ATCC 14053) for 24 h before assay preparation. Preparation of inoculum: the cell density of the bacterial suspension in sterile saline was 0.5 McFarland standard, completely suspended by shaking on a vortex mixer for 10–15 s and applied to Petri dishes with Mueller–Hinton agar and Mueller–Hinton agar with 2% glucose for *Candida albicans*. Plates were incubated at 35 °C. Growth inhibition zone sizes were measured after 24 h of incubation.

### 3.4. Statistical Analysis

All data were calculated as the mean ± standard error of mean (S.E.M.). The data were analyzed using GraphPad v8.2.1 software (San Diego, CA, USA). The treatment effects in each experiment were compared by one-way Student’s *t*-test. Differences between groups were considered significant at *p* < 0.05. All in vitro experiments were repeated three times in 2–3 technical replications.

### 3.5. In Silico Studies

The crystal structure of eIF4E was obtained from the Protein Data Bank (PDB ID: 4TPW) as a co-complex of the translation initiation factor eIF4E with the inhibitor 4EGI-1. The selected structure of the complex has a resolution of 1.5 Å and does not contain gaps in the protein backbone near the ligand binding domain. Schrödinger Suite 2020 software (Schrödinger, Inc., New York, NY, USA; Maestro Version 12.5.139, MMshare Version 5.1.139, Release 2020-3) was used to perform the modelling. The removal of inhibitor and solvent molecules, addition of hydrogen atoms, assignment of atom types, combining of non-polar hydrogen atoms, and calculation of Gasteiger partial charges and Kollman charges were performed using Schrödinger Suite 2020 software (Schrödinger, Inc., USA) with the Schrödinger Maestro Protein Preparation Wizard module. During the docking process, all torsional bonds of the ligands were free to rotate, while the protein remained rigid. The visualization and graphical representation of ligand interaction results were performed using Schrödinger Maestro software.

## 4. Conclusions

In the present work, we synthesized two series of fully deoxy acyclic analogues of ribavirin—5-alkyl/aryloxymethyl **6** and 1-alkyl/aryloxymethyl **11** derivatives of 1,2,4-triazole-3-carboxamide, and compared their anticancer and antimicrobial properties. Derivatives of series **6** apparently lose even the weak antimicrobial potential characteristic of ribavirin **1a**, while 1-alkyl/aryloxymethyls of series **11** show antimicrobial activity against Gram-positive bacteria.

Novel derivatives of 1,2,4-triazole-3-carboxamide **6g** and **11g** exhibited high cytostatic effects and antiproliferative activities in leukemia cell lines. The effect of the new compounds was comparable to ribavirin or Cyt (in the K562 line) and revealed specific cytotoxicity to leukemia cells compared to PBMC. Thus, it was shown that compounds with n-decyloxymethyl radicals, regardless of the triazole ring substitution position, exhibit anticancer activity. Molecular docking results suggest that cell cycle arrest and the suppression of cell proliferation may be mediated by the inhibition of eIF4E, like in the case of ribavirin.

These results imply that alkyloxymethyl-1,2,4-triazole-3-carboxamides have potential for further development and applications as anticancer agents. Due to the significant structural, and therefore functional, differences between alkyloxymethyl-1,2,4-triazole-3-carboxamides and nucleoside analogues, it can be assumed that they will have fewer therapeutic side effects.

## Figures and Tables

**Table 1:** Synthesized 5-alkyl/aryloxymethyl-1,2,4-triazole-3-carboxamides **6**.

	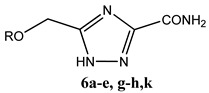	
S. No.	R	Yield
**6a**	Me	60%
**6b**	Et	53%
**6c**	n-Pr	62%
**6d**	i-Pr	24%
**6e**	n-Bu	33%
**6g**	n-C_10_H_21_	43%
**6h**	Bn	68%
**6k**	Ph	76%

**Table 2:** Synthesized 1-alkyl/aryloxymethyl-1,2,4-triazole-3-carboxamides **11**.

	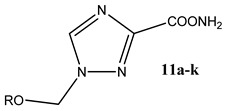	
S. No.	R	Yield
**11a**	Me	87%
**11b**	Et	78%
**11c**	n-Pr	78%
**11d**	i-Pr	91%
**11e**	n-Bu	81%
**11f**	t-Bu	49%
**11g**	n-C_10_H_21_	78%
**11h**	Bn	89%
**11i**	cyclopentyl	82%
**11j**	cyclohexyl	53%
**11k**	Ph	52%
**1c**	HO(CH_2_)_2_	83%

**Table 3:** CC_50_ values for compounds after incubation with cells for 24 and 72 h (MTT assay, *n* = 3).

5-Alkyl/aryloxymethyl-1,2,4-triazole-3-carboxamides					1-Alkyl/aryloxymethyl-1,2,4-triazole-3-carboxamides				
CC_50_, µM					CC_50_, µM				
S. No.	K562		CCRF-SB		S. No.	K562		CCRF-SB	
	24 h	72 h	24 h	72 h		24 h	72 h	24 h	72 h
				**1a (ribavirin)**		270 ± 11	10 ± 1	-	188 ± 31
**2g**	260 ± 12 *		240 ± 16 *		**1g**	240 ± 22 *		230 ± 18 *	
**2h**	240 ± 21 *		250 ± 13 *		**1h**	230 ± 13 *		270 ± 25 *	
**6a**	-	-	-	-	**11a**	-	-	-	-
**6b**	-	-	-	-	**11b**	-	-	-	-
**6c**	-	-	-	-	**11c**	-	-	-	-
**6d**	-	-	-	-	**11d**	-	-	-	-
**6e**	-	-	-	-	**11e**	-	-	-	-
					**11f**	-	-	-	-
**6g**	391 ± 15	43 ± 7	500 ± 100	-	**11g**	14 ± 0	13 ± 3	112 ± 19	62 ± 2
**6h**	-	-	-	-	**11h**	-	-	-	-
					**11i**	-	-	-	-
					**11j**	-	-	-	-
**6k**	-	-	-	-	**11k**	-	-	-	-
					**1c**	-	-	-	-
				**Cyt**		59.4 ± 14.0	58.1 ± 16.9	15.8 ± 4.1	0.1 ± 0.1

“-”—not active. * Data were previously obtained and published in [9].

**Table 4:** 1-Alkyl/aryloxymethyl-1,2,4-triazole-3-carboxamides antimicrobial effects.

S. No.	Zone of Growth Inhibition, mm			
	*S. aureus*	*M. luteus*	*P. aeruginosa*	*C. albicans*
**1-Alkyl/aryloxymethyl-1,2,4-triazole-3-carboxamides**				
**1a**	-	-	25 ± 1	30 ± 1
**11a**	-	-	-	-
**11b**	-	-	-	-
**11c**	-	-	12 ± 1	-
**11d**	-	-	-	-
**11e**	-	-	-	-
**11f**	-	-	-	-
**11g**	-	-	-	-
**11h**	-	-	-	-
**11i**	-	12 ± 1	-	-
**11j**	-	12 ± 1	-	-
**11k**	-	-	-	-
**1c**	-	12 ± 1	-	-

“-”—not active.

## Data Availability

Data are contained within the article and Supplementary Materials.

## Supplementary Materials

The following supporting information can be downloaded at: https://www.mdpi.com/article/10.3390/molecules29204808/s1. Refs. [38,39] are cited in Supplementary Materials.

## References

[1] Chudinov M.V. (2019). Ribavirin and its analogues: Can you teach an old dog new tricks?. Fine Chem. Technol..

[2] Gonzalez S. (2022). Anti-HCV and Zika activities of ribavirin C-nucleosides analogues. Bioorg. Med. Chem..

[3] Sardushkin M.V., Shiryaeva Y.K., Donskaya L., Vifor R., Donina M.V. (2020). Colloid-Chemical and Antimicrobial Properties of Ribavirin Aqueous Solutions. Sys. Rev. Pharm..

[4] Assouline S., Culjkovic-Kraljacic B., Bergeron J., Caplan S., Cocolakis E., Lambert C., Borden K.L.B. (2014). A phase I trial of ribavirin and low-dose cytarabine for the treatment of relapsed and refractory acute myeloid leukemia with elevated eIF4E. Haematologica.

[5] Way H., Roh J., Venteicher B., Chandra S., Thomas A.A. (2020). Synthesis of ribavirin 1,2,3-and 1,2,4-triazolyl analogs with changes at the amide and cytotoxicity in breast cancer cell lines. Nucleosides Nucleotides Nucleic Acids.

[6] Bózsity N., Minorics R., Szabó J., Mernyák E., Schneider G., Wölfling J., Wang H.C., Wuc C.C., Ocsovszki I., Zupkó I. (2017). Mechanism of antiproliferative action of a new d-secoestrone-triazole derivative in cervical cancer cells and its effect on cancer cell motility. J. Steroid Biochem. Mol. Biol..

[7] Szabó J., Bacsa I., Wölfling J., Schneider G., Zupkó I., Varga M., Hermab B.E., Kalmar L., Szecsi M., Mernyák E. (2015). Synthesis and in vitro pharmacological evaluation of N-[(1-benzyl-1,2,3-triazol-4-yl)methyl]-carboxamides on d-secoestrone scaffolds. J. Enzym. Inhib..

[8] Ilovaisky A.I., Scherbakov A.M., Chernoburova E.A., Povarov A.A., Shchetinina M.A., Merkulova V.M., Salnikova D.I., Sorokin D.V., Bozhenko E.I., Zavarzin I.V. (2023). Secosteroid thiosemicarbazides and secosteroid–1,2,4-triazoles as antiproliferative agents targeting breast cancer cells: Synthesis and biological evaluation. J. Steroid Biochem. Mol. Biol..

[9] Zhidkova E., Stepanycheva D., Grebenkina L., Mikhina E., Maksimova V., Grigoreva D., Matveev A., Lesovaya E. (2023). Synthetic 1,2,4-triazole-3-carboxamides Induce Cell Cycle Arrest and Apoptosis in Leukemia Cells. Curr. Pharm. Des..

[10] Wittine K., Stipković B.M., Makuc D., Plavec J., Kraljević P.S., Sedić M., Pavelić K., Leyssen P., Neyts J., Balzarini J. (2012). Novel 1,2,4-triazole and imidazole derivatives of L-ascorbic and imino-ascorbic acid: Synthesis, anti-HCV and antitumor activity evaluations. Bioorg. Med. Chem..

[11] Shen G.Y., Robins R.K., Revankar G.R. (1991). Synthetic Studies on the Isomeric N-Methyl Derivatives of C-Ribavirin. Nucleosides Nucleotides.

[12] Wan J.Q., Xia Y., Liu Y., Wang M.H., Rocchi P., Yao J.H., Qu F.Q., Neyts J., Iovanna J.L., Peng L. (2009). Discovery of novel arylethynyltriazole ribonucleosides with selective and effective antiviral and antiproliferative activity. J. Med. Chem..

[13] Zhu R.Z., Wang M.H., Xia Y., Qu F.Q., Neytsc J., Peng L. (2008). Arylethynyltriazole acyclonucleosides inhibit hepatitis C virus replication. Bioorg. Med. Chem. Lett..

[14] Xia L., Chunxian L., Lianjia Z. (2021). Advance of structural modification of 13 nucleosides scaffold. Eur. J. Med. Chem..

[15] Grebenkina L.E., Prutkov A.N., Matveev A.V., Chudinov M.V. (2022). Synthesis of 5-oxymethyl-1,2,4-triazole-3-carboxamides. Fine Chem. Technol..

[16] Tsilevich T.L., Shchaveleva I.L., Nosach L.N., Zhovnovataia V.L., Smirnov I.P. (1988). Acyclic analogues of ribavirine. Synthesis and antiviral activity. Bioorg. Chem..

[17] Tsilevich T.L., Zavgorodniy S.G., Marks U., Ionova L.V., Florentev V.L. (1986). Synthesis of ribavirin acyclic analogues. Bioorg. Chem..

[18] Hughes W.B., Kleene R.D. (1954). Reaction of methylal with some acid anhydrides. Serv. Res. Dev. Co..

[19] Thenet K., Beydoun K., Wiesenthal J., Leitner W., Klankermayer J. (2016). Ruthenium-catalyzed synthesis of dialkoxymethane ethers utilizing carbon dioxide and molecular hydrogen. Angew. Chem. Int. Ed..

[20] Matsumoto H., Kaneko C., Yamada K., Takeuchi T., Mori T., Mizuno Y. (1988). A Convenient Synthesis of 9-(2-Hydroxyethoxymethyl)guanine (Acyclovir) and Related Compounds. Chem. Pharm. Bull..

[21] Wenming L., Szewczy J., Liladhar W. (2003). Practical synthesis of diaryloxymethanes. J. Synth. Org. Chem. Jpn..

[22] Cruz-Hernandez E., Medina-Franco J., Trujillo J., Chavez-Blanco A., Dominguez-Gomez G., Perez-Cardenas E., Gonzalez-Fierro A., Taja-Chayeb L., Dueïas-Gonzalez A. (2015). Ribavirin as a tri-targeted antitumor repositioned drug. Oncol. Rep..

[23] Hedstrom L. (2009). IMP Dehydrogenase: Structure, mechanism, and inhibition. Chem. Rev..

[24] Konno Y., Natsumeda Y., Nagai M., Yamaji Y., Ohno S., Suzuki K., Weber G. (1991). Expression of human IMP dehydrogenase types I and II in Escherichia coli and distribution in human normal lymphocytes and leukemic cell lines. J. Biol. Chem..

[25] Hongxia T., Li H., Zhenshun C. (2020). Inhibition of eIF4E signaling by ribavirin selectively targets lung cancer and angiogenesis. Biochem. Biophys. Res. Commun..

[26] Nagai M., Natsumeda Y., Konno Y., Hoffman R., Irino S., Weber G. (1991). Selective up-regulation of type II inosine 5′-monophosphate dehydrogenase messenger RNA ex-pression in human leukemias. Cancer Res..

[27] Urtishak K.A., Wang L.S., Culjkovic-Kraljacic B., Davenport J.W., Porazzi P., Vincent T.L., Teachey D.T., Tasian S.K., Moore J.S., Seif A.E. (2019). Targeting EIF4E signaling with ribavirin in infant acute lymphoblastic leukemia. Oncogene.

[28] Volpin F., Casaos J., Sesen J., Mangraviti A., Choi J., Gorelick N., Frikeche J., Lott T., Felder R., Scotland S.J. (2017). Use of an anti-viral drug, Ribavirin, as an anti-glioblastoma therapeutic. Oncogene.

[29] Kraljaci B.C., Arguello M., Amri A., Cormack G., Borden K. (2011). Inhibition of eIF4E with ribavirin cooperates with common chemotherapies in primary acute myeloid leukemia specimens. Leukemia.

[30] Kentsis A., Volpon L., Topisirovic I., Soll C.E., Culjkovic B., Shao L., Borden K.L. (2005). Further evidence that ribavirin interacts with eIF4E. RNA.

[31] Silvera D., Formenti S.C., Schneider R.J. (2010). Translational control in cancer. Nat. Rev. Cancer..

[32] Papadopoulos E., Jenni S., Kabha E., Wagner G. (2014). Structure of the eukaryotic translation initiation factor eIF4E in complex with 4EGI-1 reveals an allosteric mechanism for dissociating eIF4G. Proc. Natl. Acad. Sci. USA.

[33] Fan S., Li Y., Yue P., Khuri F.R., Sun S.Y. (2010). The eIF4E/eIF4G interaction inhibitor 4EGI-1 augments TRAIL-mediated apoptosis through c-FLIP down-regulation and DR5 induction independent of inhibition of cap-dependent protein translation. Neoplasia.

[34] Moerke N.J., Aktas H., Chen H., Cantel S., Reibarkh M.Y., Fahmy A., Gross J.D., Degterev A., Yuan J., Chorev M. (2007). Small-molecule inhibition of the interaction between the translation initiation factors eIF4E and eIF4G. Cell.

[35] Chen L., Aktas B.H., Wang Y., He X., Sahoo R., Zhang N., Denoyelle S., Kabha E., Yang H., Freedman R.Y. (2012). Tumor suppression by small molecule inhibitors of translation initiation. Oncotarget.

[36] Tamburini J., Green A.S., Bardet V., Chapuis N., Park S., Willems L., Uzunov M., Ifrah N., Dreyfus F., Lacombe C. (2009). Protein synthesis is resistant to rapamycin and constitutes a promising therapeutic target in acute myeloid leukemia. Blood.

[37] Descamps G., Gomez-Bougie P., Tamburini J., Green A., Bouscary D., Maiga S., Moreau P., Gouill S.L., Pellat-Deceunynck C., Amiot M. (2012). The cap-translation inhibitor 4EGI-1 induces apoptosis in multiple myeloma through Noxa induction. Br. J. Cancer..

[38] Fieser L., Fieser M. (1967). Reagents for Organic Synthesis.

[39] Gottlieb H.E., Kotlyar V., Nudelman A. (1997). NMR Chemical Shifts of Common Laboratory Solvents as Trace Impurities. J. Org. Chem..

