# Supplementary material for: Synthesis of Alkyl/Aryloxymethyl Derivatives of 1,2,4-Triazole-3-Carboxamides and Their Biological Activities
Source: Molecules. 2024 Oct 11;29(20):4808. doi: 10.3390/molecules29204808 (PMC11509950; doi:10.3390/molecules29204808)
Supplement: Supplementary file 1 [file molecules-29-04808-s001.zip › molecules-3237551-supplementary.pdf]

Supporting Information of

«Synthesis of Alkyl/Aryloxymethyl Derivatives of 1,2,4-Triazole-3-Carboxamides and Their Biological Activities»

Ekaterina A. Mikhina [1], Daria V. Stepanycheva [2], Varvara P. Maksimova [2], Olga N. Sineva [3], Natalia N. Markelova [3], Lyubov E. Grebenkina [1], Ekaterina A. Lesovaya [2,4,5], Marianna G. Yakubovskaya [2], Andrey V. Matveev [1] and Ekaterina M. Zhidkova [2,*]

1 Lomonosov Institute of Fine Chemical Technologies, MIREA-Russian Technological University, 86 Vernadsky Prospekt, Moscow 119571, Russia; mik.hi@mail.ru (E.A.M.); legrebenkina@mail.ru (L.E.G.); 4motya@gmail.com (A.V.M.)
2 Department of Chemical Carcinogenesis, N.N. Blokhin Russian Cancer Research Center, Ministry of Health of Russia, 24 Kashirskoe Shosse, Moscow 115478, Russia; darya.stepanycheva@yandex.ru (D.V.S.); lavarvar@gmail.com (V.P.M.); lesovenok@yandex.ru (E.A.L.); mgyakubovskaya@mail.ru (M.G.Y.)
3 Gause Institute of New Antibiotics, 11 Bolshaya Pirogovskaya St., Moscow 119021, Russia; olga.sineva81@yandex.ru (O.N.S.); nathanmrk82@gmail.com (N.N.M.)
4 Faculty of Oncology, I.P. Pavlov Ryazan State Medical University, Ministry of Health of Russia, 9 Vysokovol'tnaya St., Ryazan 390026, Russia
5 Laboratory of Single Cell Biology, Friendship University of Russia, 6 Miklukho-Maklaya St., Moscow 117198, Russia
* Correspondence: e.zhidkova@ronc.ru

Table of contents

Instrument and methods 3
Experimental section 4
Copies of NMR and MS spectra: 11
***In Silico* Studies** 50

Instrument and methods

All the chemical reagents were obtained from commercial suppliers and used without further purification. All the solvents used in the reactions were dried accord-ing to the described methods [38] or were distilled before use. Reactions were monitored by thin layer chromatography (TLC) performed on PTX-AF-A-UV silica gel plates (Sorbfil, Russia). The visualization of spots on the chromatograms was carried out using UV irradiation at 254 nm, as well as iodine vapor and phosphomolybdic acid. Column chromatography was carried on silica gel Kieselgel 60 (Merck, Germany). Solvents were removed on a Buchi Rotavapor R-200 rotary vacuum evaporator using a vacuum water jet pump with a vacuum (20 mm Hg) (Büchi, Switzerland).

^{1}H and ^{13}C NMR spectra were recorded on a Bruker DPX-300 (Bruker, Germany) instrument (300 and 75 MHz, respectively). Internal standard – residual solvent signals ($CDCl_3$: 7.25 ppm for ^{1}H, 77.2 ppm for ^{13}C; DMSO-d_6: 2.49 ppm for ^{1}H, 39.5 ppm for ^{13}C). The data are presented as follows: chemical shifts, multiplicity, coupling constants J (Hz) and the relative value of integration. Designations in ^{1}H-NMR spectra: s – singlet, d – doublet, t – triplet, q – quartet, m – multiplet, se-sextet, sp – septet. The determination of solvent peaks was carried out in accordance with the literature data [39]. Two-dimensional NMR spectra were recorded on the Bruker 400 MHz WB Avance II (Bruker, Germany) instrument with a resonant frequency for ^{1}H = 400 MHz, ^{13}C = 100 MHz. LCMS were performed on a Shimadzu LCMS-2020 (Shimadzu, Japan) device in gradient mode with electrospray ionization. High-resolution mass spectra (HRMS) were recorded on Agilent 6224 (Agilent Technologies, USA, California) using electron spray ionization (ESI).Melting temperatures are determined on the MP-200D-120 (Cole-Parmer, UK) device in an open capillary.

Experimental section

5-(n-Propoxymethyl)-1,2,4-triazole-3-carboxamide (6c)

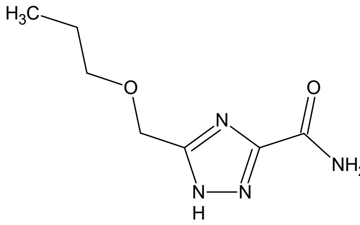

R_f = 0.61 (1% CH_3OH in $CHCl_3$), **mp** 110-111°C. **^{1}H NMR spectrum (DMSO-d_6) δ:** 0.83 (t, 3H, J=7.09, $\underline{CH_3}CH_2CH_2$); 1.50 (se, 2H, J=7.09, $CH_3\underline{CH_2}CH_2$); 3.40 (t, 2H, J=6.09, $CH_3CH_2\underline{CH_2}$); 4.51 (s, 2H, OCH_2); 7.70 and 8.01 (2s, 2H, NH_2). **^{13}C NMR spectrum (DMSO-d_6) δ:** 10.54; 22.38; 63.66; 72.13; 153.22; 156.84; 159.39. **HRMS:** for $C_7H_{12}N_4O_2$ m/z $[M+H]^+$ calculated: 185.0960; found: 185.0981; **LC** 5-(*n*-propoxymethyl)-1,2,4-triazole-3-carboxamide content: spectrophotometric detection of 235 nm no less than 96%.

Methyl 1-(Methoxymethyl)-1,2,4-triazole-3-carboxylate acid (10a)

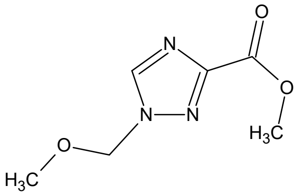

R_f = 0.26 (30% acetone in toluene). **^{1}H NMR spectrum ($CDCl_3$) δ**: 3.40 (s, 3H, OCH_3); 4.99 (s, 3H, $COOCH_3$); 5.53 (s, 2H, OCH_2); 8.34 (s, 1H, CH). **^{13}C NMR spectrum ($CDCl_3$) δ:** 52,86; 57.66; 80.39; 145.89; 154.89; 159.93.

1-(Methoxymethyl)-1,2,4-triazole-3-carboxamide (11a)

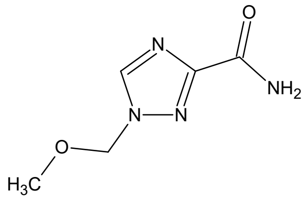

R_f = 0.53 (1% CH_3OH in chloroform), **mp** 151-152°C. **^{1}H NMR spectrum ($CDCl_3$) δ:** 3.29 (s, 3H, OCH_3); 5.51 (s, 2H, OCH_2); 7.51 and 7.67 (2s, 2H, NH_2); 8.80 (s, 1H, CH). **^{13}C NMR spectrum ($CDCl_3$) δ:** 56.58; 79.16; 146.21; 157.48; 160.41. **HRMS:** for $C_5H_8N_4O_2$ m/z $[M+H]^+$ calculated: 157.0726; found:

157.0733; **LC** 1-(methoxymethyl)-1,2,4-triazole-3-carboxamide content: spectrophotometric detection of 235 nm no less than 98%.

1-(Ethoxymethyl)-1,2,4-triazole-3-carboxamide (11b)

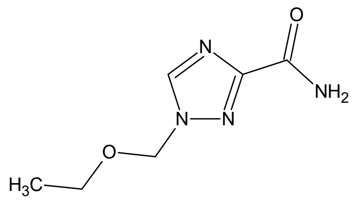

R_f = 0.52 (1% CH_3OH in chloroform), **mp** 127°C. **^{1}H NMR spectrum (DMSO-d_6) δ:** 1.08 (t, J=7.0, 3H, $\underline{CH_3}CH_2$); 3.29 (s, 3H, OCH_3); 3.54 (q, J=7.03, 2H, $CH_3\underline{CH_2}$); 5.55 (s, 2H, OCH_2); 7.61 and 7.75 (2s, 2H, NH_2); 8.79 (s, 1H, CH). **^{13}C NMR spectrum (DMSO-d_6) δ:** 14.57; 64.50; 77.64; 146.01; 157.37; 160.37. **HRMS:** for $C_6H_{10}N_4O_2$ m/z $[M+H]^+$ calculated: 171.0882; found: 171.0893; **LC** 1-(ethoxymethyl)-1,2,4-triazole-3-carboxamide content: spectrophotometric detection of 235 nm no less than 98%.

1-(n-Propoxymethyl)-1,2,4-triazole-3-carboxamide (11c)

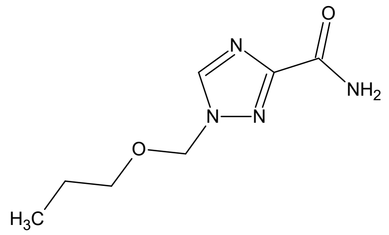

R_f = 0.69 (1% CH_3OH in chloroform), **mp** 125-126°C. **^{1}H NMR spectrum (DMSO-d_6) δ:** 0.80 (t, 3H, J = 7.41, $OCH_2CH_2\underline{CH_3}$); 1.41-1.53 (m, 2H, $OCH_2\underline{CH_2}CH_3$); 3.44 (t, 2H, J = 6.60, $O\underline{CH_2}CH_2CH_3$); 5.55 (s, 2H, OCH_2); 7.57 and 7.79 (2s, 2H, NH_2); 8.79 (s, 1H, CH). **^{13}C NMR spectrum (DMSO-d_6) δ:** 10.19; 22.00; 70.61; 77.88; 145.99; 157.35; 160.35. **HRMS:** for $C_5H_8N_4O_2$ m/z $[M+H]^+$ calculated: 185.1038; found: 185.1048. **LC** 1-(n-propyloxymethyl)-1,2,4-triazole-3-carboxamide content: spectrophotometric detection of 235 nm no less than 97%.

1-(Isopropoxymethyl)-1,2,4-triazole-3-carboxamide (11d)

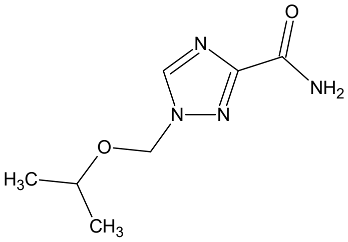

R$_f$ = 0.65 (1% CH_3OH in chloroform), **mp** 145°C. **^{1}H NMR spectrum (DMSO-d$_6$) δ:** 1.06 (d, 6H, J = 6.12, OCH(CH_3)$_2$); 3.77-3.81 (m, 1H, J = 6.11, OCH); 5.56 (s, 2H, OCH_2); 7.57 and 7.79 (2s, 2H, NH_2); 8.79 (s, 1H, CH). **^{13}C NMR spectrum (DMSO-d$_6$) δ:** 21.94; 70.36; 75.75; 145.98; 157.33; 160.47. **HRMS:** for $C_5H_8N_4O_2$ m/z $[M+H]^+$ calculated: 185.1039; found: 185.1058. **LC** 1-(isopropyloxymethyl)-1,2,4-triazole-3-carboxamide content: spectrophotometric detection of 235 nm no less than 98%.

1-(n-Butyloxymethyl)-1,2,4-triazole-3-carboxamide (11e)

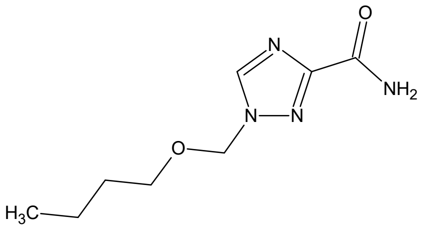

R$_f$ = 0.50 (1% CH_3OH in chloroform), **mp** 123-126°C. **^{1}H NMR spectrum (DMSO-d$_6$) δ:** 0.80 (t, J=7.41, 3H, CH_3CH_2); 1.47 (q, J=6.85 Hz, 2H, CH_3CH_2); 3.44 (t, J=6.60, 4H, CH_2CH_2); 5.55 (s, 2H, OCH_2); 7.58 and 7.79 (2s, 2H, NH_2); 8.79 (s, 1H, CH). **^{13}C NMR spectrum (DMSO-d$_6$) δ:** 10.57; 22.00; 70.61; 77.88; 145.99; 157.35; 160.35. **HRMS:** for $C_8H_{14}N_4O_2$ m/z $[M+H]^+$ calculated: 199.1195; found: 199.1205. **LC** 1-(n-butyloxymethyl)-1,2,4-triazole-3-carboxamide content: spectrophotometric detection of 235 nm no less than 97%.

1-(tert-Butoxymethyl)-1,2,4-triazole-3-carboxamide (11f)

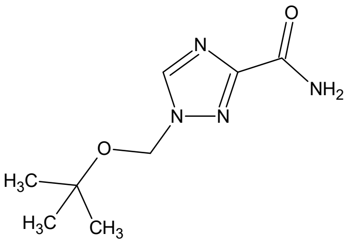

R_f = 0.65 (1% CH_3OH in chloroform), **mp** 194-195°C. **^{1}H NMR spectrum (DMSO-d_6) δ:** 1.18 (s, 9H, O$\underline{(CH_3)_3}$); 5.57 (s, 2H, OCH_2); 7.55 and 7.75 (2s, 2H, NH_2); 8.76 (s, 1H, CH). **^{13}C NMR spectrum (DMSO-d_6) δ:** 27.28; 72.94; 73.51; 132.74; 157.63; 159.84. **HRMS:** for $C_8H_{14}N_4O_2$ m/z $[M+H]^+$ calculated: 199.1195; found: 199.1208. **LC** 1-(*tert*-butoxymethyl)-1,2,4-triazole-3-carboxamide content: spectrophotometric detection of 235 nm no less than 96%.

1-n-Decyloxymethyl-1,2,4-triazole-3-carboxylic acid amide (11g)

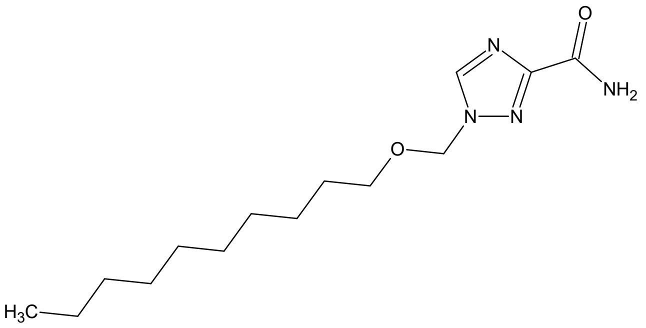

R_f = 0.60 (1% CH_3OH in chloroform), **mp** 122-124°C. **^{1}H NMR spectrum (DMSO-d_6) δ:** 0.84 (t, 3H, J = 6.83, $O(CH_2)_9\underline{CH_3}$; 1.20 (s, 14H, $OCH_2CH_2\underline{(CH_2)_7}CH_3$); 1.42-1.46 (m, 2H, $OCH_2\underline{CH_2}(CH_2)_7CH_3$); 3.37 (t, 2H, J = 6.50, $O\underline{CH_2}CH_2(CH_2)_7CH_3$); 7.57 and 7.77 (2s, 2H, NH_2); 8.78 (s, 1H, CH). **^{13}C NMR spectrum (DMSO-d_6) δ:** 22.00; 28.59; 28.84; 68.97; 145.98; 160.34. **HRMS:** for $C_{14}H_{26}N_4O_2$ m/z $[M+H]^+$ calculated: 283.2134; found: 283.2150. **LC** 1-(*n*-decyloxymethyl)-1,2,4-triazole-3-carboxamide content: spectrophotometric detection of 235 nm no less than 96%.

1-(Benzyloxymethyl)-1,2,4-triazole-3-carboxamide (11h)

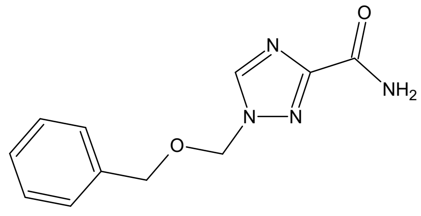

R_f = 0.65 (1% CH_3OH in chloroform), **mp** 168-169°C. **^{1}H NMR spectrum (DMSO-d$_6$) δ:** 4.60 (s, 2H, $CH_2C_6H_5$); 5.67 (s, 2H, OCH_2); 7.26-7.37 (m, 2H, C6H5); 7.60 and 7.82 (2s, 2H, NH_2); 8.83 (s, 1H, CH). **^{13}C NMR spectrum (DMSO-d$_6$) δ:** 70.69; 77.47; 127.65; 128.28; 136.87; 146.19; 157.46; 160.38. **HRMS:** for $C_{11}H_{12}N_4O_2$ m/z $[M+H]^+$ calculated: 233.1039; found: 233.1089. **LC** 1-(benzyloxymethyl)-1,2,4-triazole-3-carboxamide content: spectrophotometric detection of 235 nm no less than 97%.

1-(Cyclopentyloxymethyl)-1,2,4-triazole-3-carboxamide (11i)

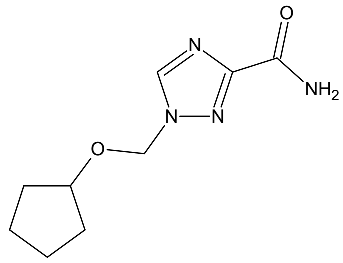

R_f = 0.66 (1% CH_3OH in chloroform), **mp** 153-154°C. **^{1}H NMR spectrum (DMSO-d$_6$) δ:** 1.46-1.66 (m, 8H, OC_5H_9); 4.08 (s, 1H, OCH); 5.54 (s, 2H, OCH_2); 7.57 and 7.79 (2s, 2H, NH_2); 8.79 (s, 1H, CH). **^{13}C NMR spectrum (DMSO-d$_6$) δ:** 22.90; 31.78; 76.42; 79.97; 145.99; 157.31; 160.40**. HRMS:** for $C_9H_{10}N_4O_2$ m/z $[M+H]^+$ calculated: 211.1195; found: 211.1208. **LC** 1-(cyclopentyloxymethyl)-1,2,4-triazole-3-carboxamide content: spectrophotometric detection of 235 nm no less than 98%.

1-(Cyclohexyloxymethyl)-1,2,4-triazole-3-carboxamide (11j)

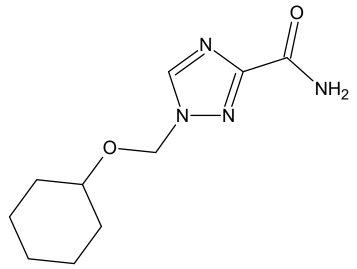

R$_f$ = 0.46 (1% CH_3OH in chloroform), **mp** 155-156°C. **^{1}H NMR spectrum (DMSO-d$_6$) δ:** 1.70-1.17 (m, 10H, C_5H_{10}); 3.49-3.51 (m, 1H, OCH); 5.59 (s, 2H, OCH_2); 7.62 and 7.85 (2s, 2H, NH_2); 8.81 (s, 1H, CH). **^{13}C NMR spectrum (DMSO-d$_6$) δ:** 23.26; 25.06; 31.61; 75.64; 145.97; 157.33; 160.48. **HRMS:** for $C_{10}H_{16}N_4O_2$ m/z $[M+H]^+$ calculated: 225.1352; found: 225.1380. **LC** 1-(cyclohexyloxymethyl)-1,2,4-triazole-3-carboxamide content: spectrophotometric detection of 235 nm no less than 96%.

1-([2-Hydroxyethoxy]methyl)-1,2,4-triazole-3-carboxamide (1c)

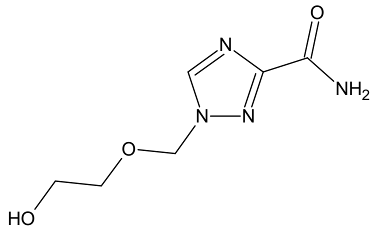

R$_f$ = 0.35 (1% CH_3OH in chloroform), **mp** 154-156°C. **^{1}H NMR spectrum (DMSO-d$_6$) δ:** 3.44-3.55 (m, 4H, -O$\underline{CH_2CH_2}$O-); 5.59 (s, 2H, OCH_2); 7.57 and 7.79 (2s, 2H, NH_2); 8.79 (s, 1H, CH). **^{13}C NMR spectrum (DMSO-d$_6$) δ:** 59.79; 70.98; 78.09; 146.01; 158.36; 160.39. **HRMS:** for $C_6H_{10}N_4O_3$ m/z $[M+H]^+$ calculated: 187.0831; found: 187.0838; **LC** 1-([2-hydroxyethoxy]methyl))-1,2,4-triazole-3-carboxamide content: spectrophotometric detection of 235 nm no less than 97%.

1-(Phenoxymethyl)-1,2,4-triazole-3-carboxamide (11k)

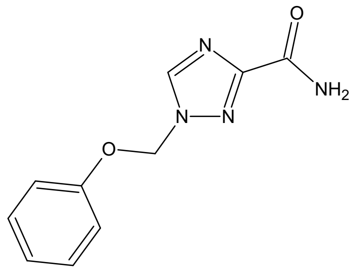

R_f = 0.75 (1% CH_3OH in chloroform), **mp** 188-192°C **^{1}H NMR spectrum (DMSO-d$_6$) δ:** 6.23 (s, 2H, OCH_2); 7.01-7.35 (m, 5H, Ph); 7.67 and 7.90 (2s, 2H, NH_2); 8.79 (s, 1H, CH). **^{13}C NMR spectrum (DMSO-d$_6$) δ:** 75.02; 116.03; 122.63; 129.82; 146.75; 155.74; 160.26. **HRMS:** for $C_{10}H_{10}N_4O_2$ m/z $[M+H]^+$ calculated: 219.0882; found: 219.0896; **LC** 1-(phenoxymethyl)-1,2,4-triazole-3-carboxamide content: spectrophotometric detection of 235 nm no less than 95%.

Copies of NMR and MS spectra:

5-(*n*-Propoxymethyl)-1,2,4-triazole-3-carboxamide (6c)

^{1}H NMR spectrum (**6c**)

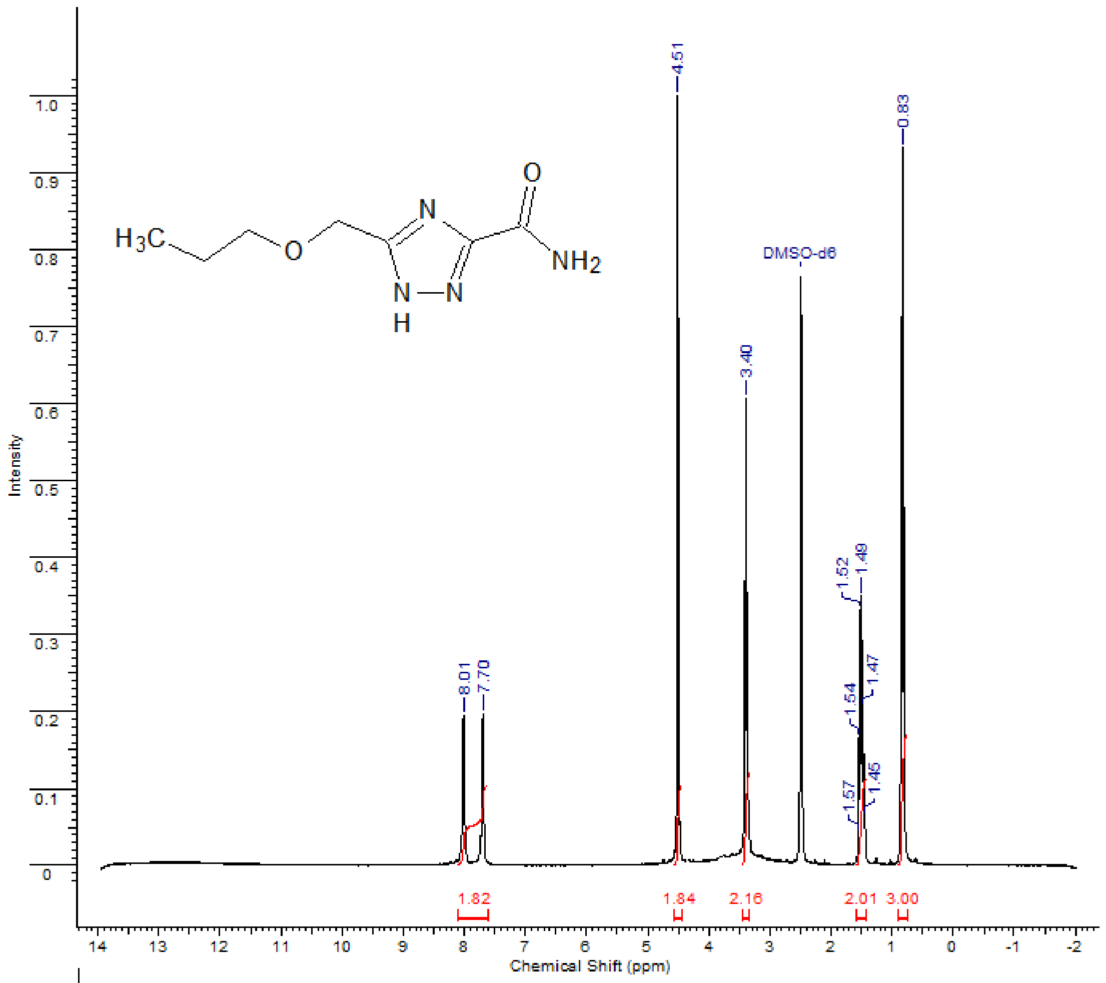

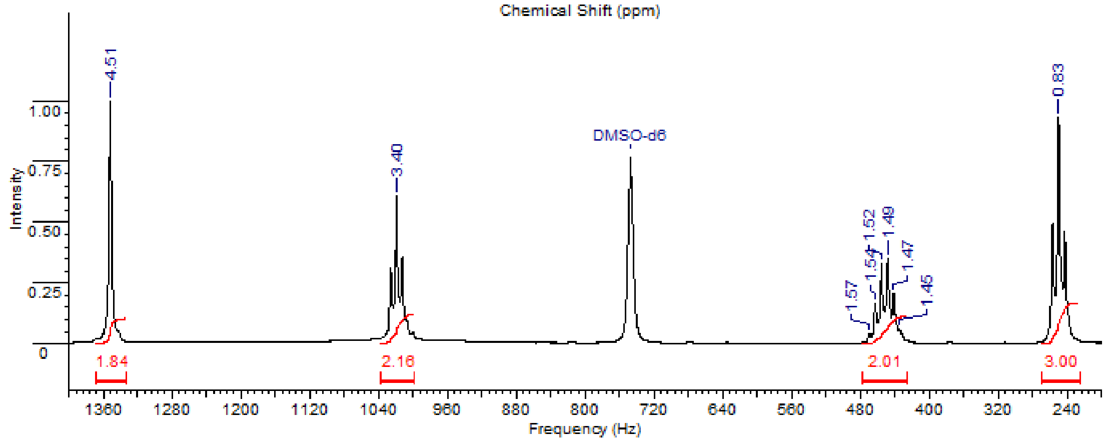

^{13}C NMR spectrum (**6c**)

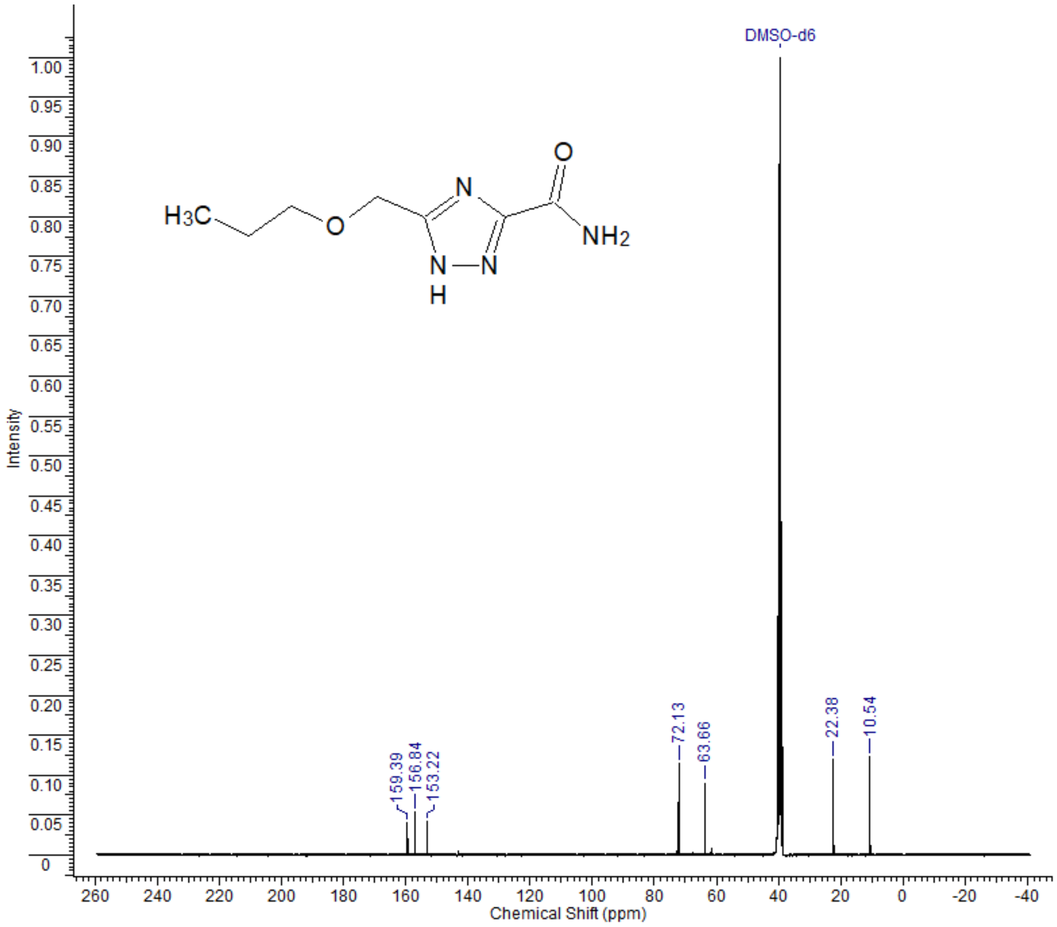

MS ESI+ (**6c**) (M=184 Da)

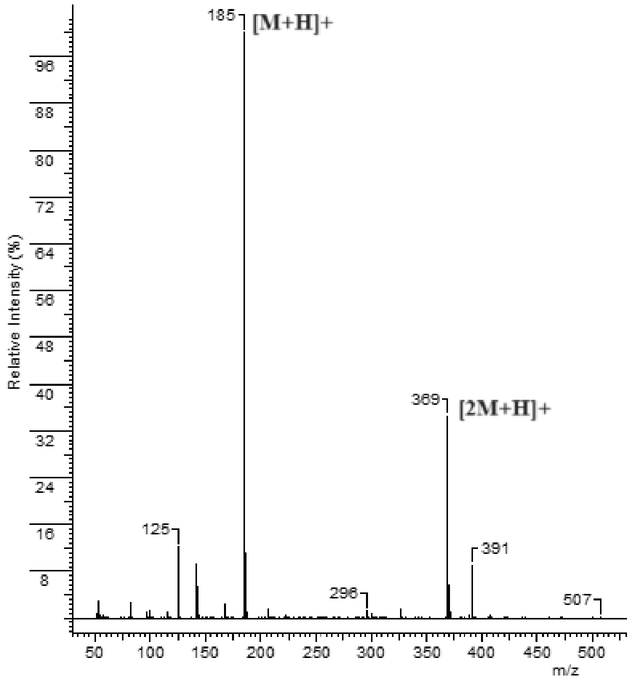

Methyl 1-(Methoxymethyl)-1,2,4-triazole-3-carboxylic acid (10a)

^{1}H NMR spectrum (**10a**)

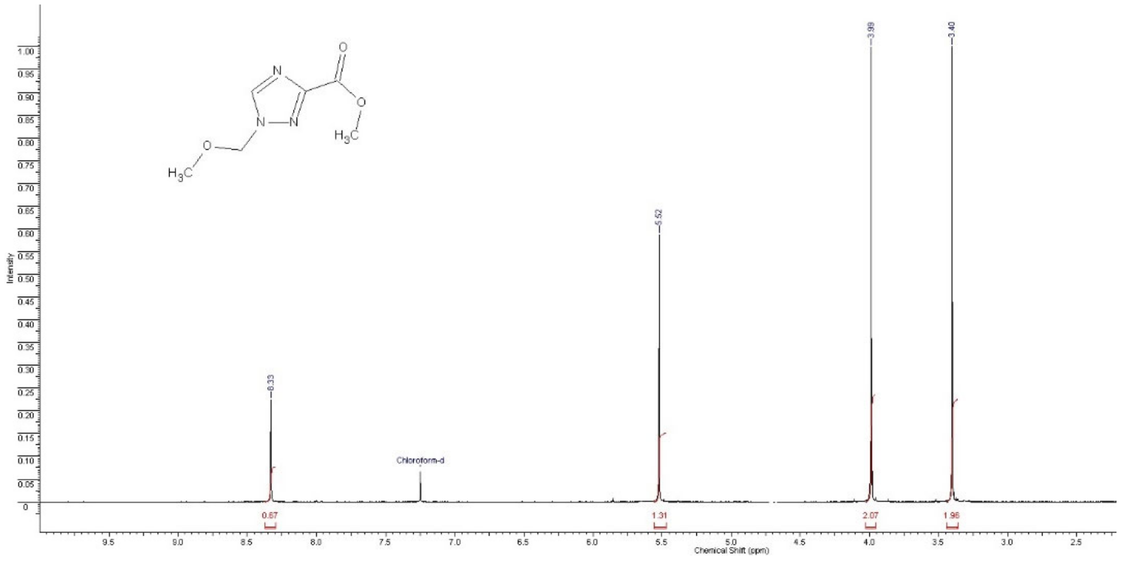

^{13}C NMR spectrum (**10a**)

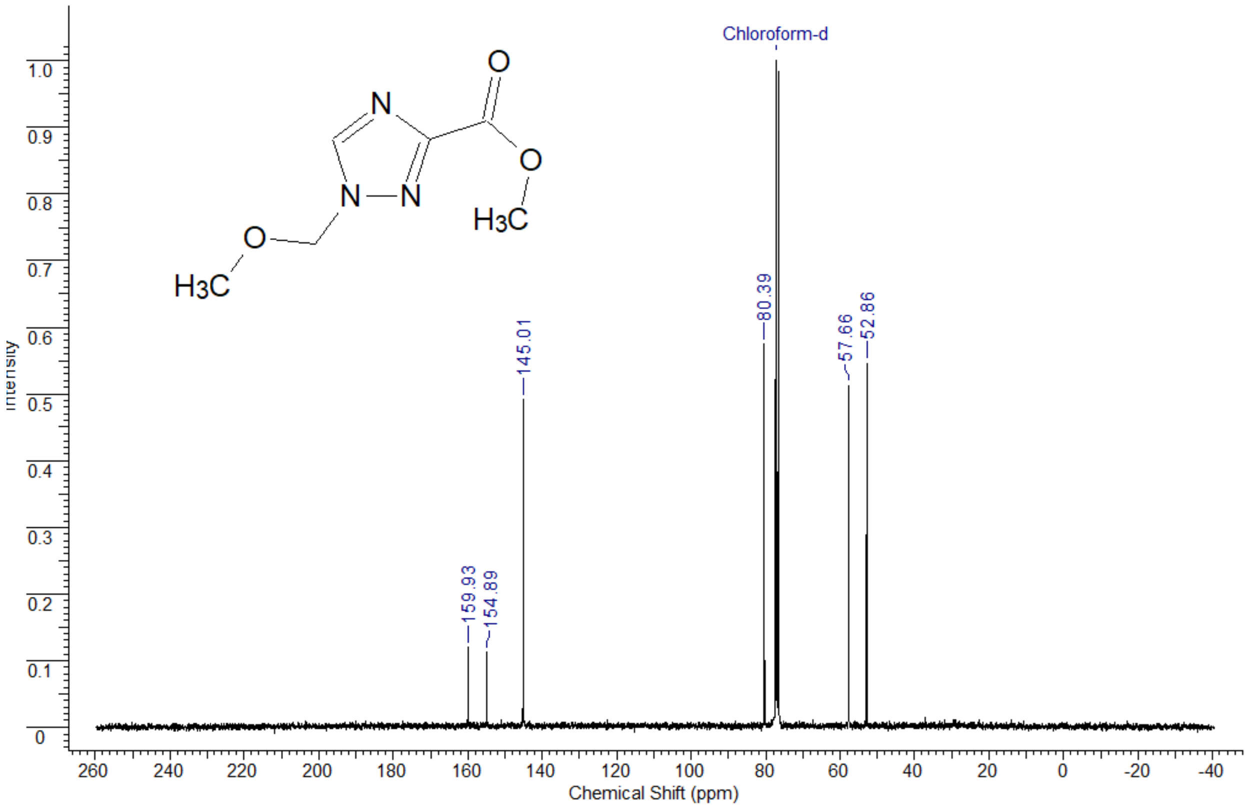

^{13}C APT NMR spectrum (**10a**)

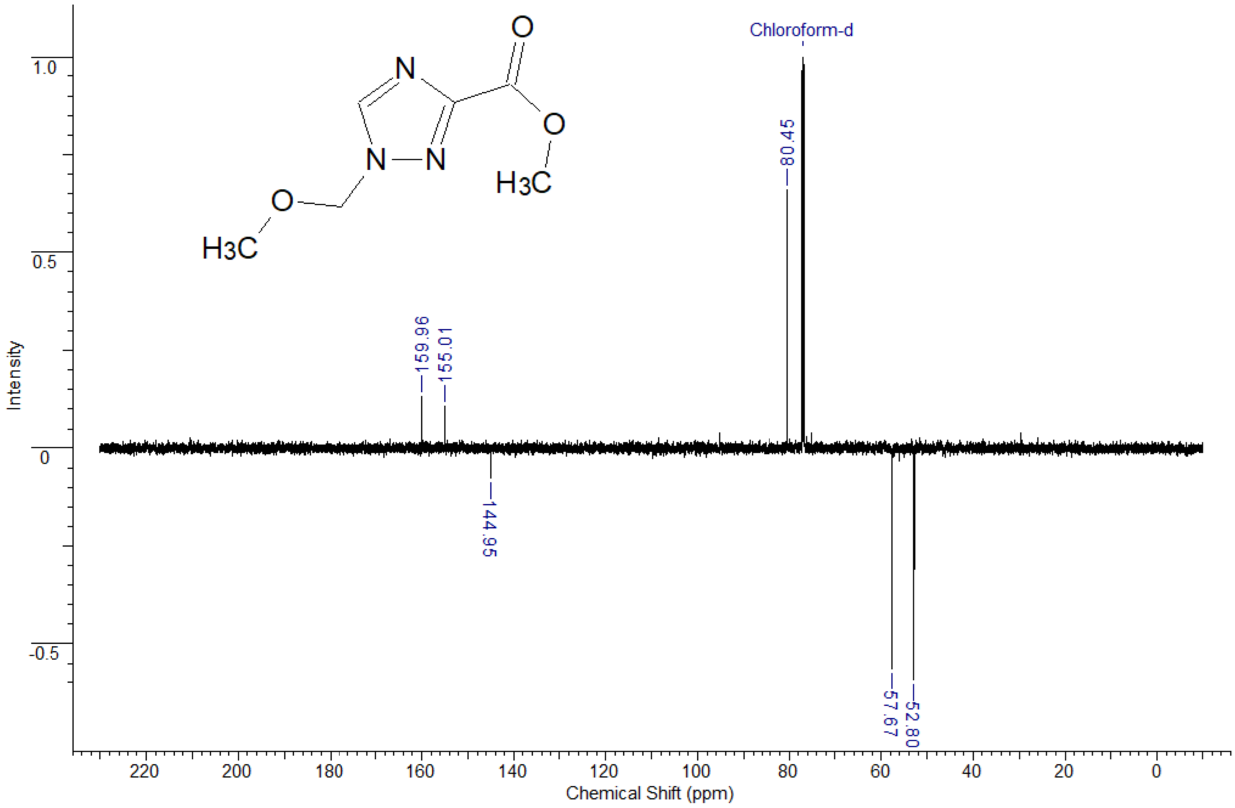

^{1}H-^{13}C HMBC NMR spectrum (**10a**)

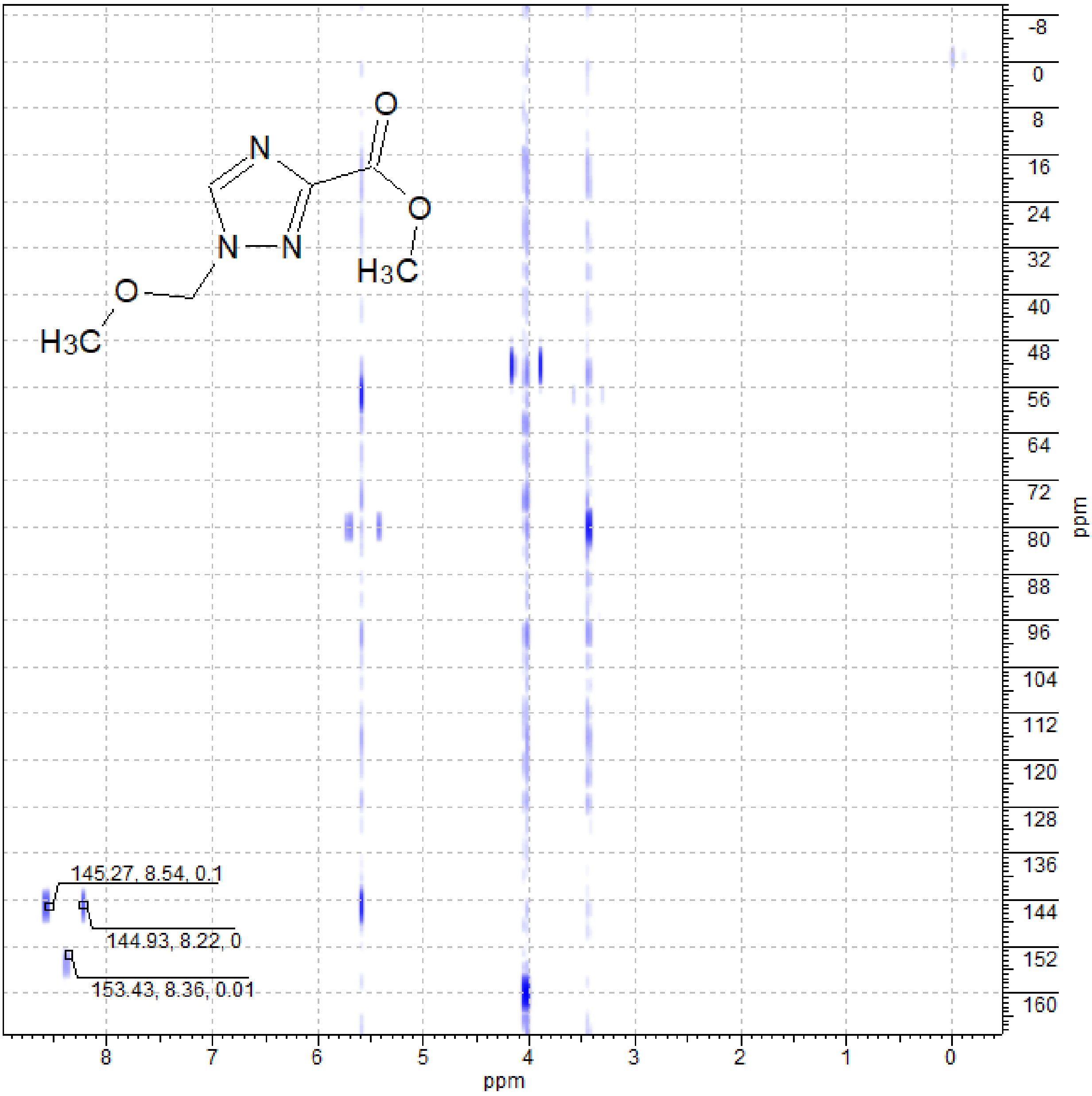

1-(Methoxymethyl)-1,2,4-triazole-3-carboxamide (11a)

^{1}H NMR spectrum (**11a**)

In the spectrum, the signal of water coincides with the signal from compound **11a** (Chemical Shift 3.29).

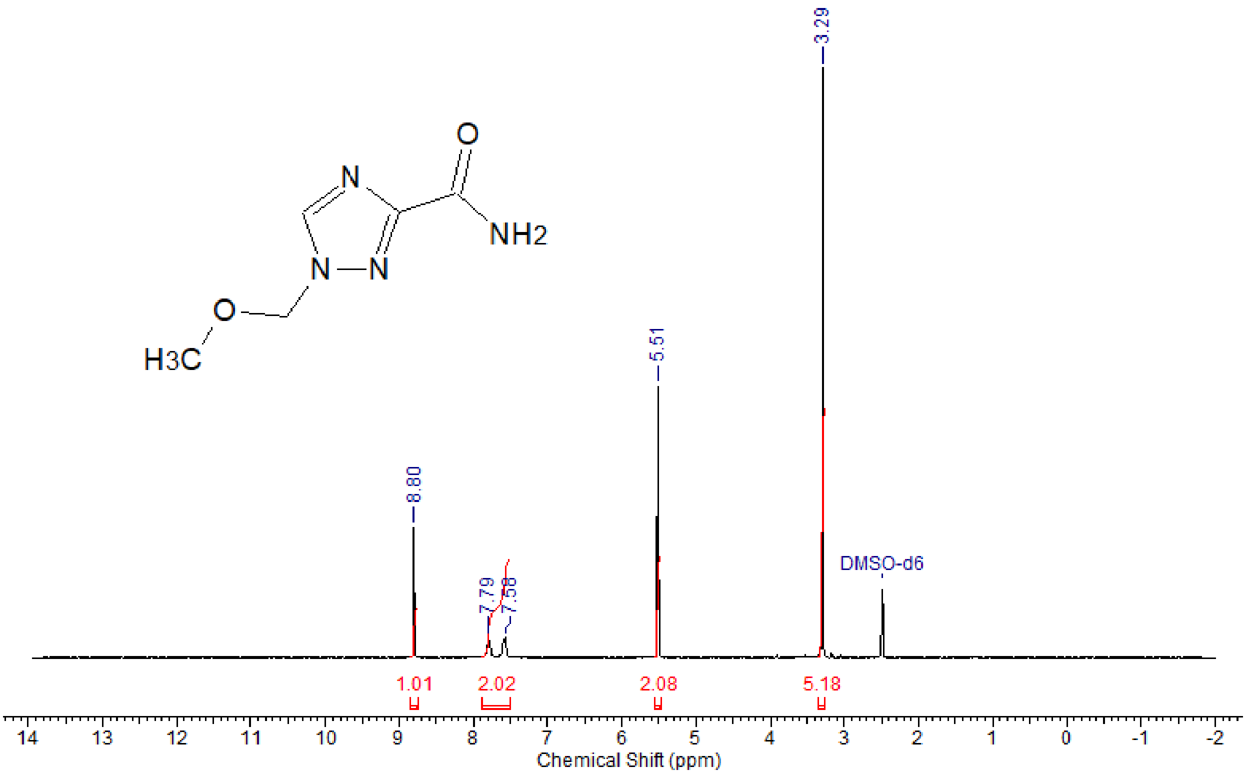

^{13}C NMR spectrum (**11a**)

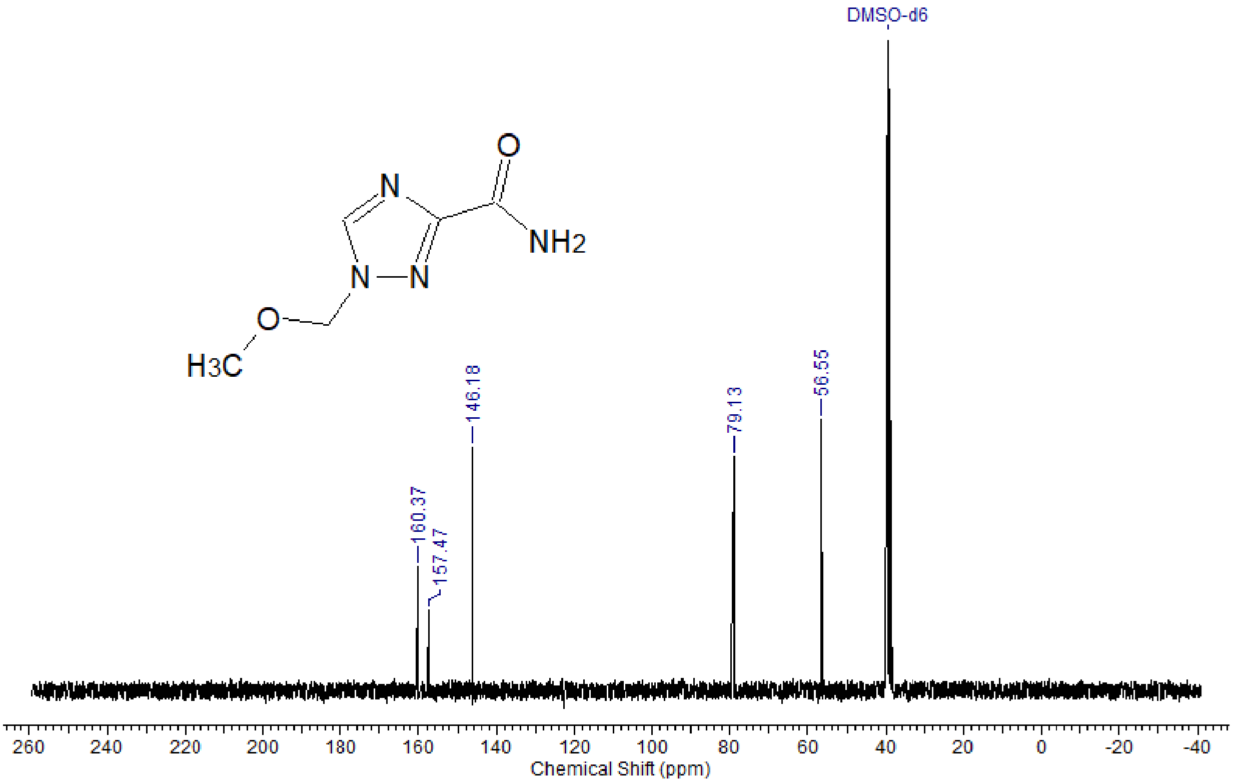

MS ESI+ (**11a**) (M=156 Da)

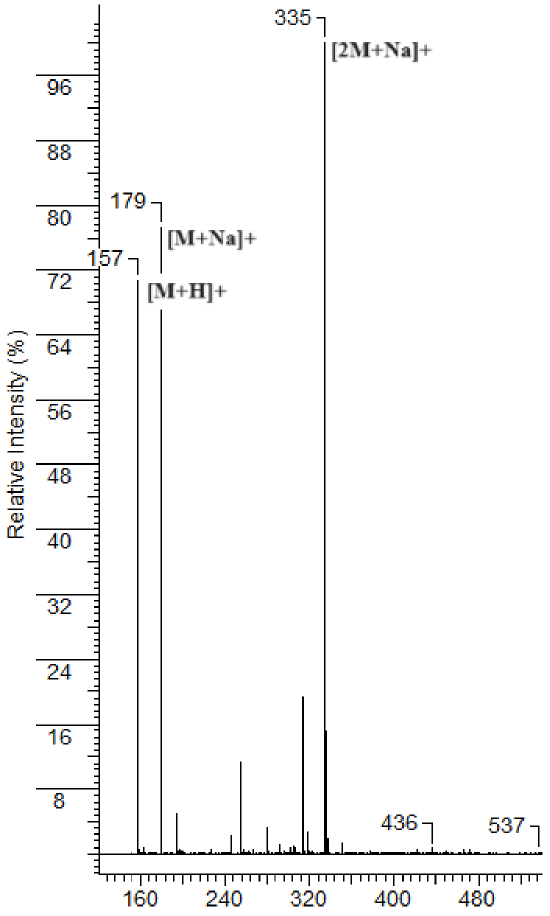

1-(Ethoxymethyl)-1,2,4-triazole-3-carboxamide (11b)

<u>^{1}H NMR spectrum (**11b**)</u>

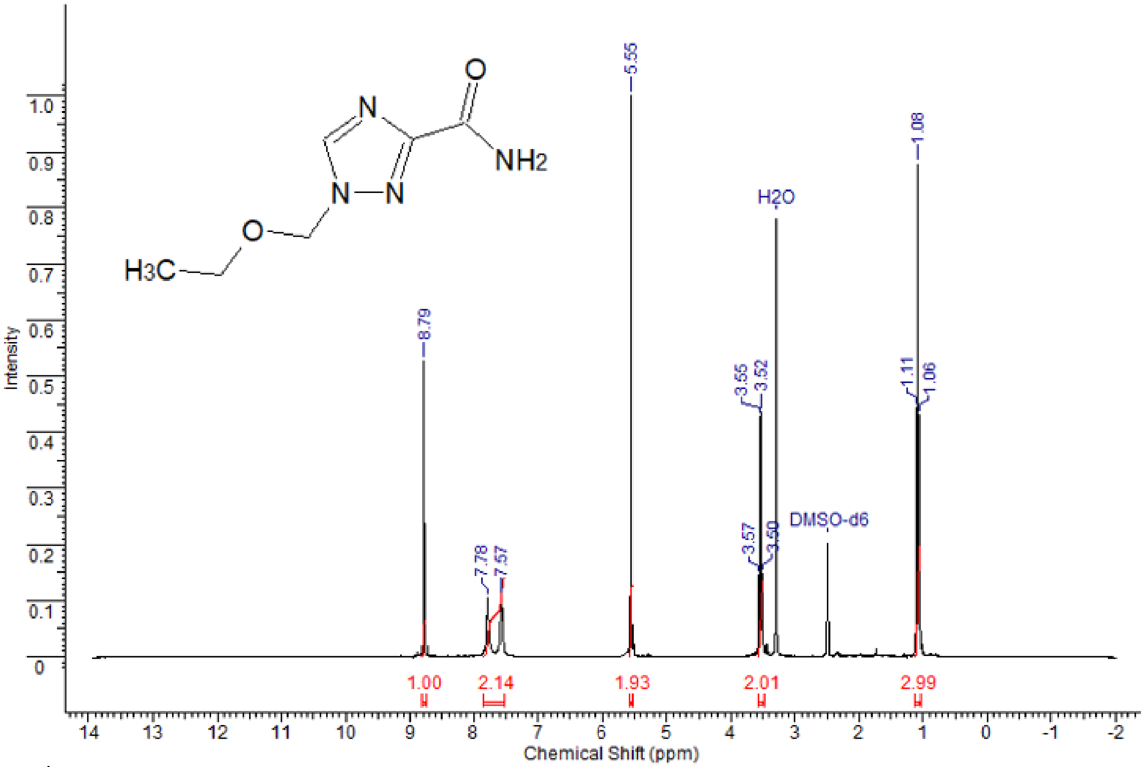

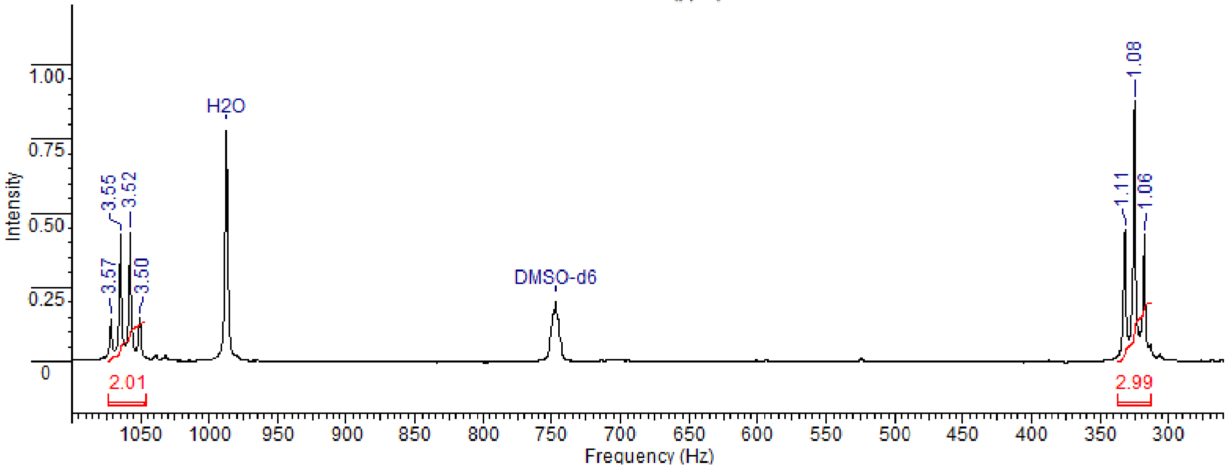

^{13}C NMR spectrum (**11b**)

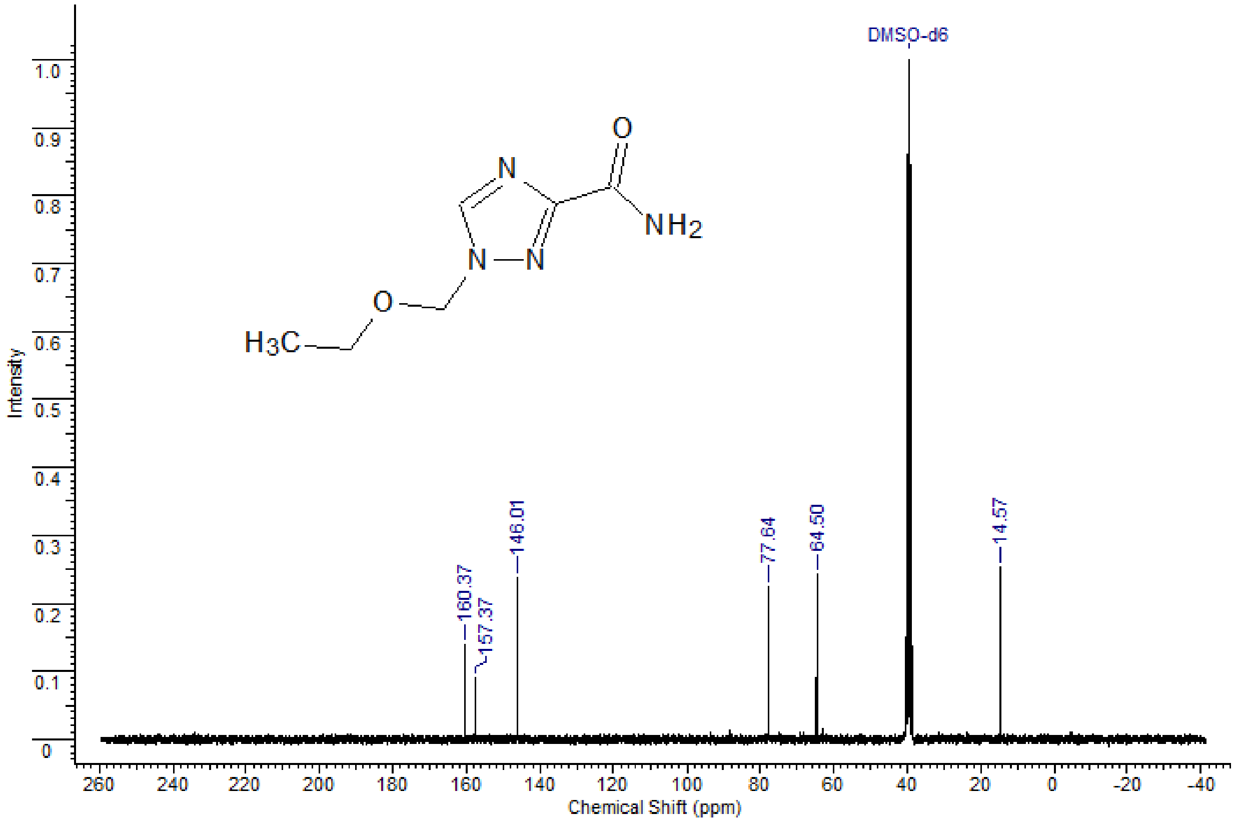

MS ESI+ (**11b**) (M=170 Da)

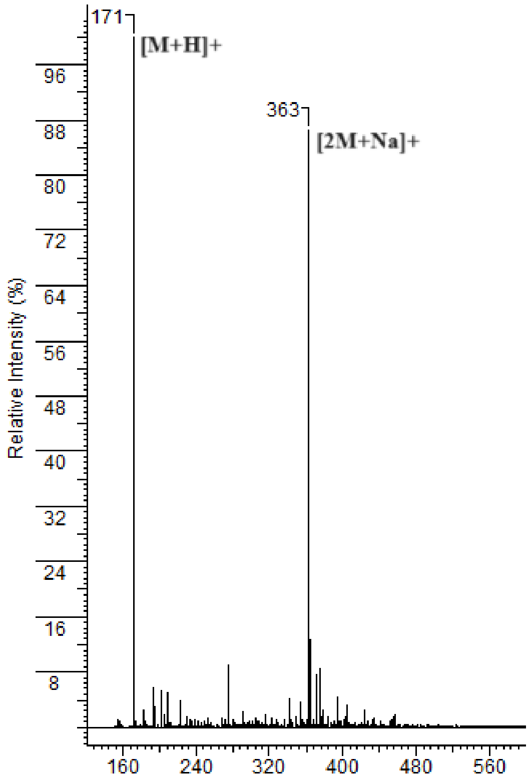

1-(*n*-Propoxymethyl)-1,2,4-triazole-3-carboxamide (11c)

^{1}H NMR spectrum (**11c**)

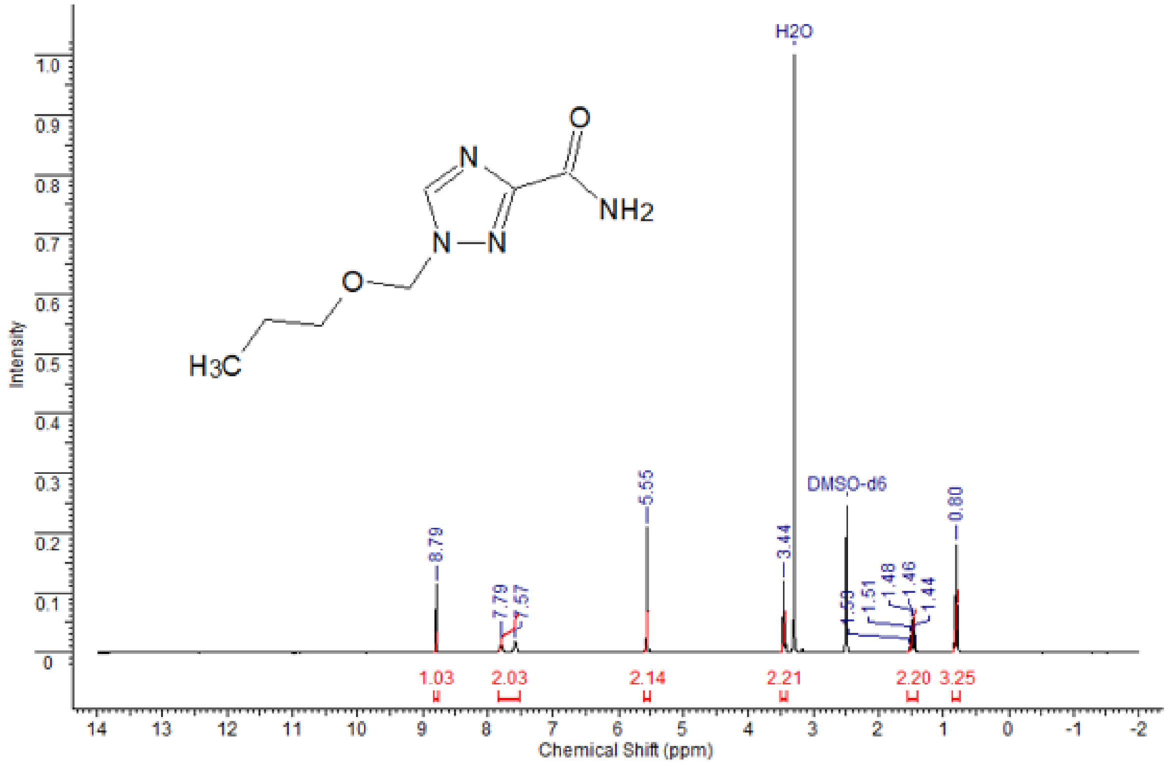

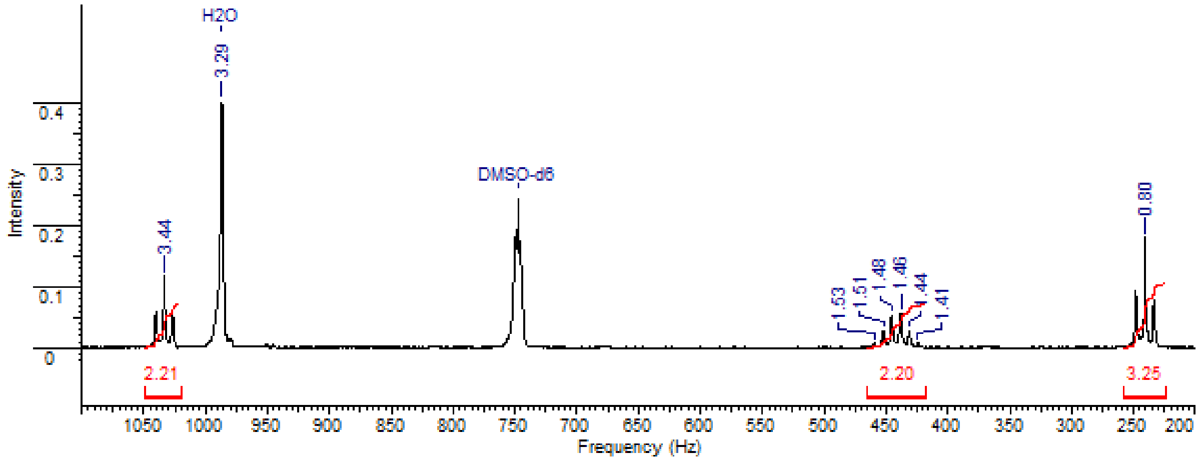

^{13}C NMR spectrum (**11c**)

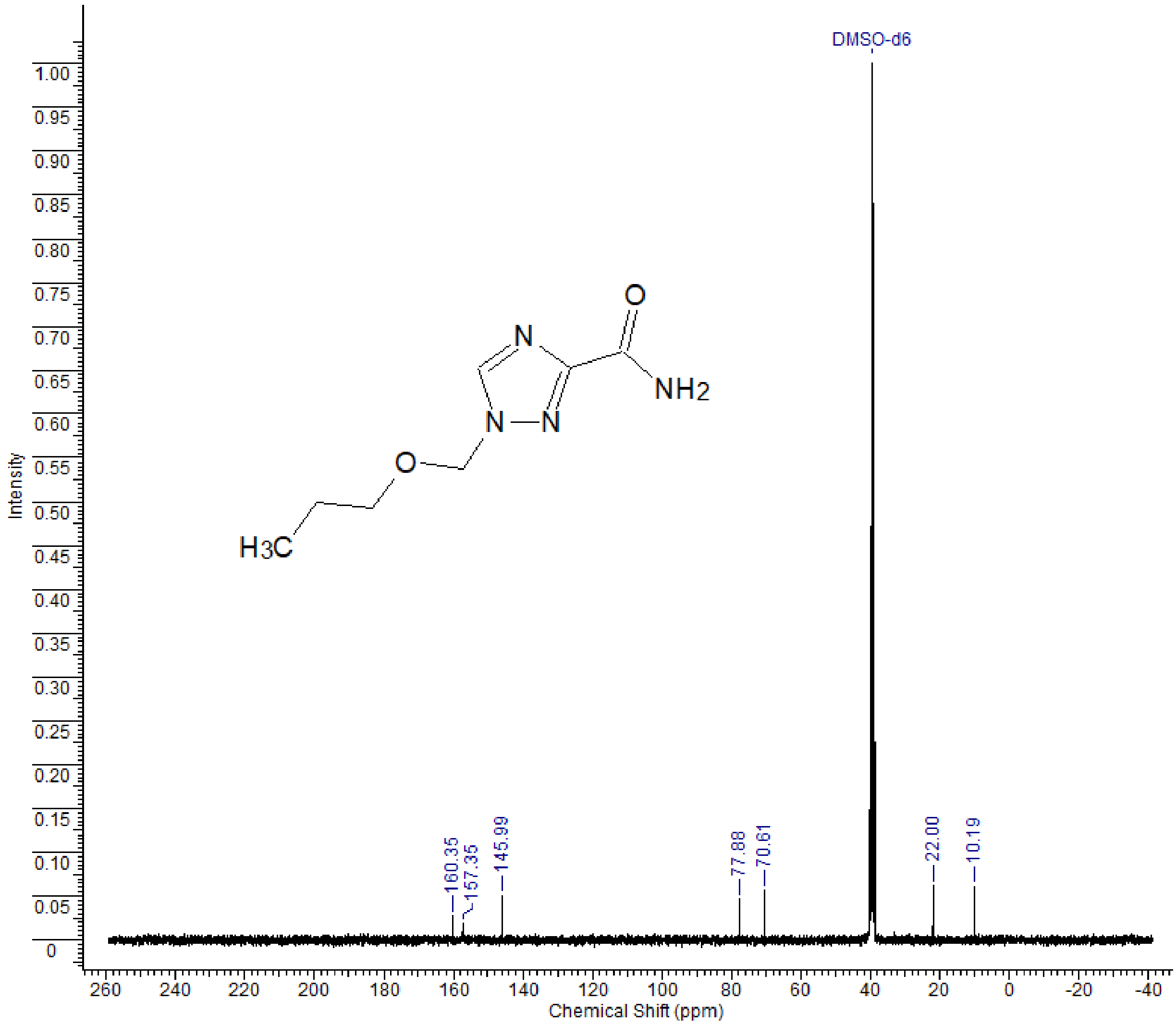

MS ESI+ (**11c**) (M=185 Da)

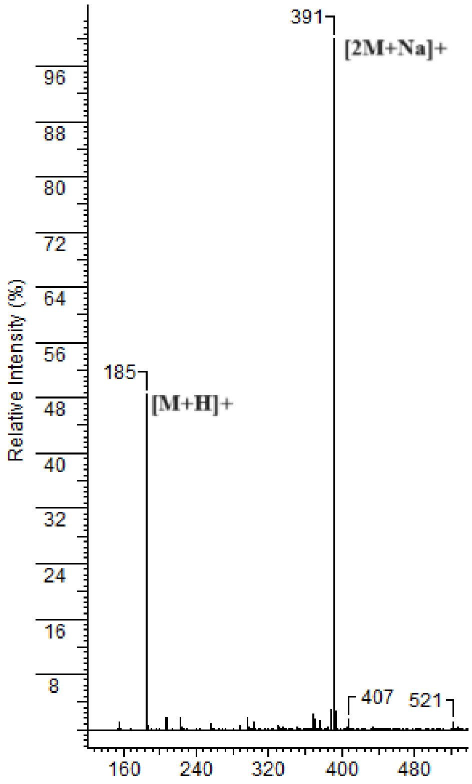

1-(Isopropoxymethyl)-1,2,4-triazole-3-carboxamide (11d)

^{1}H NMR spectrum (**11d**)

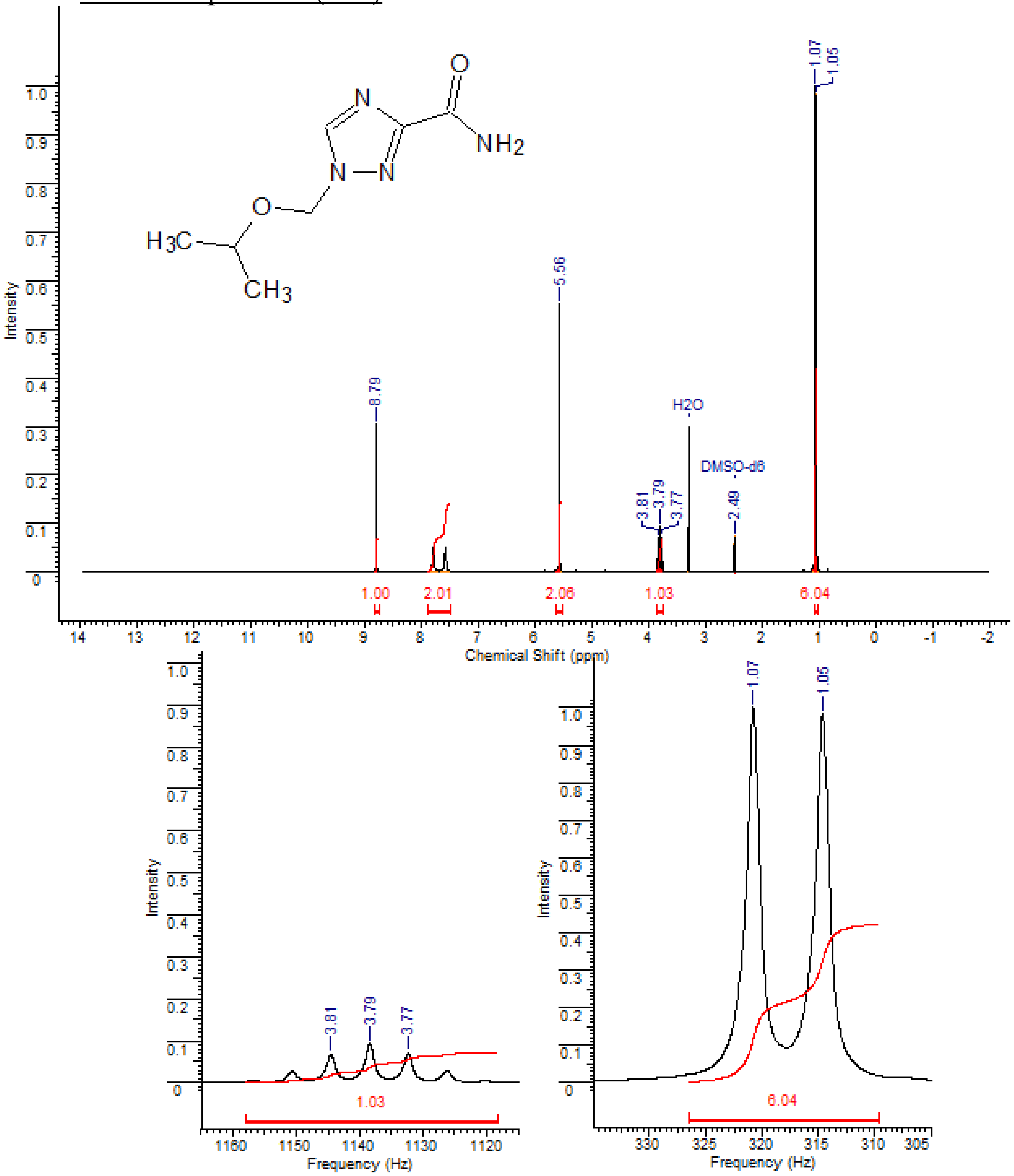

^{13}C NMR spectrum (**11d**)

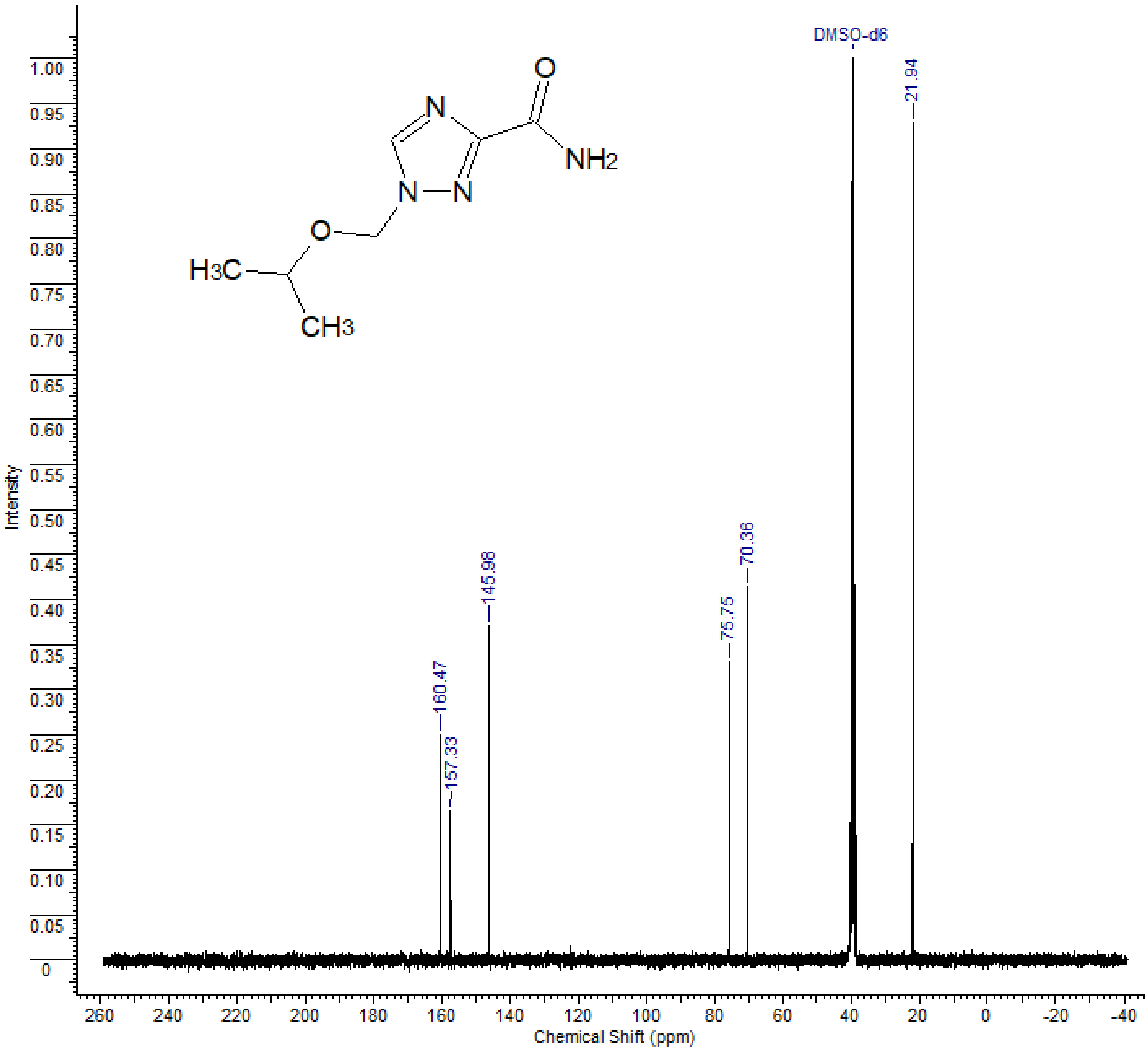

MS ESI+ (**11d**) (M=184 Da)

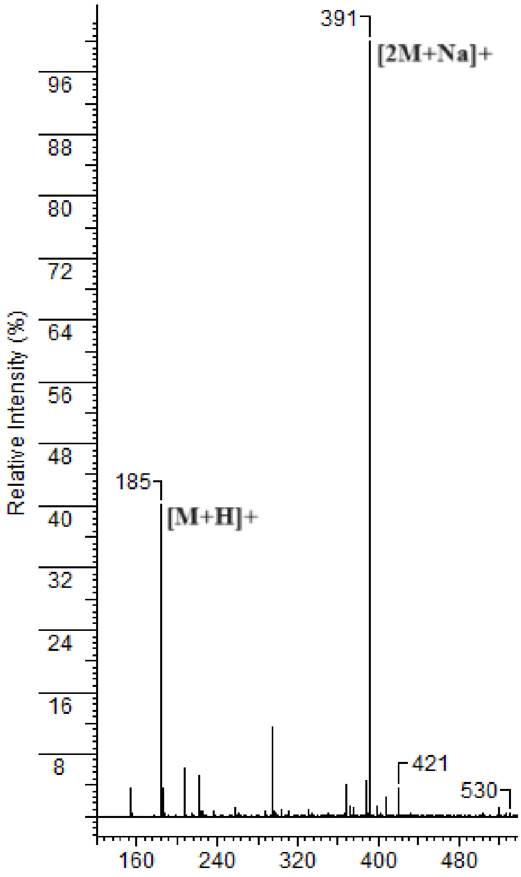

1-(*n*-Butyloxymethyl)-1,2,4-triazole-3-carboxamide (11e)

^{1}H NMR spectrum *(11e)*

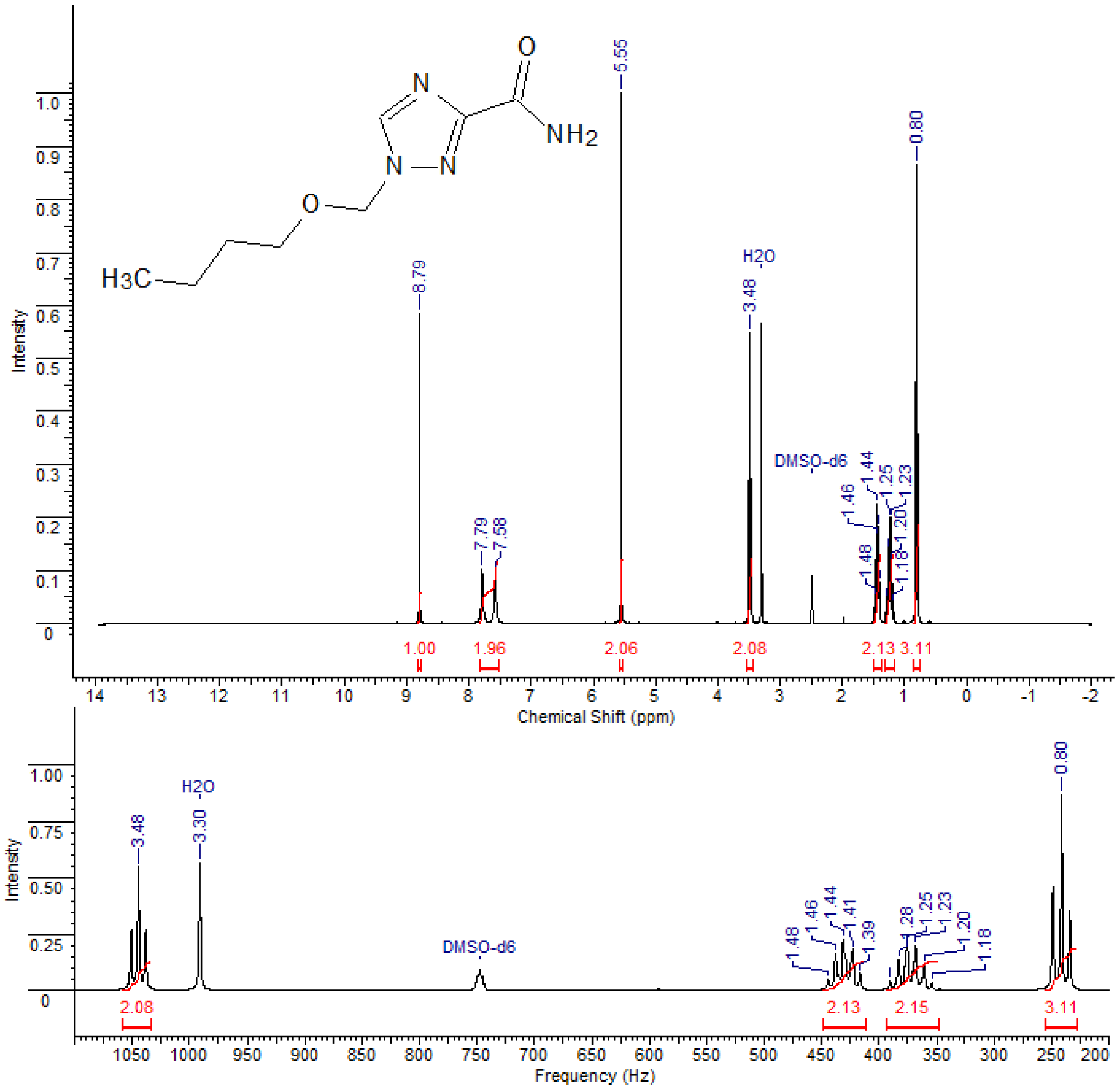

^{13}C NMR ***(11e)***

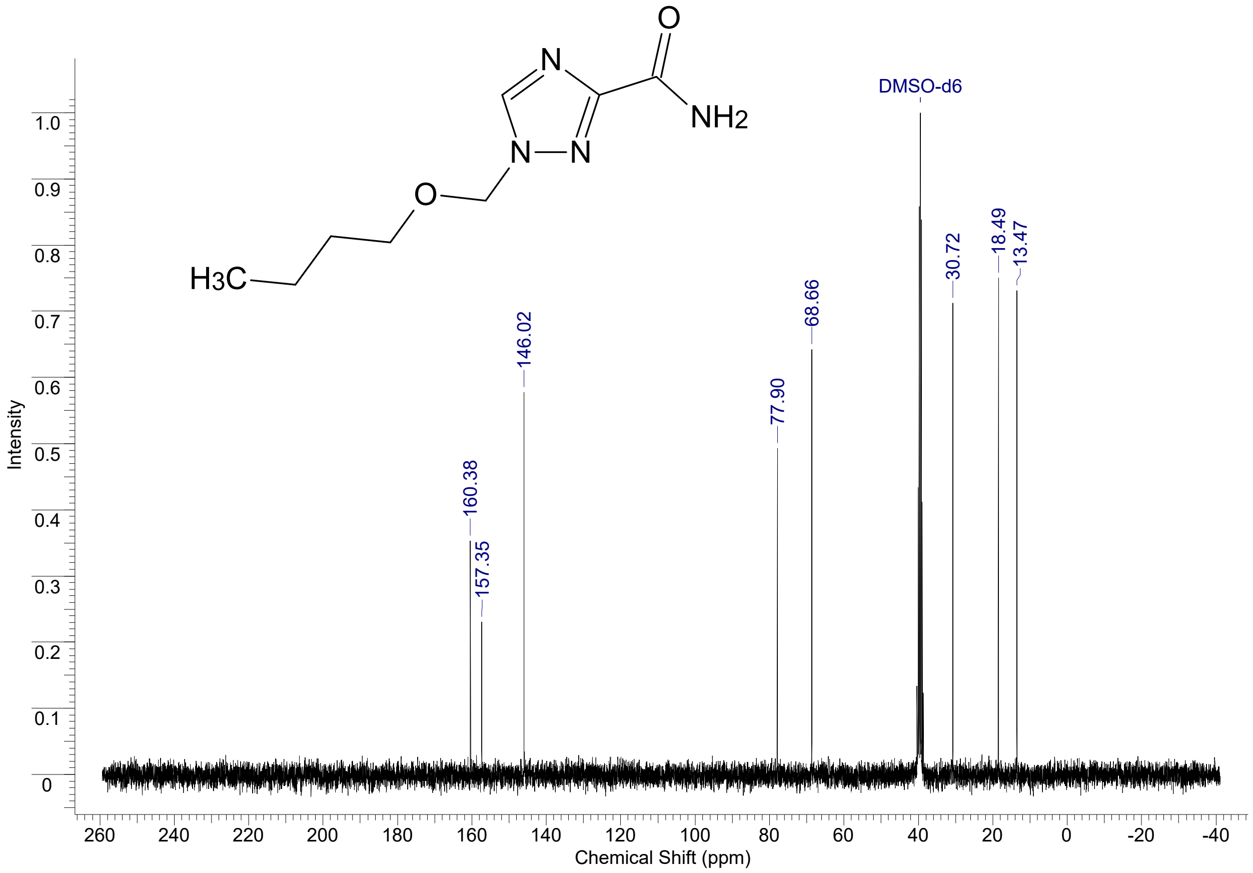

O
N
NH2
N
N
O
H3C
DMSO-d6
160.38
157.35
146.02
77.90
68.66
30.72
18.49
13.47
Intensity
1.0
0.9
0.8
0.7
0.6
0.5
0.4
0.3
0.2
0.1
0
260
240
220
200
180
160
140
120
100
80
60
40
20
0
-20
-40
Chemical Shift (ppm)

MS ESI+ (**11e**) (M=198 Da)

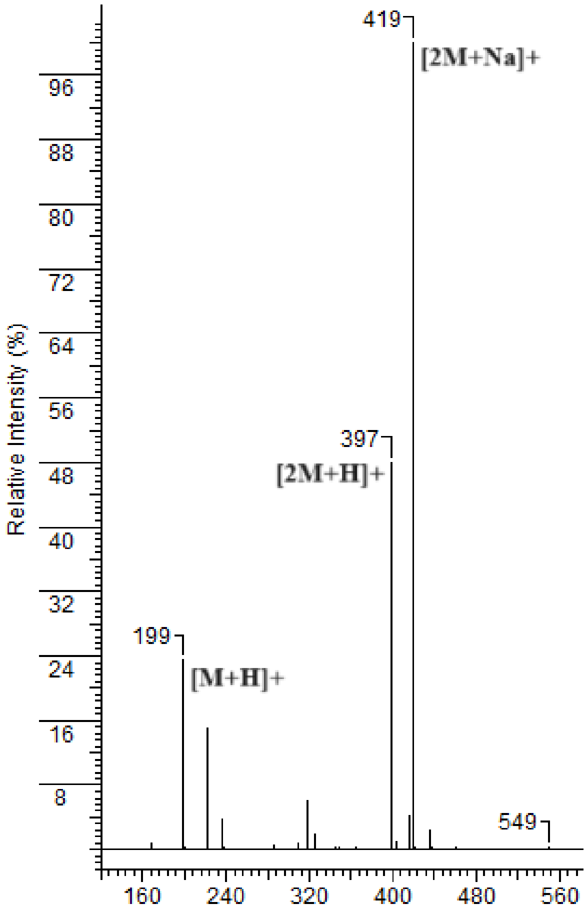

1-(*tert*-Butoxymethyl)-1,2,4-triazole-3-carboxamide (11f)

^{1}H NMR (**11f**)

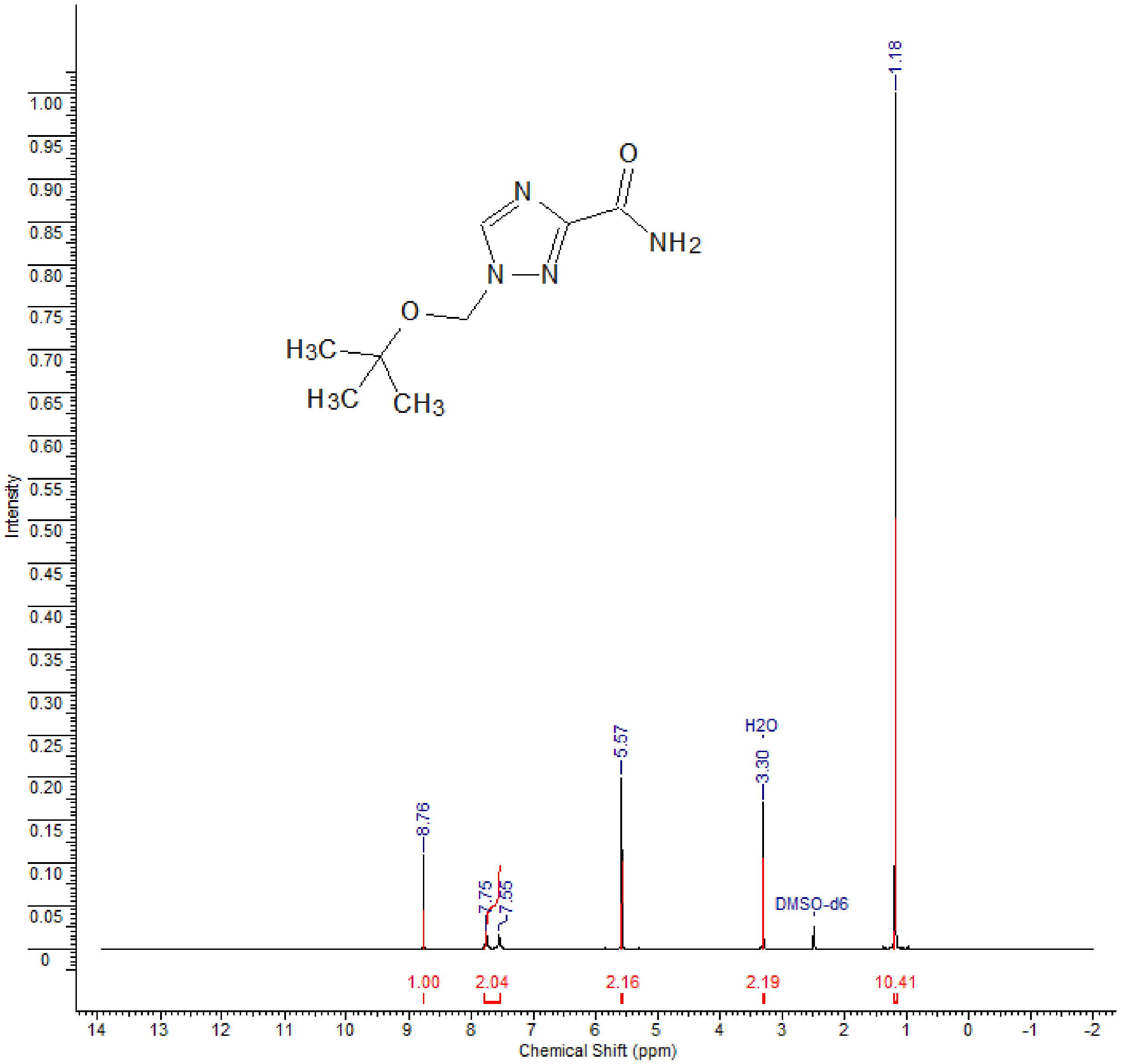

^{13}C NMR (**11f**)

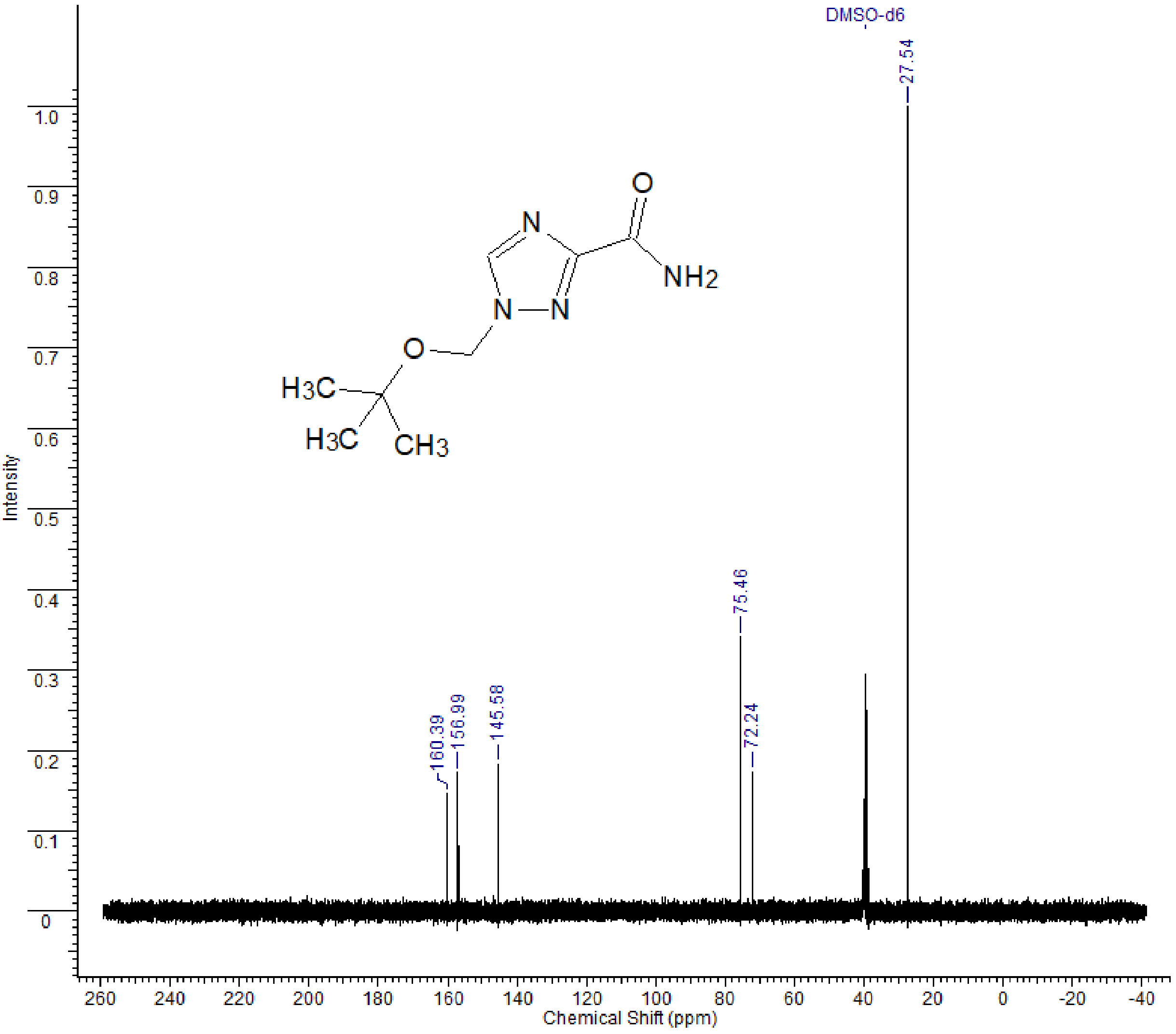
DMSO-d6
27.54
75.46
72.24
160.39
156.99
145.58
O
N
NH2
N
N
O
H3C
H3C
CH3
Intensity
1.0
0.9
0.8
0.7
0.6
0.5
0.4
0.3
0.2
0.1
0
260
240
220
200
180
160
140
120
100
80
60
40
20
0
-20
-40
Chemical Shift (ppm)

MS ESI+ (11f) (M=198 Da)

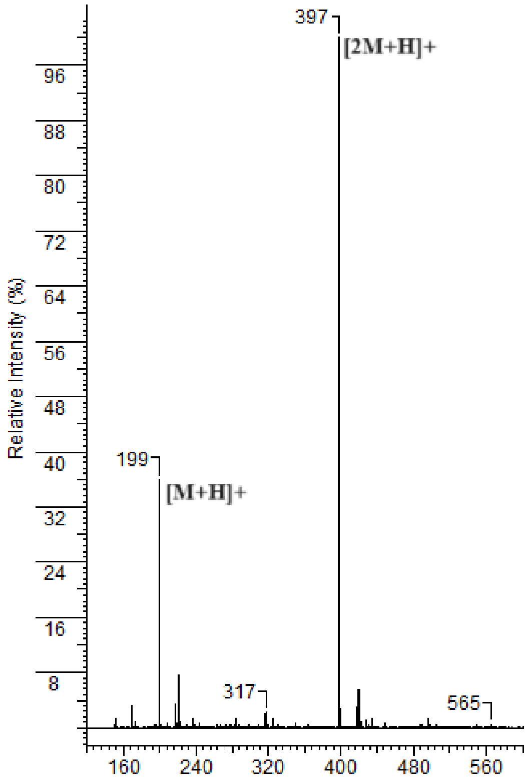

1-(*n*-Decyloxymethyl)-1,2,4-triazole-3-carboxamide (11g)

<u>^{1}H NMR spectrum (**11g**)</u>

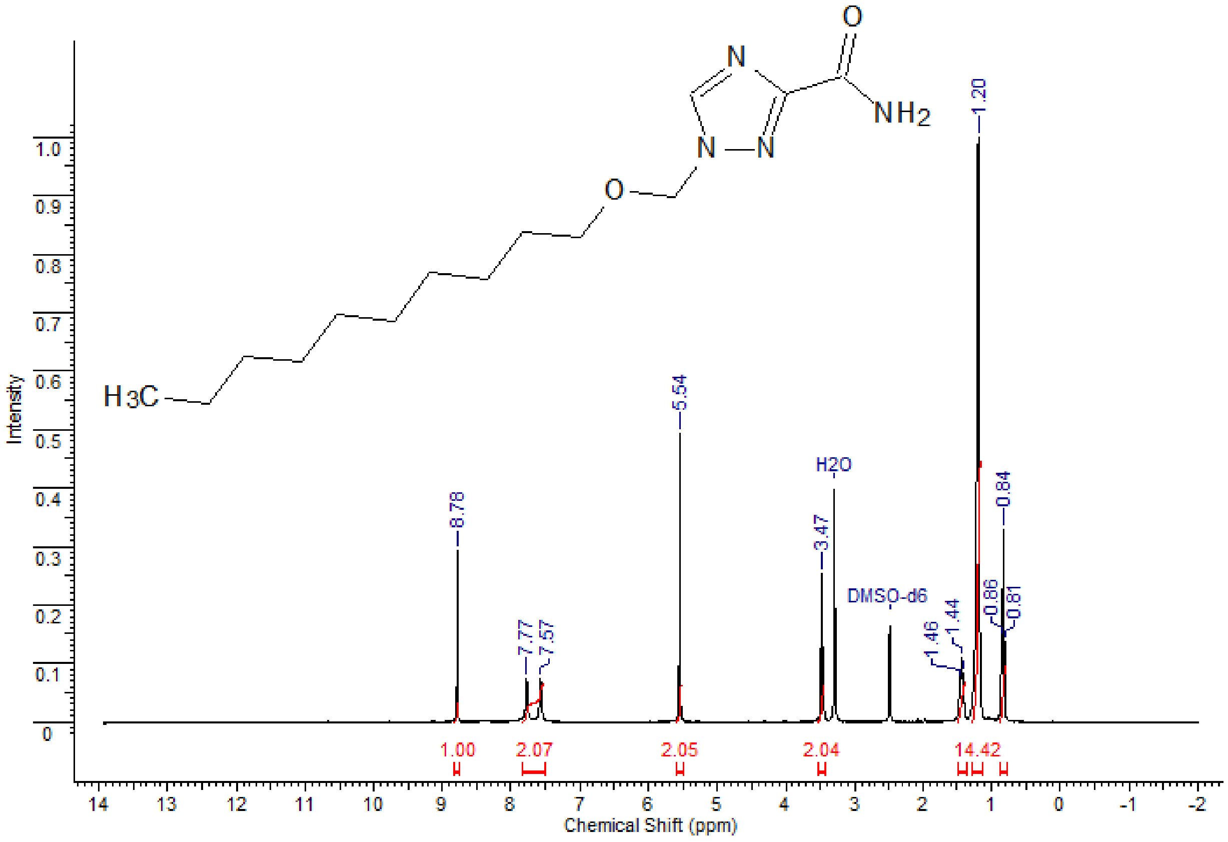

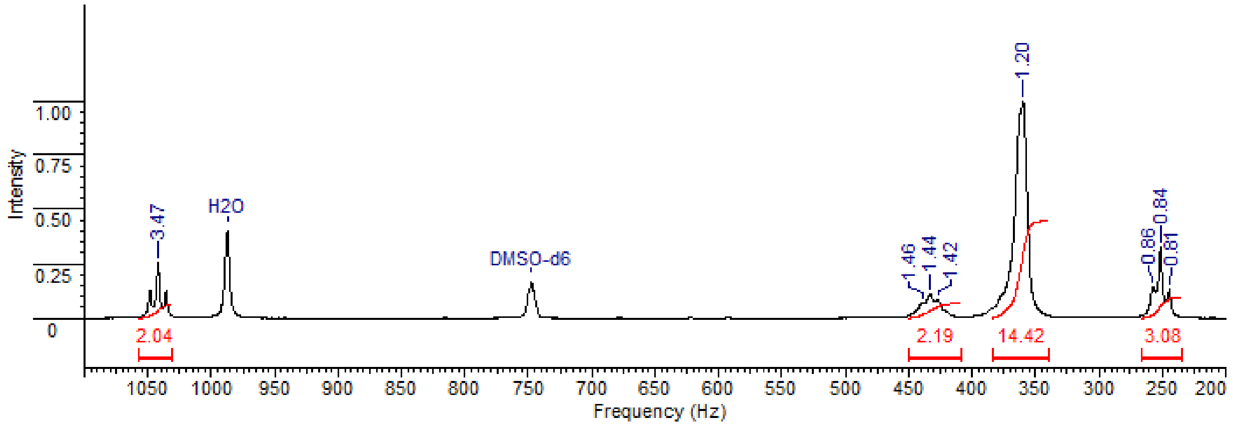

^{13}C NMR spectrum (**11g**)

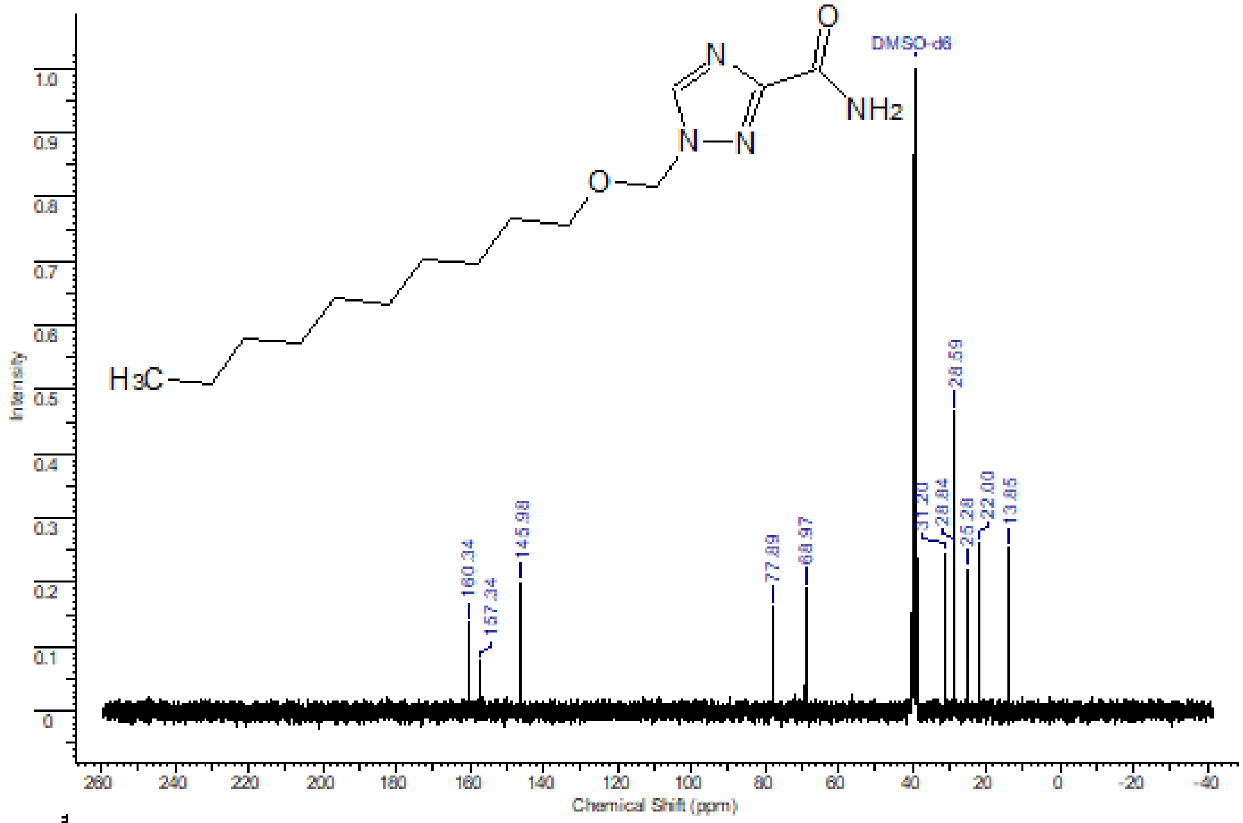

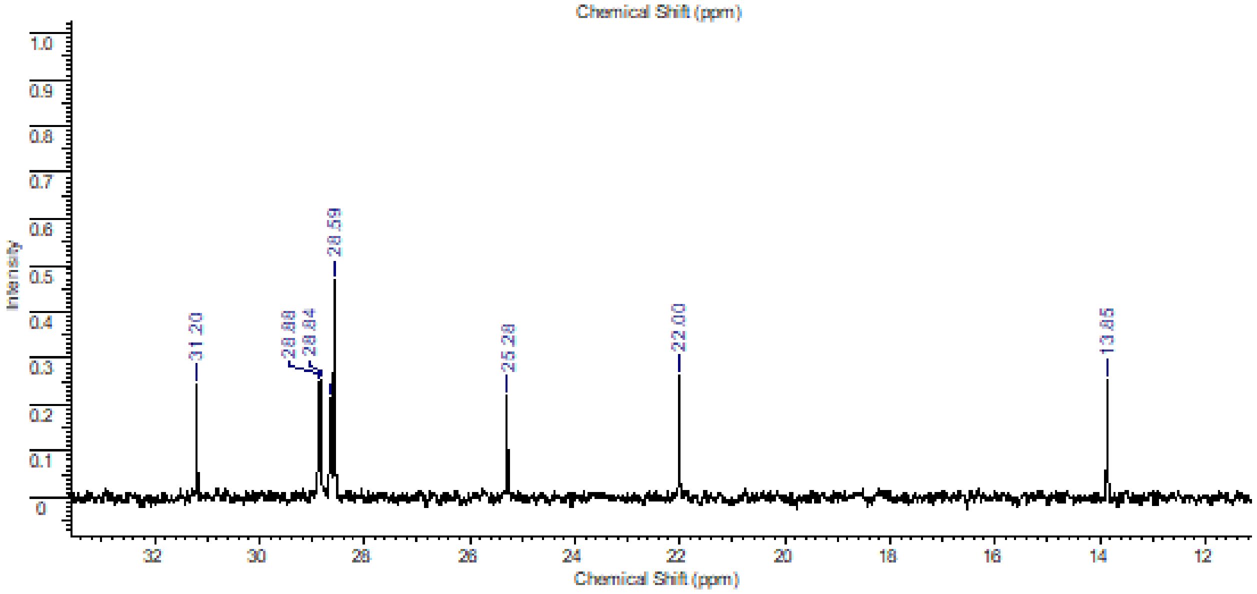

MS ESI+ (11g) (M=282 Da)

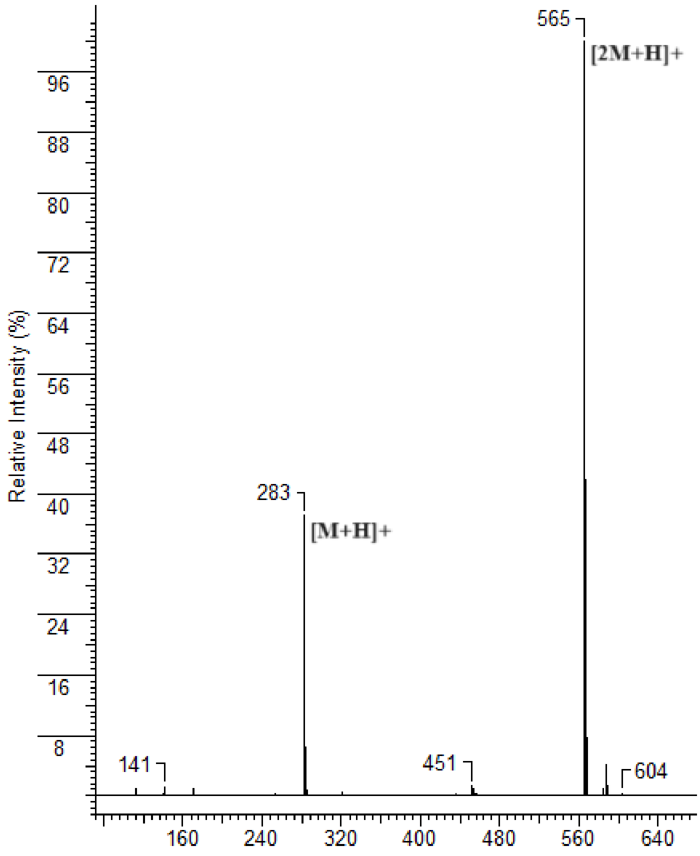

1-(Benzyloxymethyl)-1,2,4-triazole-3-carboxamide (11h)

<u>^{1}H NMR spectrum (**11h**)</u>

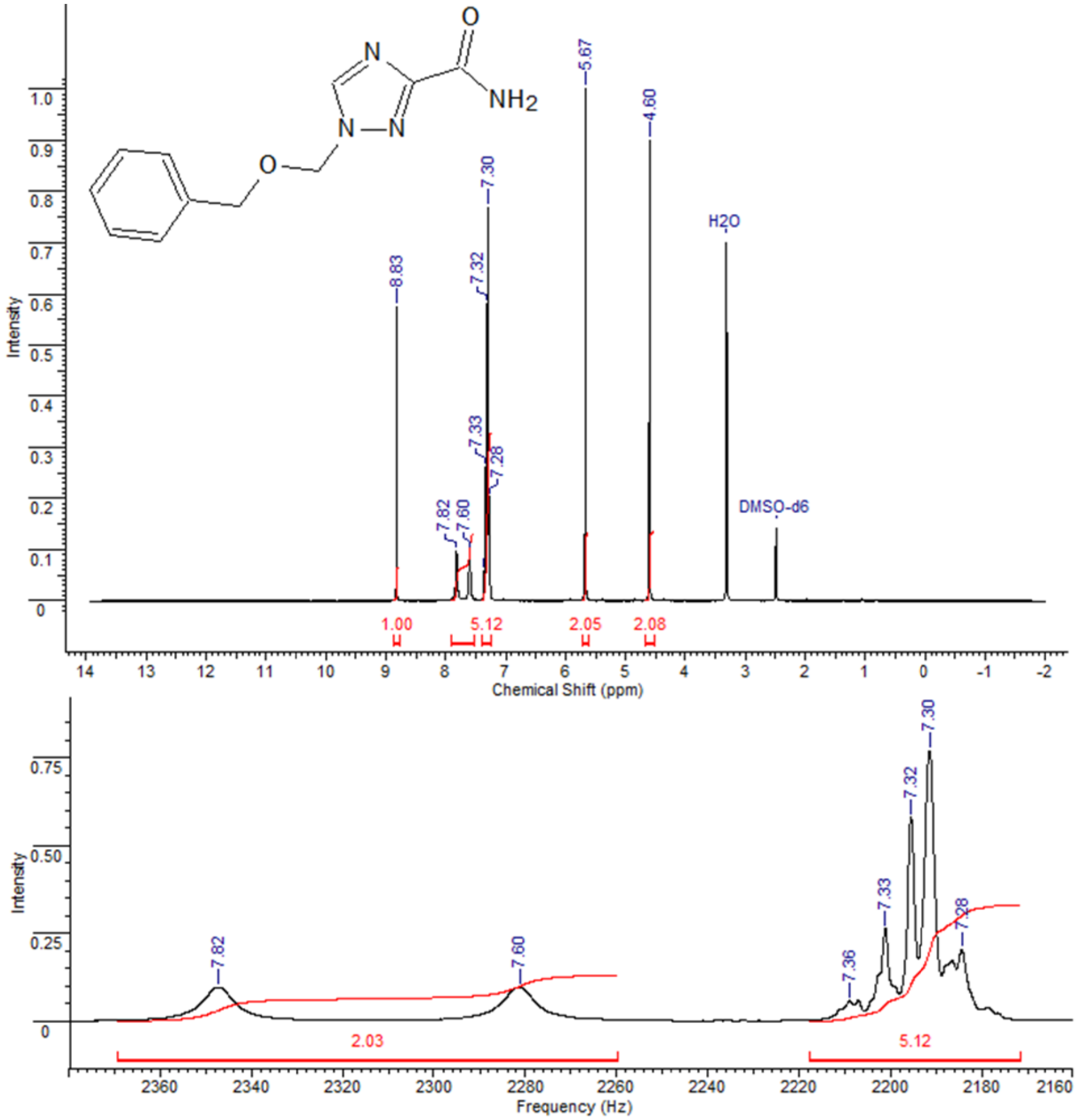

^{13}C NMR spectrum (**11h**)

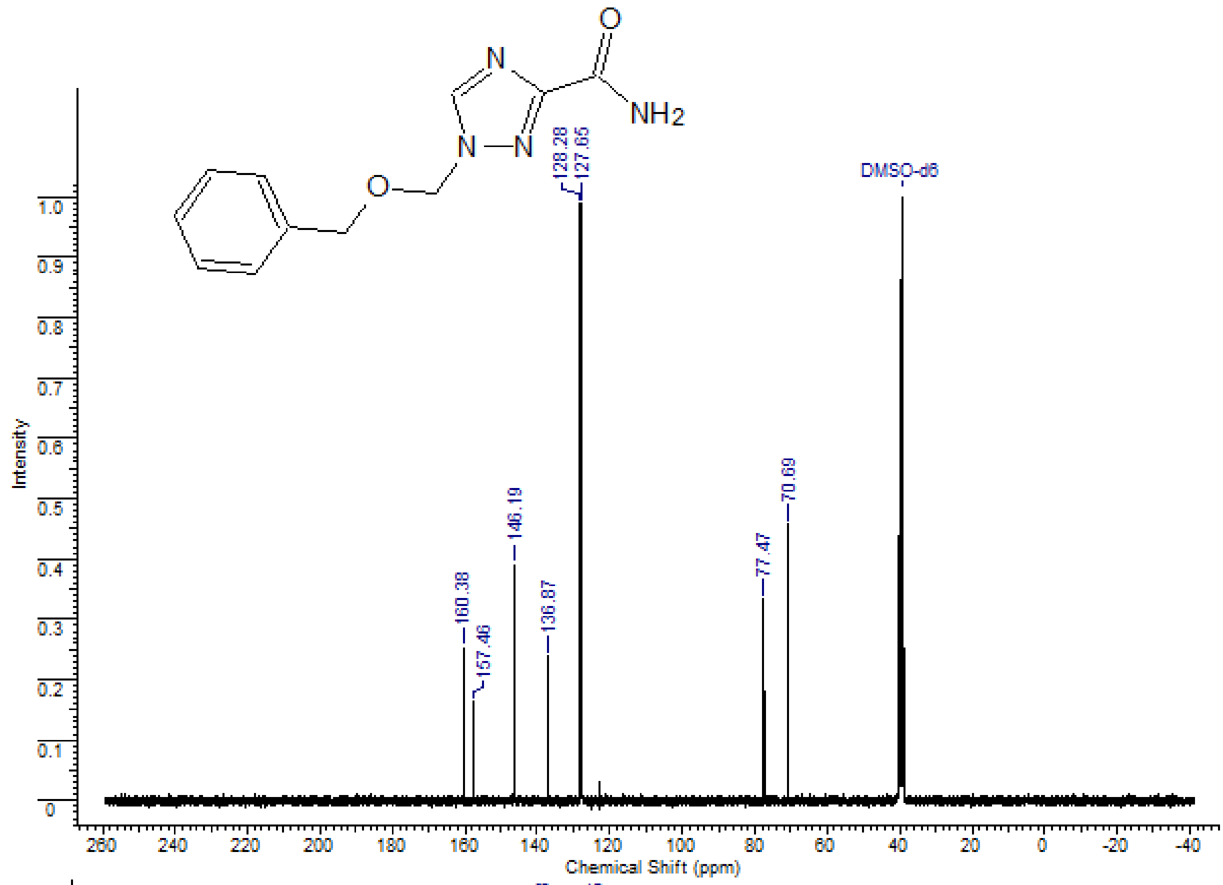

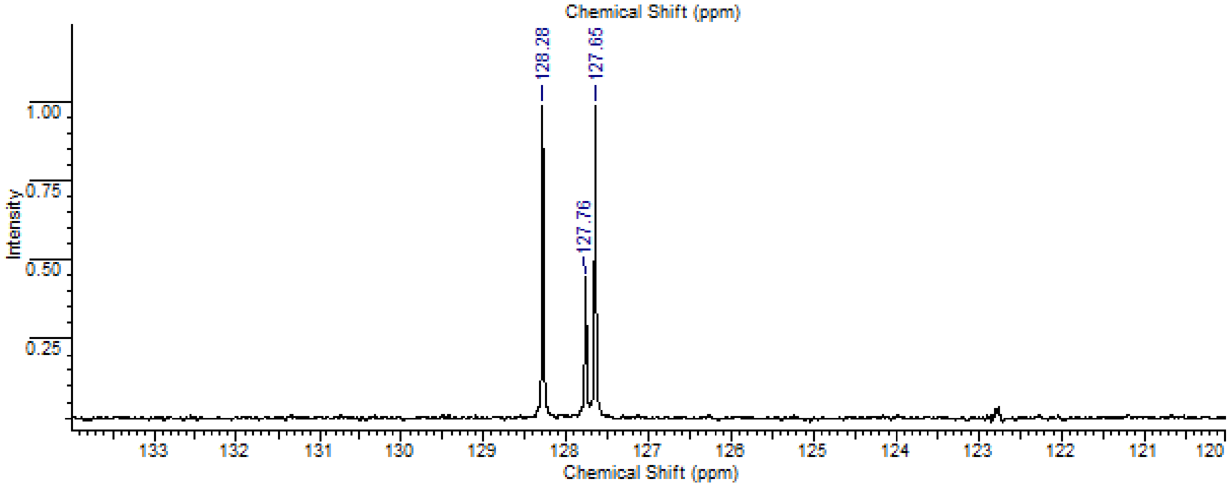

MS ESI+ (11h) (M=232 Da)

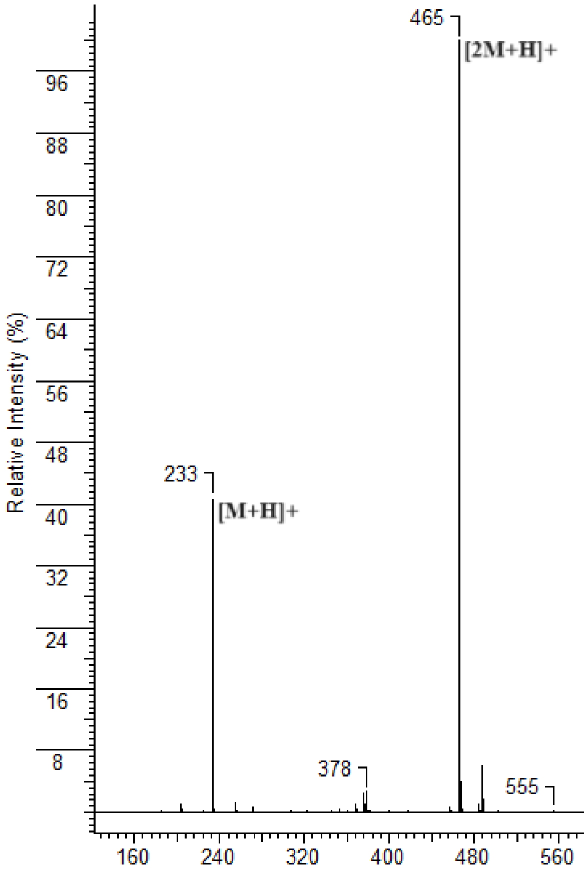

1-(Cyclopentyloxymethyl)-1,2,4-triazole-3-carboxamide (11i)

^{1}H NMR spectrum (**11i**)

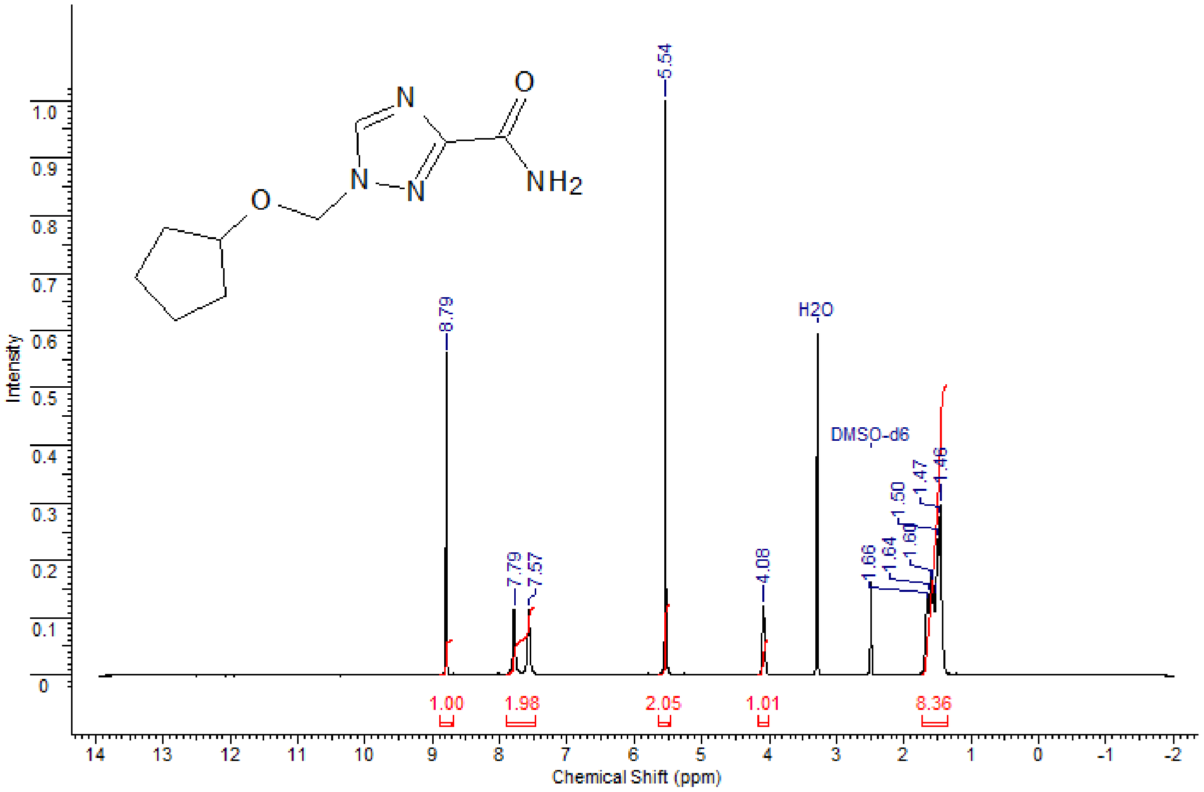

^{13}C NMR spectrum (**11i**)

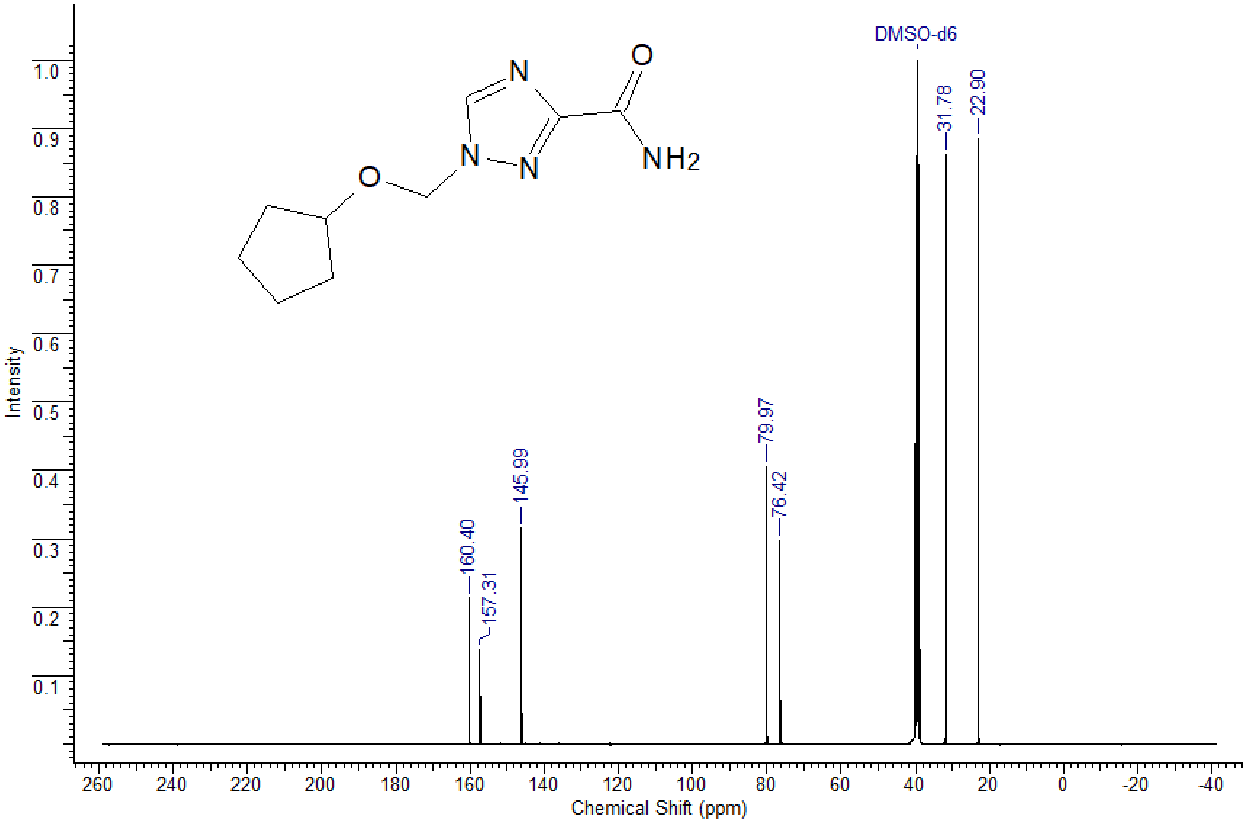

MS ESI+ (**11i**) (M=210 Da)

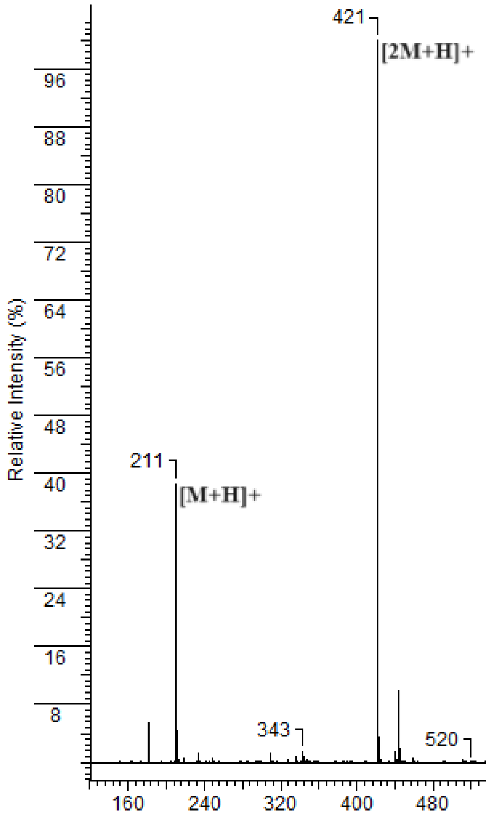

1-(Cyclohexyloxymethyl)-1,2,4-triazole-3-carboxamide (11j)

^{1}H NMR spectrum (**11j**)

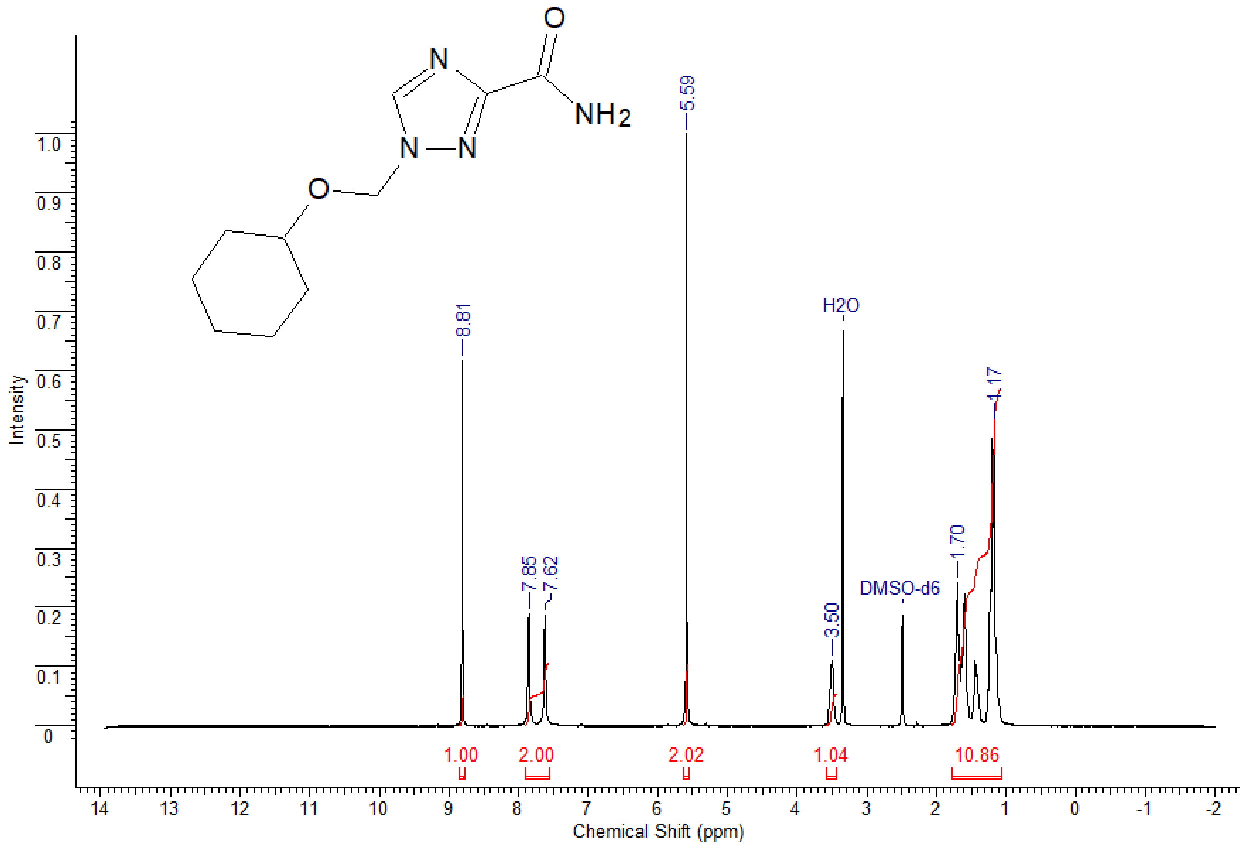

^{13}C NMR spectrum (**11j**)

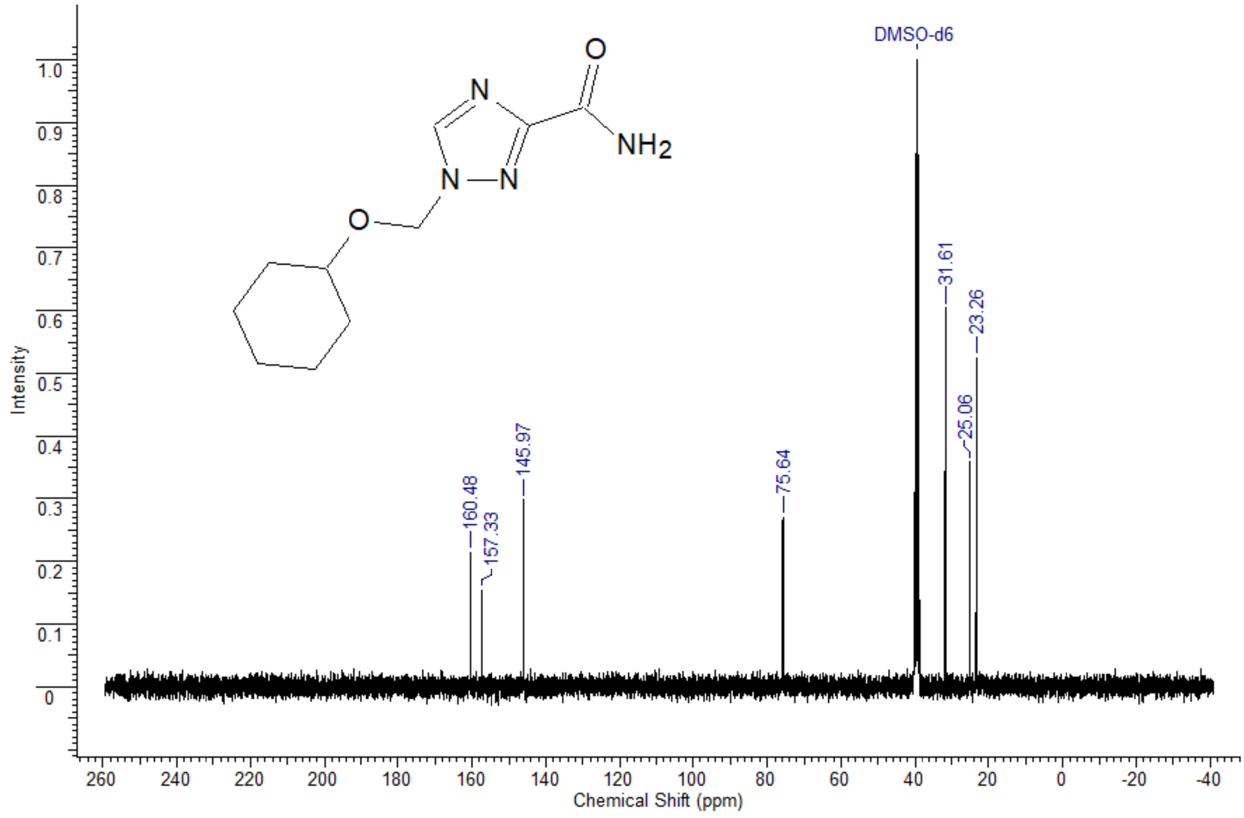

MS ESI+ (**11j**) (M=224 Da)

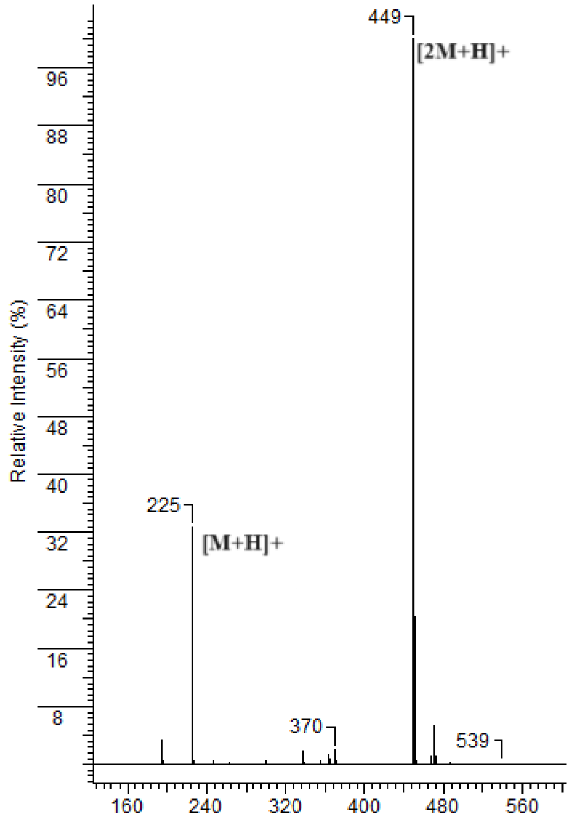

1-([2-Hydroxyethoxy]methyl)-1,2,4-triazole-3-carboxamide (1c)

^{1}H NMR spectrum (**1c**)

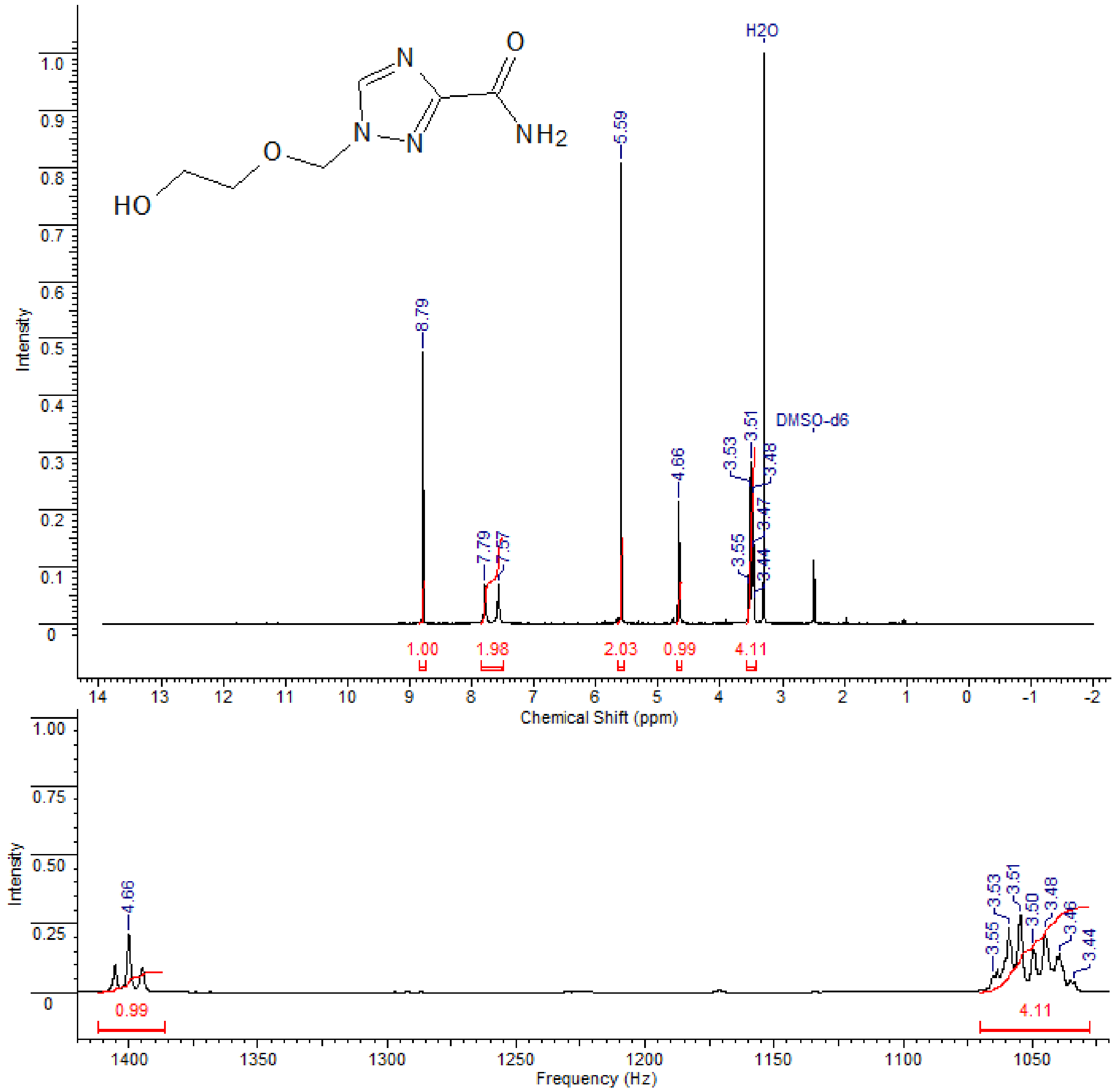

^{13}C NMR spectrum (**1c**)

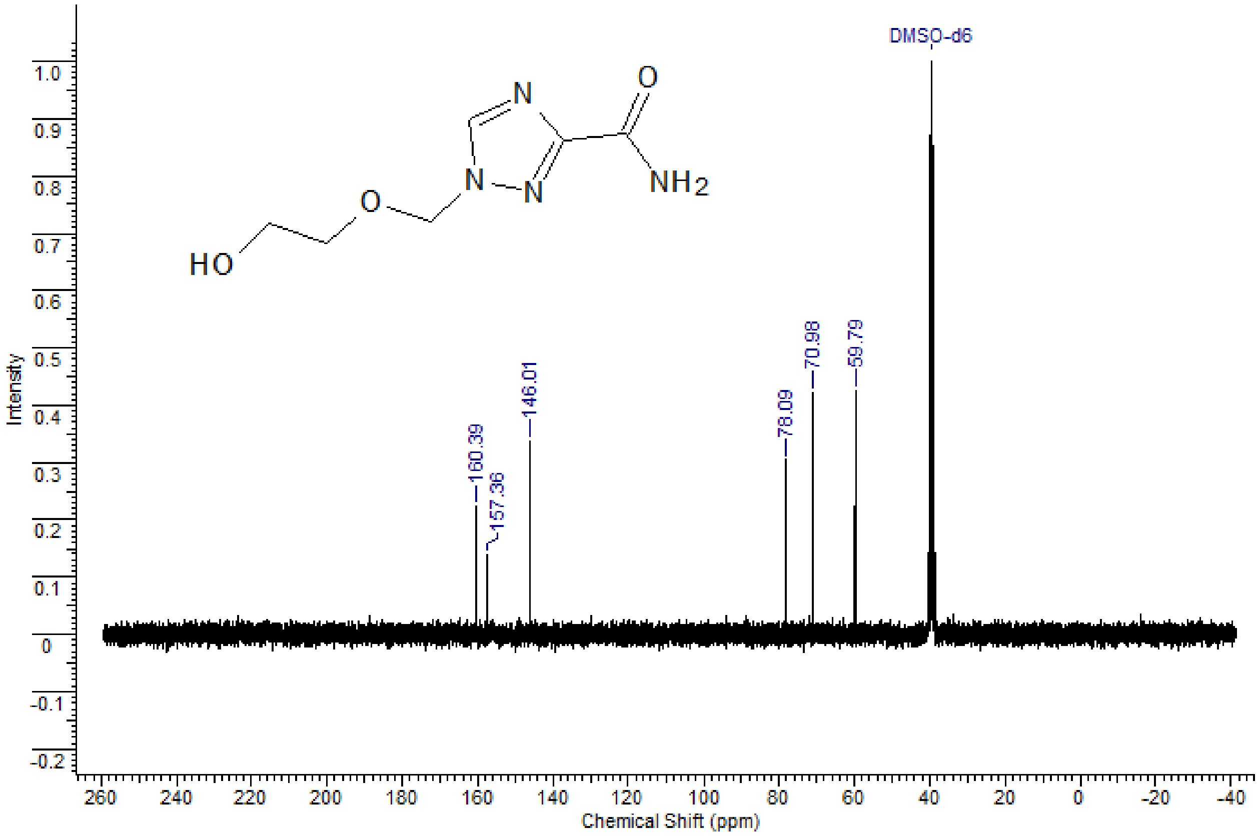

MS ESI+ (**1c**) (M=186 Da)

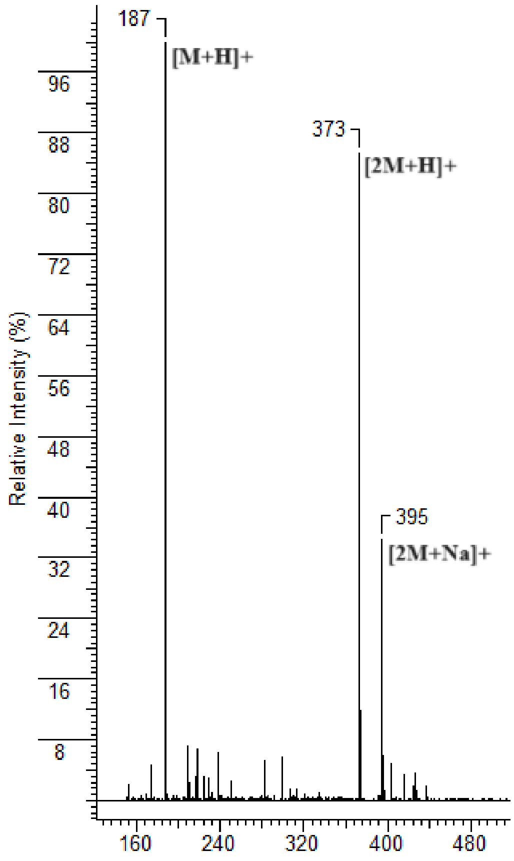

1-(Phenoxymethyl)-1,2,4-triazole-3-carboxamide (11k)

^{1}H NMR spectrum (**11k**)

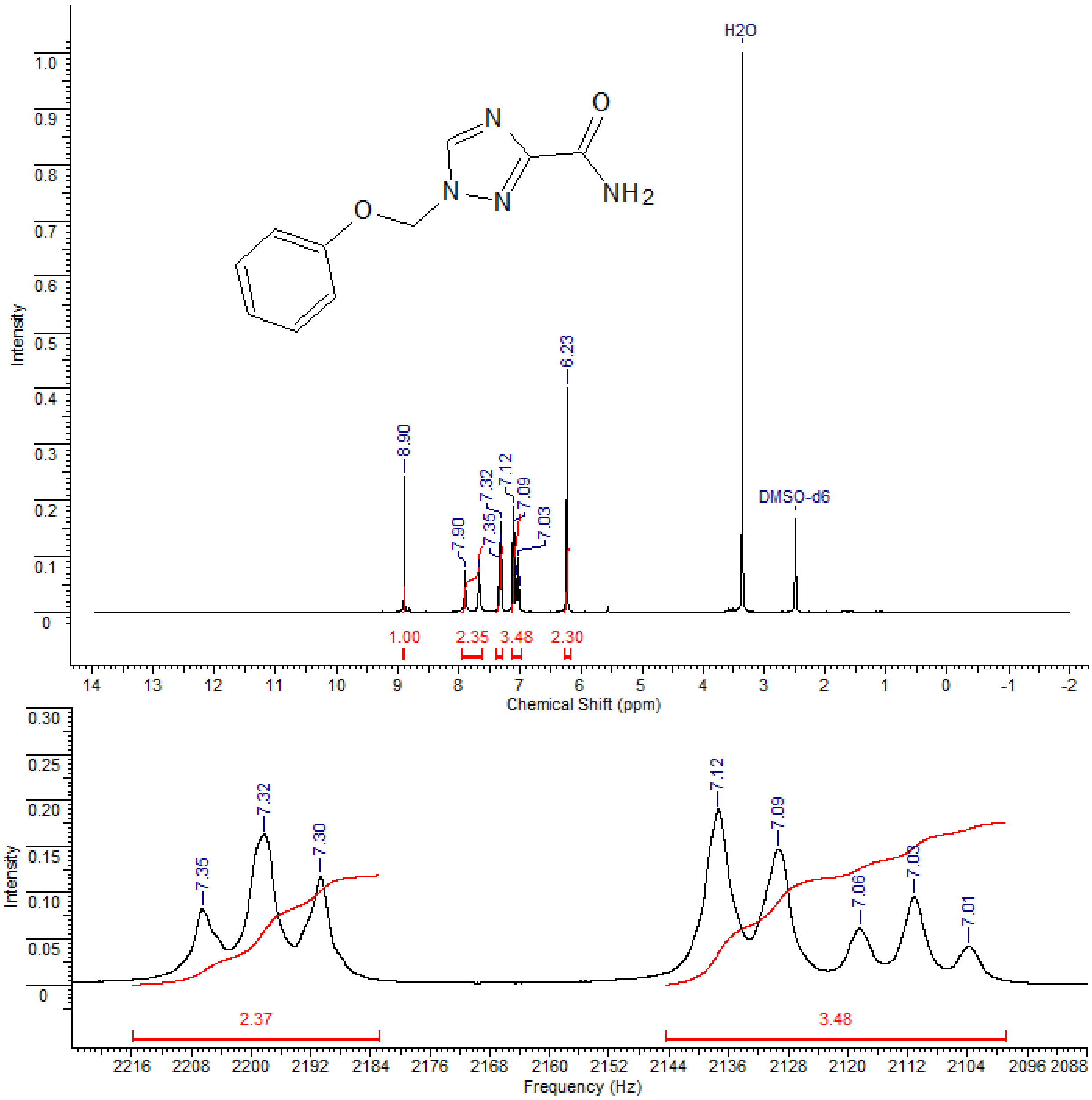

^{13}C NMR spectrum (**11k**)

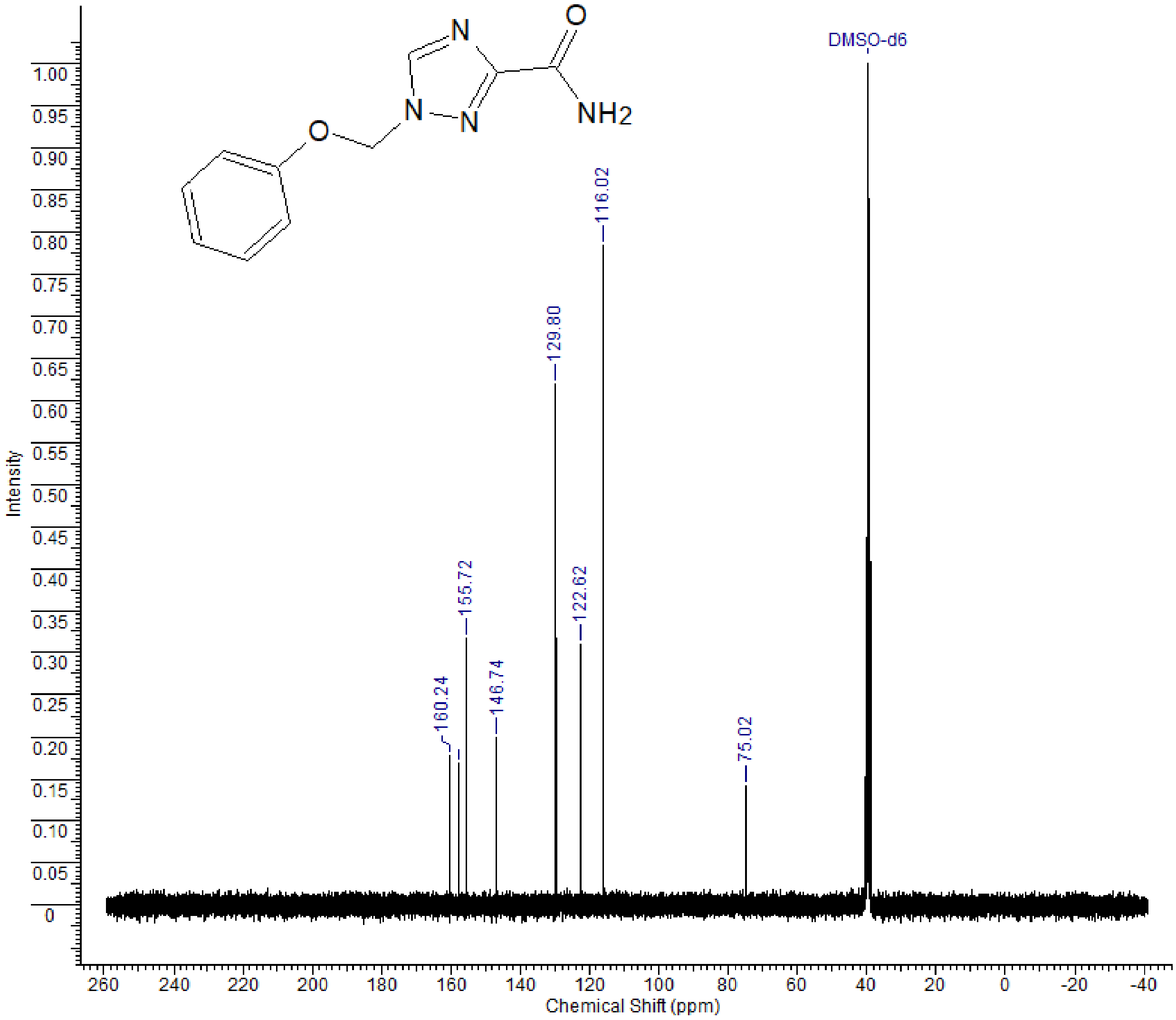

^{13}C APT NMR spectrum (**11k**)

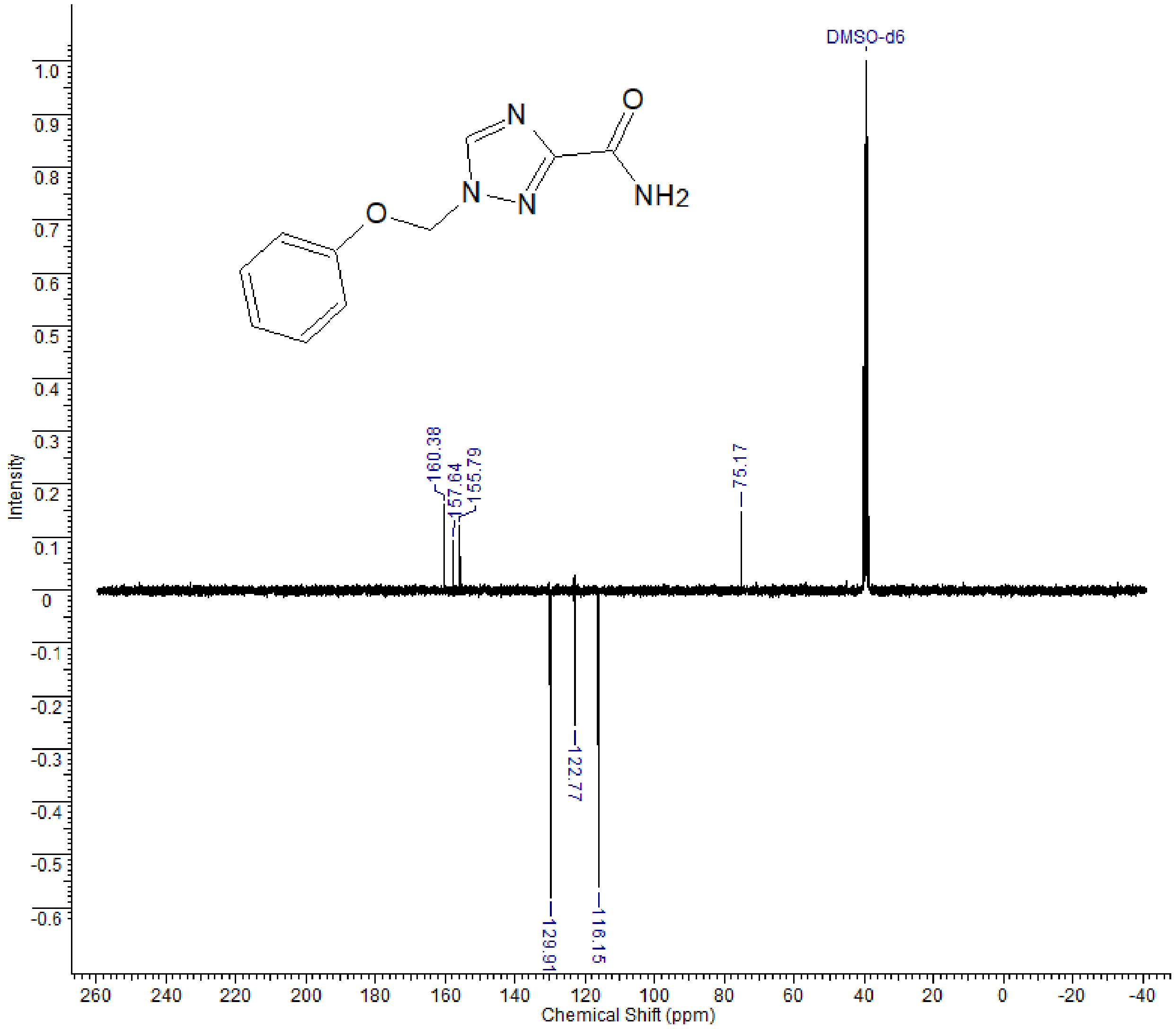

^{13}C DEPT-135 NMR spectrum (**11k**)

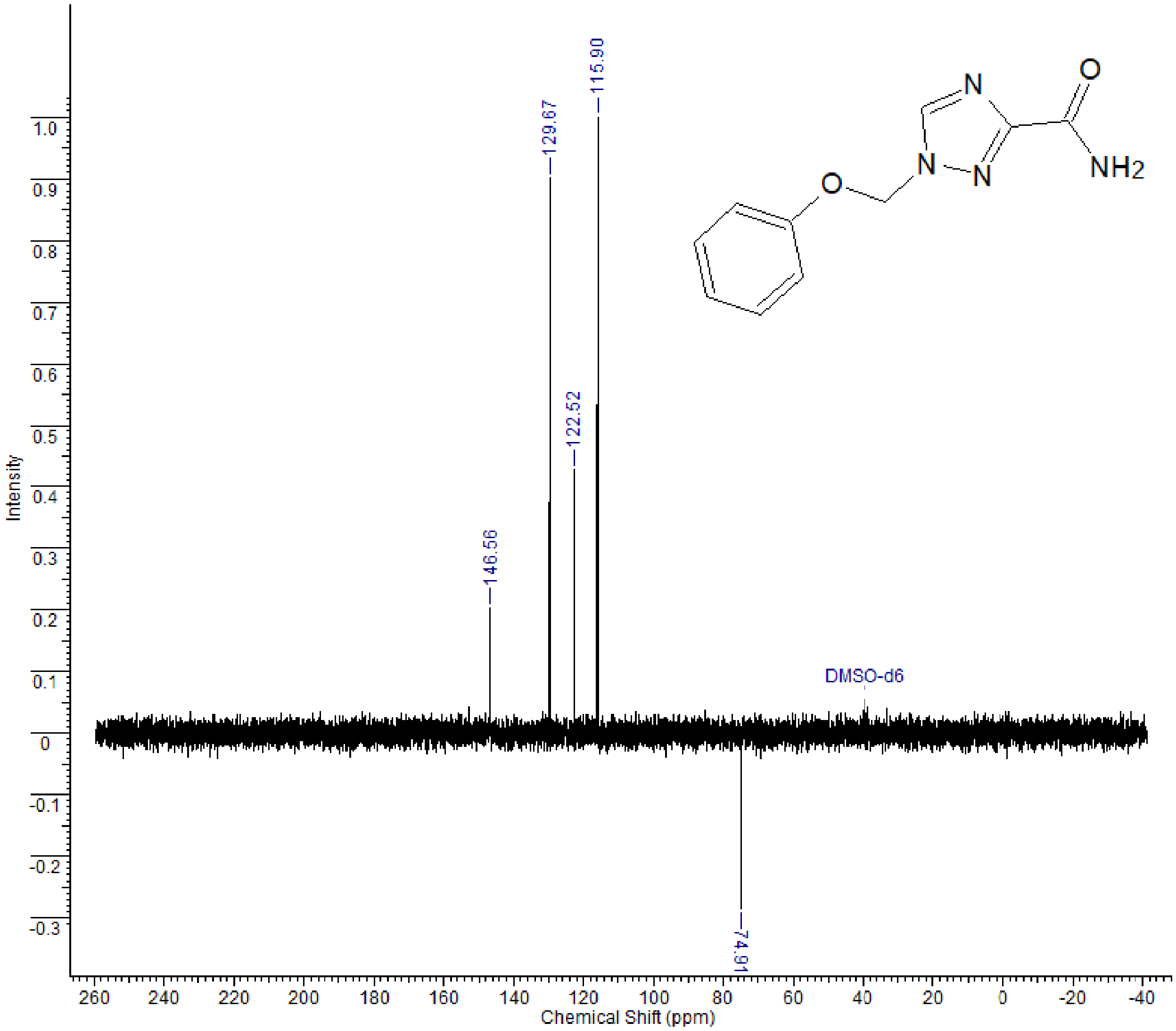

^{1}H-^{13}C HMBC NMR spectrum (**11k**)

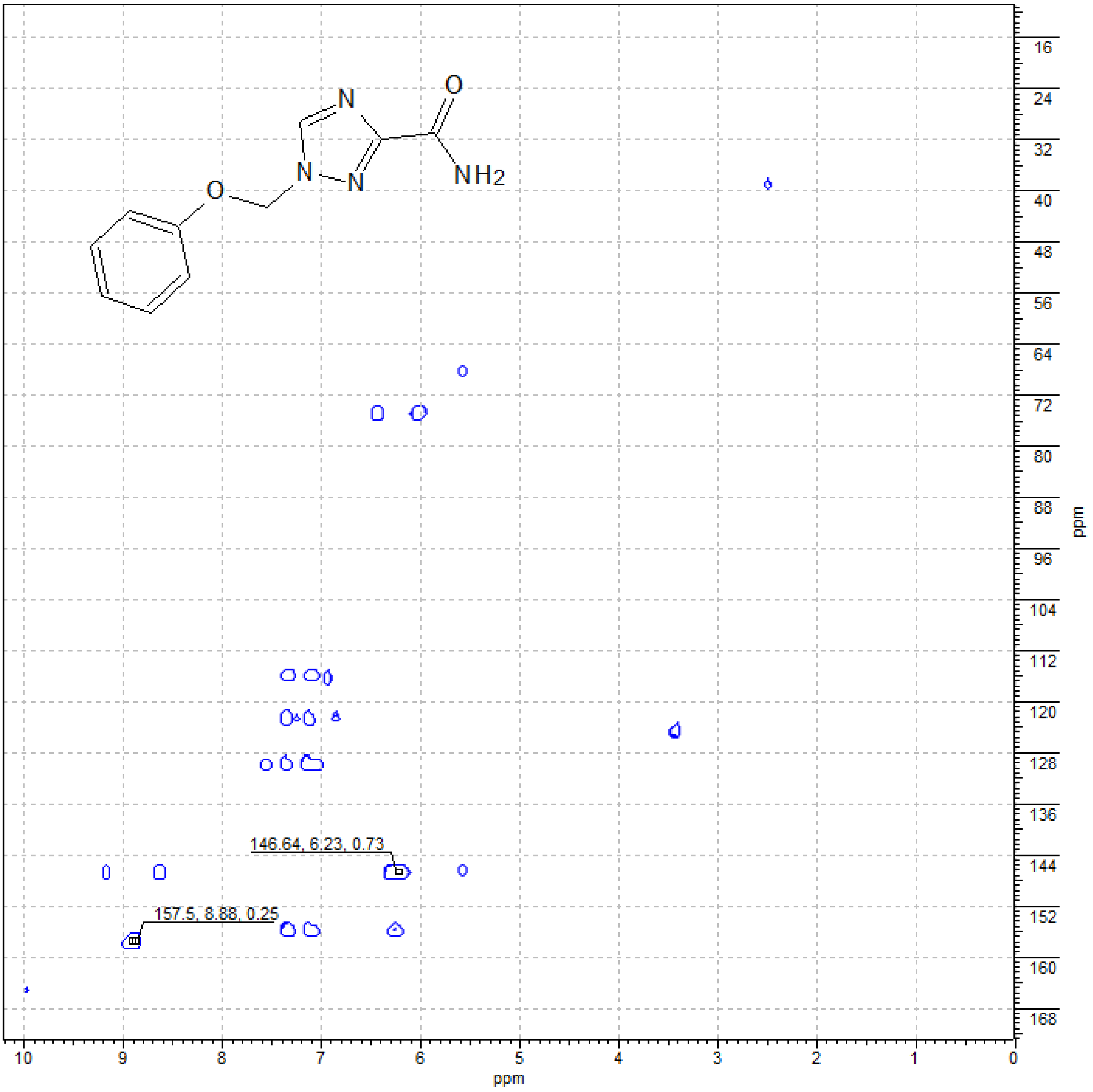

MS ESI+ (**11k**) (M=218 Da)

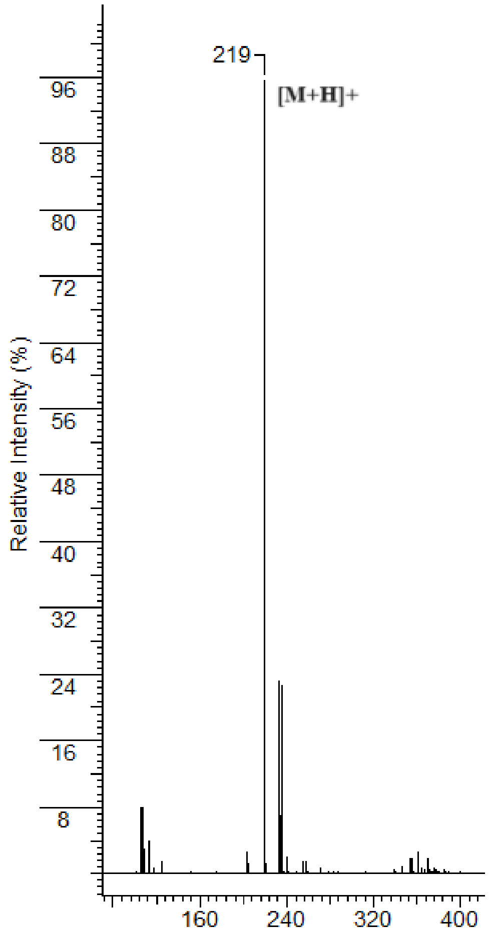

In Silico Studies

The crystal structure of eIF4E was obtained from the Protein Data Bank (PDB ID: 4TPW) as a co-complex of the translation initiation factor eIF4E with the inhibitor 4EGI-1. The selected structure of the complex has a resolution of 1.5 Å and does not contain gaps in the protein backbone near the ligand binding domain. Schrödinger Suite 2020 software (Schrödinger, Inc., New York, NY, USA; Maestro Version 12.5.139, MMshare Version 5.1.139, Release 2020-3) was used to perform the modelling. The re-moval of inhibitor and solvent molecules, addition of hydrogen atoms, assignment of atom types, combining of non-polar hydrogen atoms, and calculation of Gasteiger par-tial charges and Kollman charges were performed using Schrödinger Suite 2020 soft-ware (Schrödinger, Inc., USA) with the Schrödinger Maestro Protein Preparation Wiz-ard module. During the docking process, all torsional bonds of the ligands were free to rotate, while the protein remained rigid. The visualization and graphical representa-tion of ligand interaction results were performed using Schrödinger Maestro software.

Table S1. Binding parameters for compounds **1a,h,g, 2h,g, 6g, 11g**

S. No.	Contribution of the lipophilicity parameter					
	Calculated LogP	*DockScore (GScore)*	*ChemScore lipophilic pair term*	*ChemScore H-bond pair term*	*Electrostatic rewards*	*Sitemap terms*
**1a**	-2.26±0.68	-5.5	-0.5	-3.7	-0.8	-0.3
**1h**	-1.83±0.63	-3.5	-0.8	-1.2	-0.4	-0.3
**2h**	-1.70±0.63	-4.0	-0.8	-1.6	-0.8	-0.3
**1g**	-1.27±0.63	-3.1	-1.2	-1.2	-0.4	-0.3
**2g**	-1.13±0.63	-4.0	-1.1	-1.7	-0.9	-0.3
**6g**	3.25±0.64	-2.0	-0.6	-0.8	-0.7	-0.1
**11g**	3.12±0.64	-2.0	-1.9	-0.5	-0.6	-0.2
**4EGI-1**	4.54±0.67	-3.5	-2.8	-0.8	-0.5	0.0

Table S2. Possible binding modes in detail

S. No.	Relationship between ligand and protein	S. No.	Relationship between ligand and protein
**1a**	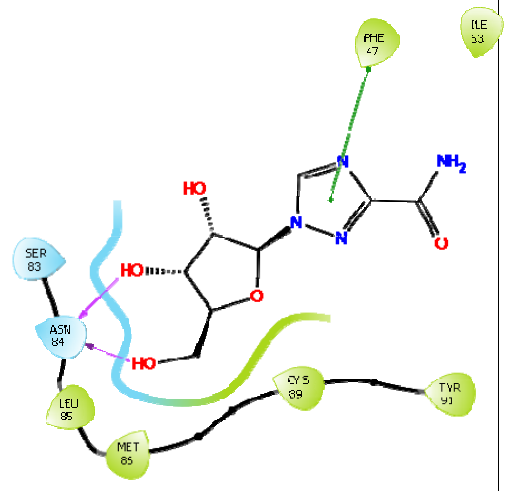	**4EGI-1**	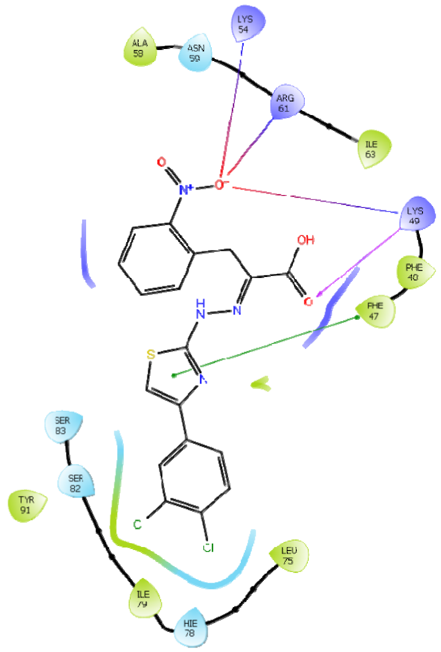
**1h**	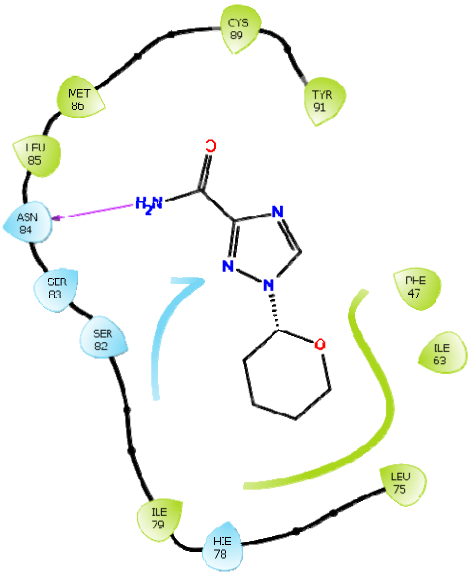	**2h**	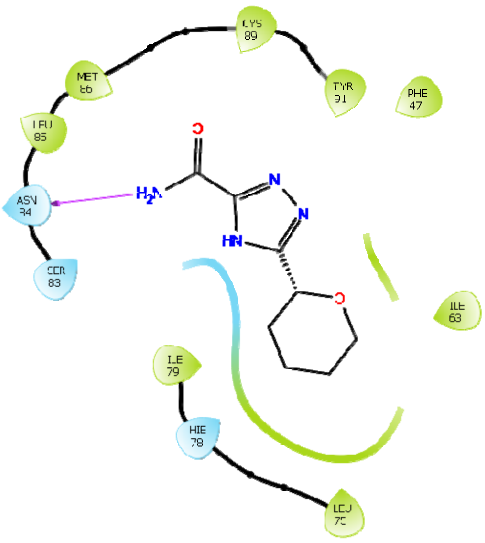

S. No.	Relationship between ligand and protein	S. No.	Relationship between ligand and protein
1g	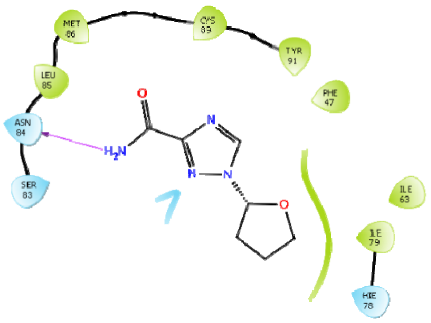	2g	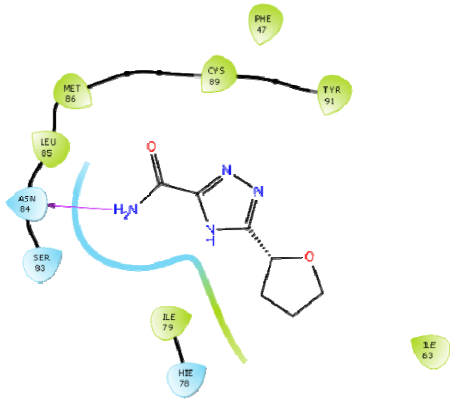
11g	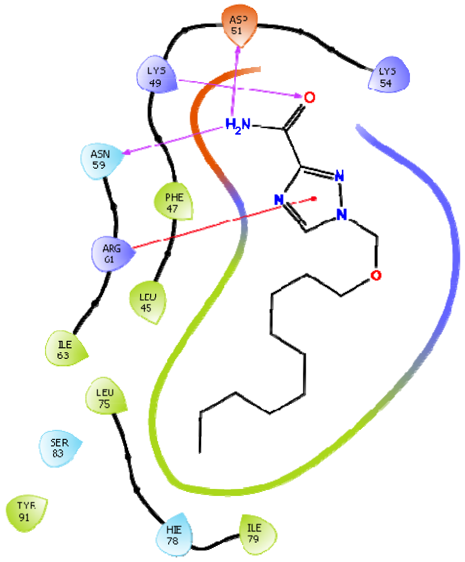	6g	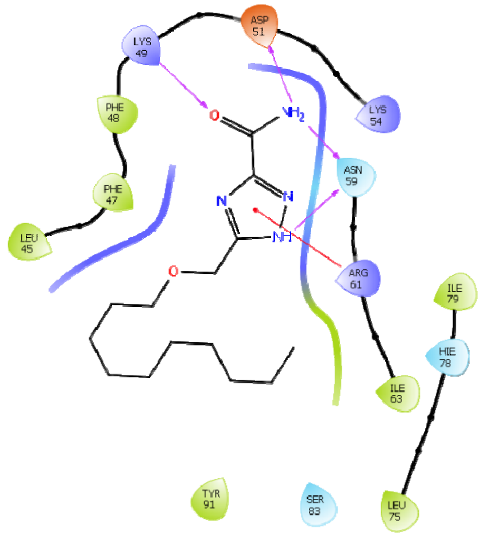

LID Legend

Charged (negative)	Polar	Distance	Pi-cation
Charged (positive)	Unspecified residue	H-bond	Salt bridge
Glycine	Water	Halogen bond	Solvent exposure
Hydrophobic	Hydration site	Metal coordination	
Metal	Hydration site (displaced)	Pi-Pi stacking	
